# Field‐Programmed Anisotropy in Magneto‐Piezoelectric Composites for Material‐Encoded Mechanoperception

**DOI:** 10.1002/adma.73967

**Published:** 2026-07-18

**Authors:** Yubin Kim, Yumin Kwon, Dabin Kim, Minsun Oh, Jin Young Jung, Dongyeong Gim, Kootak Hong, Jun Yeon Hwang, Sang‐Woo Kim, Minjeong Ha

**Affiliations:** ^1^ Department of Materials Science and Engineering Gwangju Institute of Science and Technology Gwangju Republic of Korea; ^2^ Department of Materials Science and Engineering Center for Human‐oriented Triboelectric Energy Harvesting Yonsei University Seoul Republic of Korea; ^3^ Institute of Advanced Composite Materials Korea Institute of Science and Technology Jeonbuk Republic of Korea; ^4^ Department of Materials Science and Engineering Chonnam National University Gwangju Republic of Korea

**Keywords:** anisotropy, decoupled mechanosensing, magnetic‐field alignment, piezoelectric composite, soft robotics

## Abstract

Decoupling superposed force vectors along spatially distinct axes is a prerequisite for artificial somatosensation in soft intelligent systems. Although polymer‐piezoelectric composites offer essential compliance, randomly distributed nanofillers result in a loss of inherent polar directionality and macroscopic anisotropy, which prevents force‐vector discrimination. Here, we address this challenge by encoding force‐mode selectivity into magneto‐piezoelectric composites via field‐programmed anisotropy. Guided by a rigorous torque‐balance framework, we achieve the deterministic spatial alignment of Fe_3_O_4_‐decorated BaTiO_3_ nanowires within a shape‐memory polymer matrix. The resulting axially aligned hierarchical percolation networks serve as continuous load‐transfer pathways to concentrate mechanical stress along the principal axes of the nanowires while suppressing off‐axis interference. Such structure‐driven anisotropy in electromechanical coupling maximizes piezoelectric transduction efficiency and empowers the nanocomposite to intrinsically distinguish force modalities. By upscaling the intrinsic anisotropy of piezoelectric nanowires to the macroscopic composite level, our approach defines a physical basis for material‐encoded mechanoperception. Our findings establish a versatile platform for soft embodied intelligence that offers vector‐resolved somatosensory capabilities at the material level.

## Graphical abstract

Field‐programmed anisotropy imparts material‐level intelligence to soft composites. Magnetic torque dynamics drive magneto‐piezoelectric nanowires from random dispersion into deterministic axial alignment. The resulting hierarchical percolation networks confine electromechanical coupling to specific axes and intrinsically decouple superposed force vectors. Consequently, vector‐resolved piezoelectricity establishes a physical basis for advanced artificial somatosensation.

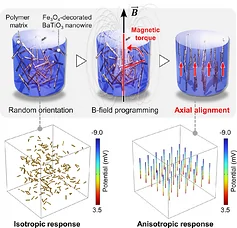

## 1 Introduction

Soft robotics, inspired by the adaptive nature of living organisms, marks a paradigm shift in autonomous machine design. By mimicking the mechanical compliance and dexterity of biological systems, soft robots can perform complex, multidirectional motions to navigate unpredictable environments [1, 2] and manipulate delicate objects [3, 4]. Yet, true autonomy requires more than compliant mechanics. It demands somatosensory capabilities comparable to biological mechanoreceptors that inherently decouple complex mechanical stimuli into distinct modalities [5, 6]. Such force‐mode selectivity enhances perceptual fidelity while reducing unnecessary data processing and energy consumption [6], which provides proprioceptive feedback [7], adaptive actuation [8], and context‐aware behavior in deformable intelligent systems [8, 9, 10]. Despite this critical need, current mechanosensors typically deliver magnitude‐only outputs without built‐in directional or modal specificity [11, 12, 13, 14]. Under multi‐axial and dynamic loading, this lack of vector selectivity leads to cross‐axis interference and spatial ambiguity. Although conventional force‐mode decoupling methods, such as multi‐sensor array configurations [15, 16], geometric isolation [17, 18], or computational post‐processing [11, 16, 19], can partially resolve modal crosstalk, they inevitably impose fabrication complexity, integration challenges, additional payload, and computational burdens that compromise both natural robotic motion and system‐level autonomy [11, 16, 19]_._

Piezoelectric materials offer a promising materials‐level strategy to overcome these challenges, as their tensorial electromechanical coupling allows for the direct transduction of mechanical stimuli into electrical signals with intrinsic orientation sensitivity. In crystalline piezoelectrics including lead‐based (e.g., PbTiO_3_, PZT) and lead‐free (e.g., BaTiO_3_, KNbO_3_) ceramics [20, 21], strong directional responses originate from tensorial piezoelectric coefficients that selectively couple applied stress to polarization along specific crystallographic axes. Nevertheless, the rigidity and brittleness of ceramic piezoelectrics severely limit their mechanical compatibility with soft and deformable platforms. Polymeric alternatives like polyvinylidene fluoride and its copolymers provide greater flexibility and mechanical resilience but exhibit weaker and isotropic piezoelectric responses due to incomplete dipole alignment and viscoelastic damping loss [22]. This trade‐off motivates composite designs that disperse crystalline piezoelectric nanofillers within elastomeric matrices, where the compliant host prevents ceramic fracture by redistributing mechanical loads and relaxing interfacial stress concentrations. In particular, embedding high‐aspect‐ratio piezoelectric nanowires further facilitates the efficient formation of percolated networks even at low volume fractions, which promotes preferential stress transfer along the nanowire long axis [23] where piezoelectric polarization is most efficiently generated. However, without deterministic alignment, the anisotropic polarization of individual nanowires is averaged out within elastomeric matrices, resulting in weak and non‐directional macroscopic piezoelectric responses [12, 14, 24, 25, 26]. To address this issue, various nanowire alignment technologies have been explored, such as sacrificial templating [27], extrusion‐based processing [25, 26, 28], substrate‐assisted growth [29, 30, 31], and dielectrophoretic assembly [24]. Despite these efforts, their applicability is largely restricted to planar geometries or uniaxial configurations. Achieving spatial control and long‐range ordering of nanowires within the viscous polymer matrices remains fundamentally challenging.

**FIGURE 1:**
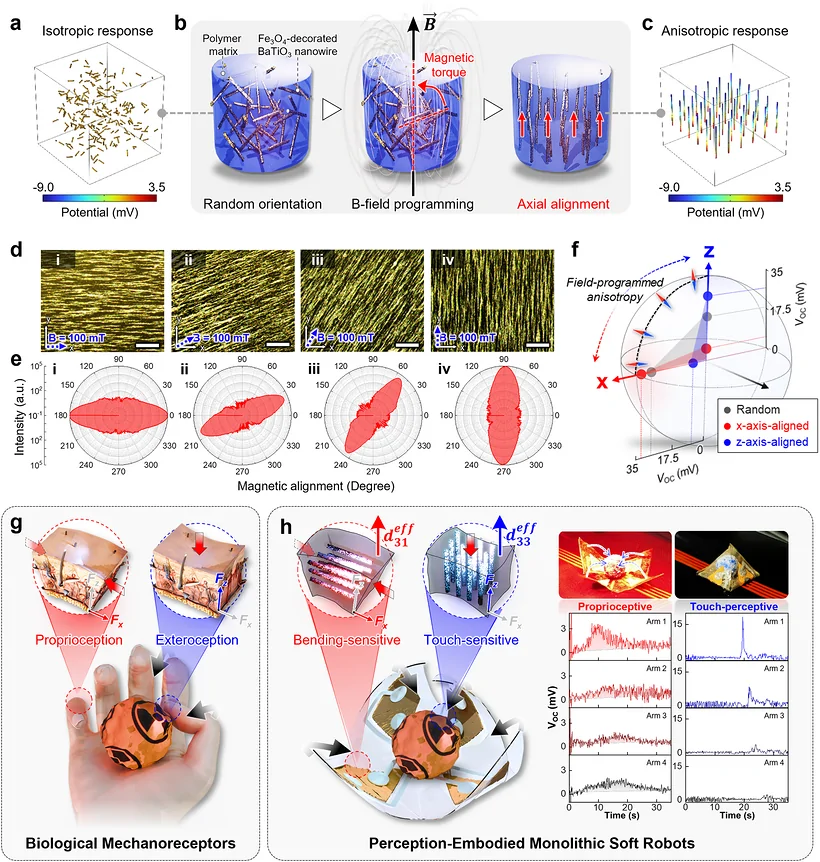
Field‐programmed anisotropy for material‐encoded mechanoperception in monolithic soft robots. (a–c) Concept of field‐programmed anisotropy. Finite element analysis (FEA) simulations contrast the low, isotropic potential distribution in composites with (a) randomly dispersed nanowires against the directionally coherent and (c) amplified response in axially aligned counterparts, (b) achieved via magnetic‐torque‐driven alignment dynamics. (d) Optical microscopic (OM) images and (e) corresponding polar plots of nanowire orientation profiles prepared under varying magnetic field angles (0–90°), Scale bar: 50 µm. (f) Three‐dimensional representation of the vector‐resolved open‐circuit voltage (*V*_oc_) signal profiles demonstrating the tunable anisotropy. The x, z‐axis aligned nanowires (red and blue spheres) exhibit distinct directionality compared to the random counterpart (gray sphere), enabling selective response to specific force vectors. (g) Bio‐inspired mechanoperception strategy mimicking skin mechanoreceptors and (h) its implementation in a monolithic soft robot. The structure‐driven anisotropy allows the robot to autonomously distinguish between bending‐induced (proprioceptive) and compression‐induced (exteroceptive) signals during closed‐loop tasks.

In this work, we present a strategy for field‐programmed anisotropy in magneto‐piezoelectric composites that effectively bridges the intrinsic polar directionality of piezoelectric nanowires to the macroscopic composite level (Figure 1a–c). We derive a rigorous torque‐balance framework to govern the rotational dynamics of Fe_3_O_4_ nanoparticle‐decorated BaTiO_3_ nanowires within a viscous shape‐memory polymer matrix. The magnetic‐field‐guided approach dictates the deterministic spatial alignment of magneto‐piezoelectric nanowires along arbitrary axes and drives their convergence into confined, geometry‐defined configurations regardless of their initial orientation (Figure 1d,e). These axially aligned hierarchical percolation networks facilitate preferential stress transfer along the longitudinal direction of the nanowires, thereby amplifying axial piezoelectric responses while minimizing orthogonal crosstalk (Figure 1a,c,f). Such structure‐driven anisotropy allows the composite to function as a material‐level filter that physically separates complex force modalities without computational intervention, which is unattainable in systems with randomly dispersed nanowires. We highlight the versatility of this platform by designing a self‐proprioceptive and shape‐morphing robot where the composite serves as a monolithic sensing‐actuation unit for in‐body transduction without discrete sensor modules or complex system‐level integration. Crucially, since the alignment process operates effectively within viscoelastic shape‐memory polymers, nanowires can be positioned directly at mechanically active sites and oriented along motion‐relevant axes. We demonstrate that this material‐encoded mechanoperception enables vector‐resolved somatosensation, which allows the soft robot to autonomously encapsulate hazardous objects through real‐time, closed‐loop feedback (Figure 1g,h). This work establishes a versatile material platform for adaptive physical intelligence, supporting robust force readout and autonomous task execution of soft intelligent systems in inaccessible environments.

## 2 Results and Discussion

### 2.1 Synthesis and Field‐Directed Ordering of Magneto‐Piezoelectric Nanowires

**FIGURE 2:**
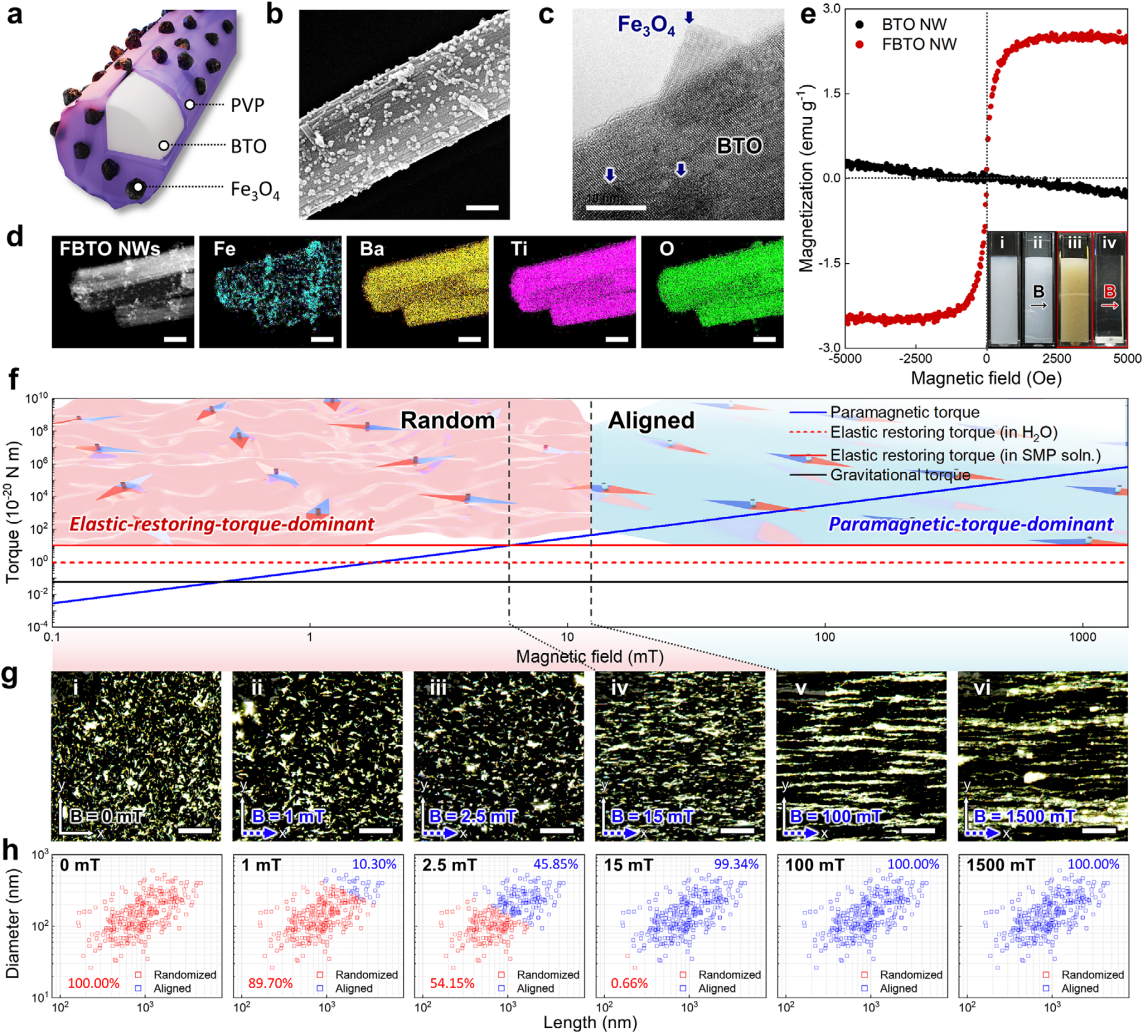
Synthesis and magnetic field‐directed alignment of Fe_3_O_4_ NP‐decorated BaTiO_3_ nanowire (FBTO NW). (a) Schematic illustration of FBTO NW with the PVP‐mediated growth of Fe_3_O_4_ NPs on the BTO surface. (b–d) Structural characterization of FBTO NWs. (b) High‐resolution scanning electron microscopic (HR‐SEM), Scale bar: 200 nm, (c) high‐resolution transmission electron microscopic (HR‐TEM) showing the lattice interface, Scale bar: 10 nm, and (d) energy‐dispersive X‐ray spectroscopic (EDS) mapping images obtained from scanning transmission electron microscopy (STEM) of FBTO NW, Scale bar: 200 nm. (e) Magnetic hysteresis (*M*‐*H*) loops of BTO (black) and FBTO (dark red) NW. Insets compare the magnetic response of (i, ii) the BTO and (iii, iv) FBTO NW suspensions in the presence of a permanent magnet (*B*). (f) Theoretical torque analysis comparing the paramagnetic torque (blue line) against elastic restoring by aqueous medium (red dotted line), elastic restoring by shape‐memory polymer (SMP) solution (red solid line), and gravitational torques (black line), defining the alignment regimes. (g) OM images of FBTO NWs/SMP composites fabricated under increasing magnetic field strengths (0 – 1500 mT). Scale bar: 20 µm. (h) Calculated phase diagrams showing the population of randomized (red) versus aligned (blue) FBTO NWs based on aspect ratio and field strength in SMP solution.

Magneto‐piezoelectric nanowires were prepared through a two‐step process involving the hydrothermal growth of BaTiO_3_ nanowires (BTO NWs) followed by surface decoration with Fe_3_O_4_ nanoparticles (NPs), as shown in Figure 2a and Figure S1. BTO NWs were synthesized via a nano‐templating‐assisted hydrothermal method. TiO_2_ NPs first reacted with NaOH to form Na_2_Ti_3_O_7_ NWs, which served as sacrificial templates for ion‐exchange reactions. Acidic treatment replaced Na^+^ ions with protons, after which Ba^2+^ incorporation induced the formation of crystalline BTO NWs with a high aspect ratio (AR). To magnetize BTO NWs, the BTO surface was further modified with poly(vinylpyrrolidone) (PVP) to provide nucleation sites for the growth of Fe_3_O_4_ NPs. Subsequently, co‐precipitation of Fe^2+^ and Fe^3+^ ions under basic and anaerobic conditions led to uniform deposition of Fe_3_O_4_ NPs on the PVP‐functionalized BTO surface, resulting in the successful synthesis of Fe_3_O_4_ NPs‐decorated BTO (FBTO) NWs (see Methods for details). The morphology of FBTO NWs synthesized using 5% (mol/mol) Fe precursors relative to BTO NWs can be clearly verified in Figure 2b and Figure S1b. Both pristine and FBTO NWs exhibited similar dimensions, with an average length of approximately 1.04 µm and a diameter of 150 nm, corresponding to an AR of 7.6. Despite the inherent polydispersity, the morphological characteristics were consistently maintained after functionalization (Figure S1c,d). FBTO NWs displayed noticeably rougher surfaces than pristine BTO NWs due to the dense coverage by polyhedral Fe_3_O_4_ NPs (Figure 2b,c and Figure S1f). Increasing the Fe precursor concentration resulted in larger Fe_3_O_4_ NPs and higher surface coverage, consistent with their Fe‐based origin (Figure S2). Elemental mapping by scanning transmission electron microscopy coupled with energy‐dispersive X‐ray spectroscopy (STEM–EDS) confirmed Fe localization at the NPs on the BTO surface, while Ba, Ti, and O remained uniformly distributed throughout the core (Figure 2d). Given that the magnetic properties of Fe_3_O_4_ NPs are highly dependent on their crystallographic phase and particle size [32], the phase composition of Fe_3_O_4_ NPs on the BTO surface was investigated. X‐ray diffraction (XRD) analysis revealed a distinct (311) reflection at 35.4°, characteristic of inverse spinel Fe_3_O_4_, along with major reflections at 22.1° and 45.1°, corresponding to the (100)/(001) and (200)/(002) planes of tetragonal BTO [25], respectively (Figure S3a). Notably, the intensity of the Fe_3_O_4_ (311) peak increased with higher Fe precursor concentrations, indicating enhanced NP loading (Figure S3b). Selected‐area electron diffraction (SAED) patterns acquired from TEM further supported these findings by exhibiting diffraction patterns consistent with both BTO and Fe_3_O_4_ phases (Figure S4). Fourier‐filtered imaging indexed to the (311) and (220) planes confirmed the presence of crystalline Fe_3_O_4_ NPs localized on the BTO surface (Figure S4e), clearly distinct from the underlying BTO lattice.

To unveil the growth mechanism of Fe_3_O_4_ NPs on the PVP‐functionalized BTO NWs, X‐ray photoelectron spectroscopy (XPS) analysis was conducted on both pristine BTO and FBTO NWs (Figure S5). In the pristine BTO, the Ba 3d and Ti 2p spectra corresponded to Ba^2+^ and Ti^4+^ in the perovskite lattice, with minor signals from under‐coordinated Ba and Ti states (Figure S5a‐i,b‐i). After Fe_3_O_4_ NPs growth, the shoulder peaks associated with Ba vacancies and the Ti^3+^ subpeaks in the Ba 3d and Ti 2p spectra became more pronounced, which reflects a higher density of under‐coordinated surface sites that promoted Fe nucleation (Figure S5a‐ii,b‐ii). Even before Fe incorporation, PVP‐functionalized BTO NWs exhibited stronger Ba 3d and Ti 2p peaks associated with coordinatively unsaturated states (Figure S5e–h) [33]_._ This indicates that PVP could promote mild surface reduction and generate Ba or Ti vacancies that served as preferential anchoring sites for Fe ion adsorption. Under acidic conditions ($pK_{a\;FeCl_{3}}$≈ 2.4), the vacancy sites were saturated with oxygen‐related moieties through chemisorption of H_2_O, OH^−^, or O_2_. This process effectively terminated the surface with hydroxyl groups [34]_._ Subsequent pH elevation to ∼11 using NH_4_OH deprotonated these hydroxyl groups, thus creating negatively charged surface sites that electrostatically promoted adsorption of Fe^3+^ and Fe^2+^ ions. The adsorbed Fe ions then participated in co‐precipitation, leading to the nucleation and growth of Fe_3_O_4_ NPs on the BTO NWs. Zeta potential measurements further support the surface‐mediated growth mechanism. PVP‐functionalized BTO NWs became more negatively charged after acid treatment (from −38.7 to −42.4 mV) and subsequently shifted toward near neutrality (−29.8 mV) upon Fe_3_O_4_ NP formation, consistent with surface charge compensation through Fe cation adsorption (Figure S6a,b). Specifically, FBTO NWs synthesized in the presence of PVP exhibited a greater depletion of surface‐charged groups and markedly more homogeneous Fe_3_O_4_ NPs coverage than those prepared without PVP (Figures S6c,d and S7). These results confirm that PVP enhanced interfacial coordination and promotes uniform surface functionalization (Figure S7b). The appearance of Fe─O bonding features and Fe 2p signals in the XPS spectra further verifies the successful formation of Fe_3_O_4_ NPs on the BTO surface (Figure S5c,d) [35]_._ In spite of the defective features found in FBTO NWs, ferroelectric properties such as local piezoelectric responses showed comparable results involving ferroelectric domain switching behaviors near a coercive field (Figure S1e–h) [31]_._

The functionalization with Fe_3_O_4_ NPs imparts magnetic responsiveness to the BTO NWs, which was primarily evidenced by magnetic hysteresis measurements in Figure 2e. Unlike the pristine BTO NWs that exhibited a diamagnetic response, the FBTO NWs showed negligible coercivity and a saturation magnetization of 2.26 emu g^−^^1^, in good agreement with their nominal Fe:Ba stoichiometry (5%). The near‐zero remanent magnetization (∼0.14 emu g^−^^1^ at zero field) further highlights the superparamagnetic nature of FBTO NWs, which is beneficial for minimizing magnetic dipole–dipole interactions and preventing irreversible agglomeration in the absence of an external magnetic field. The inset images in Figure 2e demonstrate that BTO NWs are merely dispersed near a permanent magnet (i, ii), whereas FBTO NWs migrate under the field (iii, iv). The macroscopic magnetic control arises from the synergy between superparamagnetic Fe_3_O_4_ NPs and the high‐AR BTO NWs, which facilitated efficient magnetic alignment. Although Fe_3_O_4_ NPs lack shape anisotropy, their axial arrangement along the elongated BTO backbone reduces demagnetization [36] and induces rotation of the entire NW along the applied field direction [37]. This alignment minimizes the magnetic potential energy in the system, as magnetic dipole moments preferentially orient parallel to the field (Figure S8).

To validate the precise structural ordering enabled by the magnetic responsiveness, we analyzed the alignment behavior of FBTO NWs in viscous media based on the relative magnitudes of paramagnetic, elastic restoring, and gravitational torques. The balance among these contributions dictates the transition from random to aligned states across media of different viscosities and magnetic field strengths (see more details in Supplementary Note 1). FBTO NWs dispersed in water (0.01% w/v), where the shear modulus is negligible (*G*_H2O_ ≈ 10^−8^ MPa), were readily aligned along applied in‐plane magnetic fields (*θ* = 0° or 180°) and exhibited sharp angular distribution profiles centered at 0° and 180° (Figure S9a,b). The magnetic alignment was further demonstrated by embedding FBTO NWs into a viscous solution of polyether‐polyol polyurethane‐based shape memory polymers (SMP). In aqueous media, magnetic alignment initiated at approximately 2.5 mT as the magnetic torque (1.81 × 10^−20^ N·m) exceeded the elastic restoring torque (8.99 × 10^−21^ N·m). In contrast, the SMP solution exhibits a higher elastic resistance (1.04 × 10^−^^19^ N·m), nearly an order of magnitude greater than that observed in the aqueous environment. Thus, the theoretical onset of alignment is predicted to shift toward a higher magnetic field of 6.0 mT (Figure 2f and Figures S9d–f and S10a). Guided by these numerical calculations, the magnetic alignment in the viscous medium was experimentally validated (Figure 2g,h). Moreover, while the behavior of the majority of FBTO NWs aligned with the theoretical estimation, we further described the NW population distribution under magnetic fields to provide a more comprehensive understanding of alignment dynamics in both low‐ and high‐viscosity media (Figure 2h and Figure S9c). The ensemble behavior confirms fully direction‐selective ordering and verifies vectorially programmable alignment with high fidelity. Remarkably, the field‐programmed alignment was adaptable across polymer matrices with wide viscosity ranges and even in systems whose rheological properties changed during processing (Figures S10b and S11). Such consistency highlights the strong field responsiveness of FBTO NWs and ensures their suitability for directed assembly in versatile processing environments. Our magnetic‐field‐assisted strategy presents fundamental advantages for structurally organizing anisotropic fillers within viscous polymer matrices. Because magnetic fields inherently permeate dense and highly viscous polymers without attenuation, this guided approach is completely contactless and electrode‐independent. Consequently, it provides infinite degrees of freedom to dynamically reorient and achieve high‐resolution spatial alignment along arbitrary 3D axes. Ultimately, such spatial versatility establishes a highly scalable pathway for fabricating complex 3D composite architectures without introducing geometric constraints or parasitic interfaces.

### 2.2 Hierarchical Ordering and Anisotropic Polarization of Magneto‐Piezoelectric Composites

**FIGURE 3:**
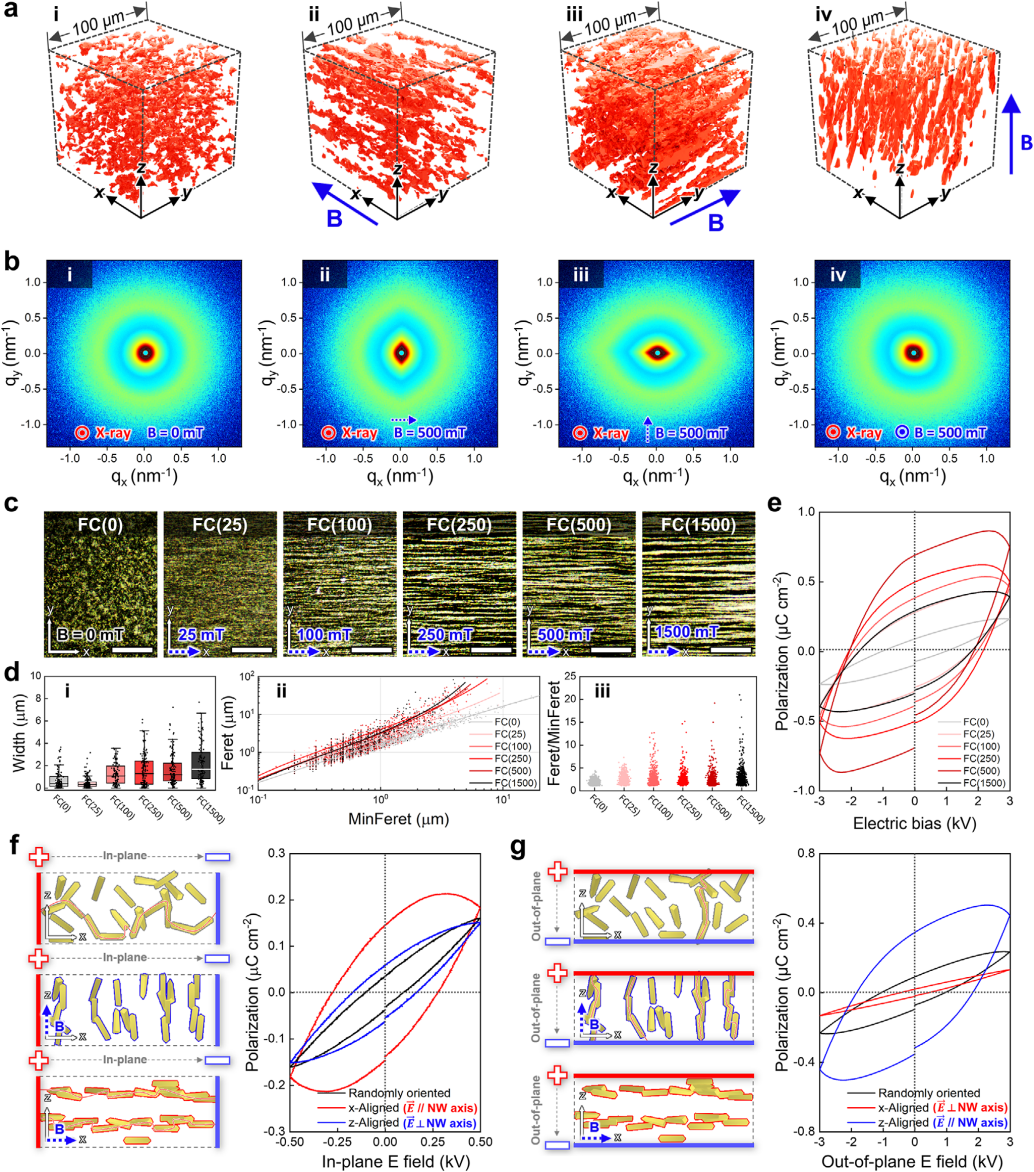
Structural anisotropy and direction‐dependent ferroelectricity of aligned FBTO NWs/SMP composites. (a,b) Three‐dimensional structural analysis confirming axial alignment. (a) X‐ray microscopic (XRM) images (volume: 100 µm × 100 µm × 100 µm). (b) Two‐dimensional small‐angle X‐ray scattering (2D‐SAXS) patterns. Panels display (i) random orientation and alignment along the (ii) x‐, (iii) y‐, and (iv) z‐axes, directed by the magnetic field direction (blue arrows). (c,d) Evolution of alignment degree with field strength. (c) OM images of composites fabricated under magnetic fields ranging from 0 to 1500 mT. Each are denoted as FC(X), where X indicates the applied field strength in mT. Scale bar: 50 µm. (d) Statistical analysis of NW assemblies derived from OM data: (i) chain width variation, (ii) correlation between Feret and MinFeret diameters, and (iii) aspect ratio distributions for the FC series. (e) Ferroelectric polarization‐electric field (*P*‐*E*) hysteresis loops for FC(0) through FC(1500), showing alignment‐enhanced polarization. (f,g) Anisotropic ferroelectric responses. Schematics (left) and *P*‐*E* loops (right) represent the relationship between NW alignment and the electric field vector (*E*). (f) In‐plane and (g) out‐of‐plane *E*‐field configurations with curves comparing randomly oriented (black), x‐axis‐aligned (red), and z‐axis‐aligned (blue) states.

Through the field‐directed microscale alignment of high‐AR FBTO NWs, we clarified how hierarchical structural ordering governs the physical anisotropy of bulk solid‐state magneto‐piezoelectric composites. The composites were first fabricated by uniformly dispersing 5 wt% of FBTO NWs in the SMP matrix, followed by magnetic‐field alignment along the x‐, y‐, or z‐axis during solidification. 3D X‐ray microscopy (XRM) revealed well‐defined internal architectures in which the FBTO NWs remained uniformly distributed and preserved their programmed alignment in Figure 3a and Figure S12. These structural arrangements were further supported by 2D small‐angle X‐ray scattering (SAXS), which revealed alignment‐dependent scattering profiles across the composites (Figure 3b). Specifically, x‐ and y‐axis‐aligned NW composites displayed streak‐like scattering features when the incident beam was perpendicular to the alignment axis (Figure 3b‐ii, iii), whereas the random‐ and z‐axis‐aligned NW composites showed isotropic scattering owing to the absence of lateral orientation and alignment parallel to the beam direction (Figure 3b‐i, iv). To evaluate how hierarchical ordering influences piezoelectric polarization, the FBTO NWs content was increased to 40 wt%. At this high loading, the FBTO NWs not only exceeded the threshold for field‐directed alignment but also experienced stronger magnetophoretic interactions that led to the chain‐ and bundle‐like assemblies under an applied magnetic field (Figure 3c). The dependence of the assembled structures on magnetic‐field strength was quantified by analyzing lateral dimensions, including chain width normal to the field direction as well as the Feret and MinFeret diameters that represent maximum and minimum projected lengths. Systematic broadening of chain width appeared with increasing field strength from 100 to 1500 mT (Figure 3d‐i; denoted as FC(X), where X means the field strength), which indicates tighter axial packing accompanied by lateral growth. The Feret diameter increased more strongly than the MinFeret diameter, resulting in a broader and enhanced asymmetric distribution (Figure 3d‐ii). The observed microstructural evolution originates from the strong dipolar interactions between FBTO NWs aligned along the external field directions, where significant magnetic permeability contrast arises between Fe_3_O_4_ NPs and the surrounding polymer matrix [38, 39]. Because the local field gradient strengthened magnetophoretic attraction, it drove a hierarchical transition from isolated NW alignment to chain formation at intermediate fields (100 mT) and ultimately bundle‐like assemblies at the highest field (1500 mT). These morphological behaviors were universally observed regardless of the NW loadings (Figure S13a,b). However, once axial packing was saturated within the aligned domains at high NW loadings, the excess NWs assembled laterally to form secondary branches oriented perpendicular to the applied field (Figure S13b).

Polarization‐Electric field (*P‐E*) hysteresis measurements further revealed how such hierarchical ordering modulates the ferroelectric polarization state of the composites, which fundamentally dictates their electromechanical response. The remanent polarization (*P*_r_) of the z‐axis‐aligned NW composites increased from 0.08 to 0.58 µC cm^−2^ between FC(0) and FC(500). The significant enhancement is attributed to the improved poling efficiency facilitated by the continuous and field‐oriented FBTO NW pathways that act as high‐permittivity channels. However, a subsequent decline in *P*_r_ to 0.41 µC cm^−2^ at FC(1500) suggests that over‐densified NW assemblies and multidirectional branching disrupt the uniaxial percolation network, which increases interfacial charge loss and limits macroscopic polarization (Figure 3e and Figure S13e). To verify anisotropy associated with NW alignment, polarization responses were measured under electric field applied either parallel or perpendicular to the alignment axis (Figure 3f,g and Figure S13c,d). When the electric field was applied parallel to the NW direction, polarization was strongly amplified due to the preferential dipole moment along the aligned and high‐permittivity NW network. In contrast, electric fields applied orthogonal to the alignment direction traverse a larger fraction of the low‐permittivity polymer matrix and intersect discontinuous NW segments, resulting in a reduced net polarization. Overall, these results show that the polarization enhancement was maximized when NW chains formed continuously percolated and field‐collinear pathways. Over‐densified and multidirectional branching weakened this axial continuity, indicating that the polarization response is governed by the axial‐aligned and percolated network rather than by NW loading.

### 2.3 Material‐Encoded Signal Decoupling via Alignment‐Dependent Stress Transfer

**FIGURE 4:**
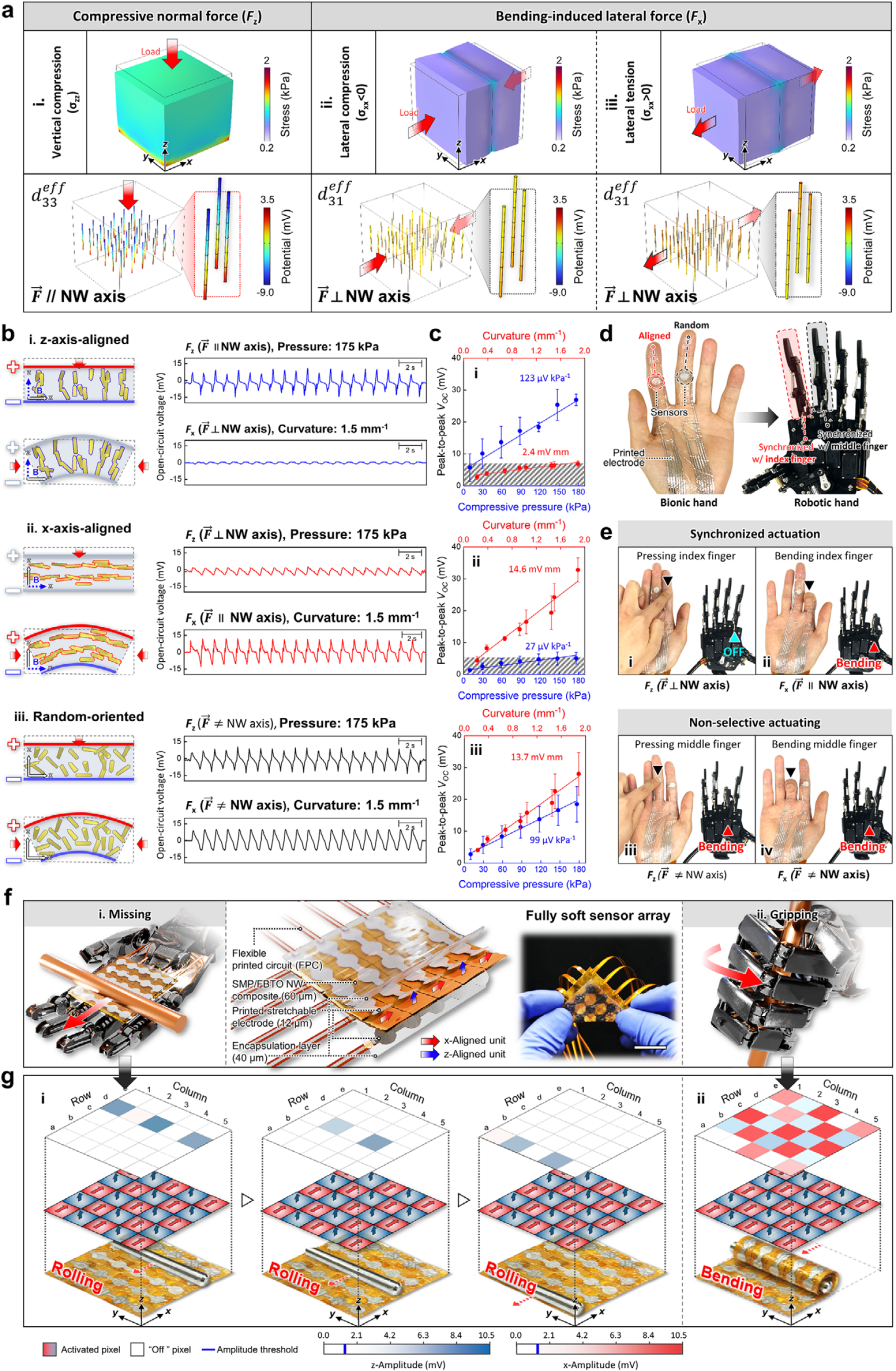
Mechanosensing mechanism and selective signal decoupling capabilities. (a) FEA simulating stress distributions (top) and piezoelectric potential generation (bottom). The models compare scenarios where the external load (*F*) is applied parallel to the NW axis (i, $d_{33}^{eff}$ mode) versus perpendicular to the NW axis (ii–iii, $d_{31}^{eff}$ mode). (b,c) Mode‐selective sensing performance. (b) Open‐circuit voltage (*V*_oc_) signals and (c) sensitivity plots for sensors with (i) z‐axis‐aligned, (ii) x‐axis‐aligned, and (iii) random NW orientations. Scale bar: 2 s. (d,e) Wearable interface for robotic hand control. (d) Photograph of skin‐conformal sensors on a human hand. An x‐aligned NW sensor (index finger) and a randomly distributed NW sensor (middle finger) are positioned. (e) Demonstration of signal decoupling: (i, ii) the aligned NW sensor enables precise bending‐only actuation by ignoring touching artifacts, whereas (iii, iv) the randomly distributed NW sensor triggers actuation non‐selectively. (f,g) Spatiotemporal discrimination of mechanical stimuli. (f) Schematic and photograph of a 5 × 5 matrix sensor array with alternating x‐ and z‐axis‐aligned pixels. Scale bar: 2 cm. Scenario depicts (i) object rolling with serial compressing and (ii) gripping with sequential bending. (g) *V*_OC_ amplitude mapping corresponding to the scenarios in f) with distinguishing between (i) the trajectory of a rolling cylinder and (ii) global deformation signals induced by bending.

To understand the physical mechanism underlying force‐mode selectivity in magneto‐piezoelectric composites and clarify how NW alignment affects stress transfer, finite element analysis (FEA) was performed to correlate the microstructural alignment with external stress propagation. In the thin‐film composite geometry (15 mm × 15 mm × 200 µm) defined herein, mechanical deformation is dominated by stress components aligned with the principal loading axis, whereas transverse stresses arising from lateral strain remain negligible [40]. Accordingly, the FEA focused on the efficiency of axial stress transfer as a function of NW orientation. Guided by the distinct alignment and hierarchical assembly states of FBTO NWs observed experimentally (Figure 3c,d), four representative orientation models were defined: (i) random, (ii) z‐axis‐aligned, (iii) z‐axis‐aligned/assembled into chains, and (iv) z‐axis‐aligned/assembled into bundles (Figure S14). To investigate their mechanical responses, two physically relevant deformation modes were considered with compressive normal force (*F*_z_) and bending‐induced lateral force (*F*_x_). While vertical compression gives a predominantly *σ*_zz_ stress field (Figure 4a‐i and Figure S14a), bending deformation induces a more complex stress profile. Following bending‐beam theory, this mode was modeled as opposing compressive stress (*σ*_xx_<0) and tensile stress (*σ*_xx_>0) induced relative to the neutral mechanical plane (Figure 4a‐ii, iii and Figure S14b). The resulting stress and piezoelectric potential mapping reveal a strong dependence of load transfer on NW geometry and microstructural arrangement. Under vertical compression, load transfer in the randomly oriented model is markedly hindered by discontinuous percolation pathways, leading to negligible stress localization and minimal potential generation (Figure S14a‐i). In contrast, z‐axis‐aligned NWs assembled into chain‐like architectures achieve the highest stress confinement and potential output (Figure 4a‐i and Figure S14a‐iii). This enhancement originates from the continuous head‐to‐tail connectivity along the z‐axis, which maximizes axial load transfer in a collinear direction with the NW alignment ($\vec{F}$ *∥*NW axis) and activates longitudinal effective piezoelectric mode ($d_{33}^{eff}$). Despite nominal z‐alignment, the bundled NW model showed reduced potential outputs, as lateral bundling disrupted the axial‐confined stress transmission and could further promote charge trapping and dielectric leakage (Figure S14a‐iv). When subjected to bending, all orientation models show lower stress concentration and reduced piezoelectric potentials. Notably, the z‐axis‐aligned chain models reveal a distinct shift toward a spatially inhomogeneous stress profile (Figure 4a‐ii, iii and Figure S14b). Since the applied load is perpendicular to the NW alignment ($\vec{F}$⊥NW axis), these models rely on the transverse effective piezoelectric mode ($d_{31}^{eff}$), where the $d_{31}^{eff}$‐driven potential is orders of magnitude lower than the $d_{33}^{eff}$‐driven response (Figure S15a). The contrast in potential generation demonstrates that efficient stress confinement and maximum energy conversion are achievable only when the loading direction is collinear with both the individual‐aligned axis and the hierarchical‐assembled microstructures of the NWs. The computational analysis is rigorously supported by experimental validation through tensile testing of the composites (Figure S16a–d). External mechanical loads applied parallel to the NW alignment direction result in strong stress confinement within the composite (red line; Figure S16b), while loads applied perpendicular to the NW alignment direction lead to significant stress attenuation (purple and blue lines; Figure S16b). The substantial directionality of stress transfer is fundamentally based on a finite load‐transfer zone (*L*_t_) across each NW, which maximizes efficiency only as the projected AR of the NW along the loading direction approaches its intrinsic high AR (see more details in Note S2 and Figure S16e,f). Therefore, the electromechanical transduction in magneto‐piezoelectric composites is critically governed by the synergy of both individual NW geometry with sufficiently high AR and their ensemble architecture.

Based on the axial‐dependent electromechanical transduction mechanisms of FBTO NWs, we developed force‐mode selective mechanosensors by strategically controlling the alignment and hierarchical assembly of the NWs within the thin‐film composites. Each sensor was configured as a thin‐film composite electrically poled in the out‐of‐plane direction (z‐axis) and sandwiched between top and bottom Ni electrodes, which directly generates an open‐circuit voltage (*V_OC_*) under mechanical stimuli without an external bias (see Methods for detail). To verify force‐mode selectivity, three distinct sensor configurations with (i) z‐axis‐aligned, (ii) x‐axis‐aligned, and (iii) randomly oriented NWs were subjected to vertical pressure and bending analogous to the FEA analysis. The z‐axis‐aligned NW sensor exhibited a dominant response to vertical pressure (175 kPa) with a peak‐to‐peak *V_OC_* of approximately 26.9 mV (Figure 4b‐i) and a high linear sensitivity of 123 µV kPa^−1^, whereas its sensitivity to bending remained negligible at 2.4 mV mm (Figure 4c‐i). The experimental results are consistent with the FEA predictions, where collinear loading along the NW axis ($\vec{F}$ *∥*NW axis) maximizes axial load transfer and thus activates the high‐efficiency longitudinal $d_{33}^{eff}$ mode. On the other hand, the x‐axis‐aligned NW sensor demonstrated a distinct reversal in force‐mode selectivity (Figure 4b‐ii), characterized by a high linear sensitivity to bending (14.6 mV mm) and a significantly suppressed response to vertical pressure (27 µV kPa^−1^; Figure 4c‐ii). The experimental output of the x‐axis‐aligned NW sensor under bending (*V_OC_* ∼24.2 mV) remarkably outweighed its response to vertical compression. The reversal in selectivity indicates that the total piezoelectric output is not just a function of intrinsic constants but is more critically governed by the stress transfer efficiency within the aligned and assembled NWs. In this configuration, bending‐induced lateral force is effectively concentrated along the longitudinal axis of the x‐axis‐aligned NWs, while vertical pressure acts perpendicular to the NW alignment ($\vec{F}$⊥NW axis), leading to inefficient load transfer and minimal potential generation. The selective load transfer along a programmed axis further demonstrates vector‐resolving capability even under a complex mechanical environment which multiple orthogonal stimuli are coexistent. Because the firmly embedded nanowires maintain their parallel alignment relative to the field‐programmed principal axes without undergoing rigid‐body rotation during macroscopic matrix deformation, the sensor output relies on the geometric vector between the loading axis and the internal anisotropy. Extensive multi‐axial testing demonstrated that under orthogonal in‐plane bending, the peak‐to‐peak *V_OC_* scaled proportionally with the applied curvature but exhibited a strong angular dependence. Thus, maximum sensitivity was achieved when the principal bending axis was parallel to the nanowire alignment (0°) and systematically diminished at perpendicular orientations (90°) (Figure S26). Moreover, when subjected to simultaneous multi‐axial loading using conformal 3D‐printed mechanical holders such as dynamic bending combined with dynamic out‐of‐plane pressure, or a 3D‐axial combination of simultaneous x‐ and y‐axis bending alongside z‐axis pressing, the orthogonally configured sensors reliably decoupled the superposed stimuli. Relying on this defined orientation of the embedded FBTO NWs, the aligned NW sensors not only suppress cross‐axis interference across an in‐plane bending mode ($\vec{F_{y}}$), but also distinguish superposed stimuli under the multi‐axial loading conditions ($\vec{F_{z}}$+$\vec{F_{x}}$, $\vec{F_{x}}$+$\vec{F_{z}}$, $\vec{\;F_{y}}$+$\vec{F_{z}}$, and even $\vec{F_{x}}$ + $\vec{F_{y}}$ + $\vec{F_{z}}$) without experiencing structural failure and noticeable signal distortion (up to 2.45 N), as shown in Figure S27. The results verify that the sensors operate reliably within a broad practical mechanical regime to accurately distinguish superposed stimuli even under highly complex multi‐axial deformation.

The necessity of such controlled anisotropy is further highlighted by the performance of randomly oriented NW sensors, which showed nearly isotropic electromechanical coupling with comparable sensitivities to both force modes (99 µV kPa^−1^ and 3.7 mV mm; Figure 4b‐iii,c‐iii). The absence of a defined alignment axis in the random model results in discontinuous percolation pathways and a failure to decouple different force vectors. Furthermore, the influence of non‐ideal intermediate alignment states on electromechanical responses was quantified to comprehensively evaluate the transduction mechanism (Figure S15b–g). The signal decoupling capability is severely compromised in the over‐densified state of FC(1500). Unlike the optimally aligned FC(500) composite that sharply separates bending stimuli from compressive pressure, the voltage outputs in the FC(1500) state overlap substantially, which causes a more isotropic electromechanical response and signal crosstalk. The dense and multidirectional branching limits directional load transfer. Thus, the bending sensitivity drops by 5.3‐fold compared to the FC(500) composite, while the pressure sensitivity slightly increases due to the lateral expansion of the active network. This quantitative degradation confirms that restricted and continuous collinear percolation pathways are absolute prerequisites for accurate force‐mode discrimination. Consequently, the results confirm that maximum energy conversion and high‐fidelity force‐mode differentiation are achieved through a geometric synergy, where the loading direction is collinear with both the individual NW axes and their hierarchical assembly. Alignment‐dependent stress transfer allows for the selective activation of electromechanical transduction modes directly at the material level. This physically embedded force‐mode discrimination enables directional mechanosensing without complex decoding and establishes a robust framework for real‐time, low‐power tactile interfaces.

The exceptional signal decoupling achieved through controlled NW orientation facilitates the application of these sensors in remote operation systems for advanced human‐machine interfaces (HMI), specifically demonstrated in prosthetic hand control (Figure 4d,e and Video S1). The sensing platform consists of ultrathin piezoelectric composite sensors (thickness: ∼6 µm; Figure S17a–c) printed onto a fully soft form factor with flexible printed circuit board (FPCB, thickness: ∼10 µm) patterned through liquid metal (Figures S17d,e and S18). The highly compliant sensing platform ensures intimate contact with finger joints while minimizing motion‐induced artifacts under severe deformation, which allows stable acquisition of real‐time piezoelectric voltage signals during the dynamic hand motions. The HMI system processes the *V_OC_* signal data generated by hand motions such as touching or bending fingers using a preliminary setup based on a digital multimeter and an Arduino Uno‐based control unit that converts the sensor outputs into modulated pulse signals (Figure S17f). The modulated signal is then transmitted to a servo motor for driving the prosthetic robot hand. Further, we established four main commands based on the threshold amplitude of *V_OC_* to control the bending angle of robot fingers (Figure S17g): (i) no response (0–2 mV), (ii) robot finger bending to 30° (2–4 mV), (iii) bending beyond 120° (> 4 mV), and (iv) return to the initial position (< 0 mV). Figure 4e highlights the critical importance of the tactile signal decoupling capability in HMI. A sensor incorporating x‐axis‐aligned NWs was attached onto the index finger joint, where bending sensitivity dominates, while the other with randomly oriented NWs was placed on the middle finger joint for comparison (Figure 4d). When the user bent the index finger, the x‐axis‐aligned NW sensor generated a high *V_OC_* (> 4 mV) signal that triggered a synchronized bending actuation of the corresponding robot finger exactly and promptly (Figure 4e‐ii). In contrast, when the index finger was subjected to a gentle touch or high vertical pressure, no corresponding action was triggered by the robot finger due to negligible *V_OC_* (< 2 mV) in the sensor with the x‐axis‐aligned NWs (Figure 4e‐i). The selective response of sensors enables such precise synchronization by decoupling joint bending from vertical pressure. By comparison, the randomly oriented NW sensor that is similar to conventional design of piezoelectric sensing devices demonstrated a critical limitation of non‐selective responses under both touching and bending [12, 14]. Thus, the synchronized robotic middle finger performed the bending action indiscriminately in response to any external mechanical perturbation, regardless of whether the human finger was bent or pressed (Figure 4e‐iii, iv). The absence of force‐mode discrimination led to unpredictable robot behavior and prevented reliable synchronization between human finger motions and robotic actuation.

The critical challenge in advanced robotics lies in achieving real‐time decision‐making capabilities in dynamic environments where visual recognition is unreliable. Therefore, a robot hand must discern whether its gripping action is successful or has failed due to object slippage or rolling (Figure 4f). Such robotic dexterity requires the real‐time decoupling of signals to identify whether the mechanical input originates from the external object movement or the intentional kinematic changes of the robot hand. Based on our strategies of decoupled tactile perception, we address this requirement by designing sensor arrays that enable directional and temporal signal distinction. For this purpose, we fabricated a fully soft sensor array composed of alternating x‐ and z‐axis‐aligned NW sensing units arranged in a 5 × 5 pixel checkerboard configuration (Figure 4g and Figures S18 and S19a,b; see Methods in detail). The sensor array verified the capability of real‐time tracking and force‐mode decoupling under two distinct, manually applied actions (Figure S19c–f). The first scenario simulated a gripping failure that was characterized by the object slipping. A metallic cylinder (diameter: 10 mm, weight: ∼70 g) was rolled sequentially across the entire sensor arrays, which induce a dominant z‐axis normal force (∼21 N) component under shear stress. When the cylinder was rolled over the checkerboard‐arranged sensor arrays, the z‐axis‐aligned NW sensing units were selectively and sequentially activated with showing distinct *V_OC_* amplitudes, while the x‐axis‐aligned sensing units remained nearly unresponsive (Figure 4g‐i). Selective and sequential activation validates the origin of signal as a rolling event of an object across the flat sensor arrays, while simultaneously allowing the robot to monitor the object's trajectory and kinematic parameters in real‐time. The resulting immediate feedback is crucial for the robot to recognize a gripping failure event and initiate a corrective action. In the second scenario, we simulated a successful grip. The sensor arrays on a robot hand were folded over the same cylinder, which conforms to the cylinder curvature. In this situation, the x‐axis‐aligned NW sensing units were activated sequentially across the entire sensor arrays with reporting high *V_OC_* amplitudes (Figure 4g‐ii). These high‐amplitude signals arise from the in‐plane strain and associated bending component which would be equivalent to the palm folding during a gripping motion. Concurrently, a subtle yet distinct activation was observed in the z‐axis‐aligned NW sensing units within the contact area, corresponding to vertical pressure exerted as the cylinder was fully gripped. Such decoupled bimodal sensing enables the robot to verify gripping success and adjust its bending actuation according to the characterized object size. In contrast, a control sensor array consisting of all‐randomly‐oriented NW sensing units exhibited a similar range of *V_OC_* amplitudes for both touching (*V_OC_*: 4–10 mV; Figure S19c) and bending (*V_OC_*: 4–12 mV; Figure S19e). Such non‐selective responsiveness lacks the required specificity to identify the event type without substantial post‐processing, thereby hindering real‐time robotic control. In contrast, our checkerboard‐arranged sensor array based on the orthogonal sensitivity of x‐ and z‐axis‐aligned NW sensing units enables distinct, directional, and temporal sensing. The spatio‐temporally resolved detection provides a practical framework for autonomous decision‐making for a robot to instantly differentiate between a successful grip and a missing object, while simultaneously assessing object diameter even under visual occlusion.

### 2.4 Perception‐Embodied Monolithic Soft Robots for In‐Operando Feedback

**FIGURE 5:**
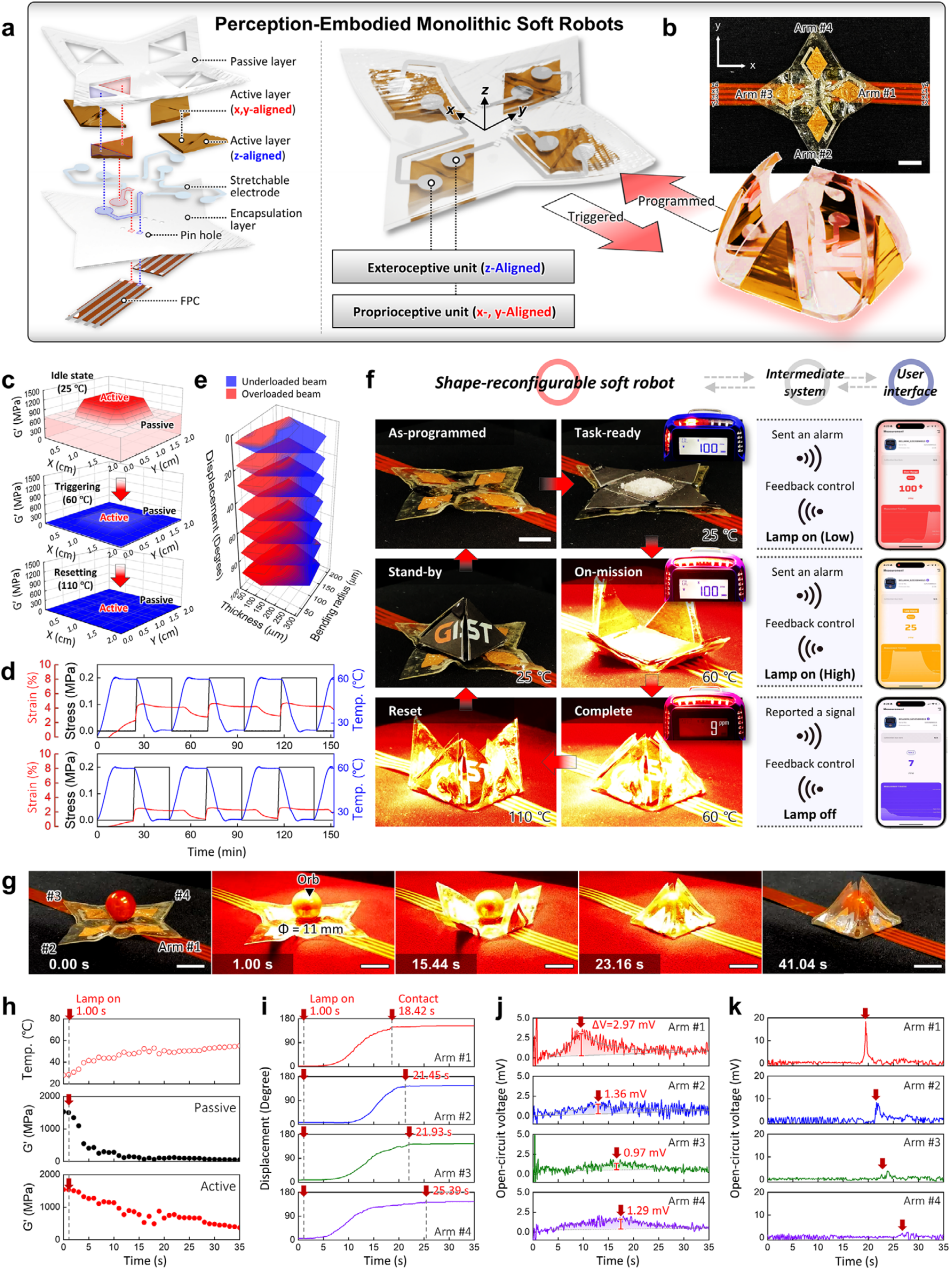
Perception‐embodied monolithic soft robots. (a) Schematic illustrating the monolithic robot design consisting of passive and active layers. The active layers feature orthogonal NW alignments (x/y‐axis‐aligned NW units for proprioception, z‐axis‐aligned NW units for exteroception) to decouple sensing modalities. (b) Photographs of the fabricated robot with shape‐reconfigurable actuations. Scale bar: 1 cm. (c) Spatiotemporal mapping of storage modulus (G') transitions during thermal triggering (60°C) and resetting (110°C). (d) Cyclic dynamic mechanical analysis (DMA) comparing the shape‐memory performance of pure SMP (top) and the 10 wt% FBTO composite (bottom). (e) Design phase diagram correlating geometric parameters (thickness, bending radius) with actuation angle, defining the payload capacity. (f) Operational workflow for hazardous contaminant isolation by assembling a packaging box. (g) Time‐lapse sequence of the robot autonomously grasping a target object (Φ = 11 mm). Scale bar: 1 cm. (h–k) Real‐time electromechanical feedback: temporal profiles of (h) temperature‐modulus and (i) actuation angle during assembly. Corresponding *V*_oc_ signals clearly distinguish between (j) proprioceptive bending deformation and (k) exteroceptive contact events.

The development of fully autonomous robotic systems is fundamentally dependent on continuous and high‐fidelity sensor feedback to enable closed loop control without human intervention. However, the extreme mechanical compliance and high degree‐of‐freedom (DoF) of soft robots pose a significant bottleneck, because the simultaneous superposition of diverse stress modes such as compression and bending induces signal crosstalk that makes it difficult to discriminate individual force vectors. Conventional strategies relying on the integration of discrete or rigid sensors conflict with the principles of soft robotics [8, 9, 41, 42, 43]. The additional mechanical payloads degrade compliance and agility, while the severe modulus mismatch between stiff sensing elements and soft bodies inevitably leads to interfacial delamination and mechanical failure. Furthermore, the necessity of integrating multiple sensor modules to resolve multimodal forces increases system complexity and mass, which ultimately restricts the robot's intrinsic deformation freedom. To overcome the aforementioned barriers, we propose a monolithic soft robotic system with embodied mechanosensing inspired by biological mechanoreceptors that discern complex forces through orientation‐specific responses [5]. By strategically embedding axial‐aligned FBTO NWs within a SMP matrix, we realized synthetic mechanoreceptors capable of intrinsic force‐mode decoupling. The seamless architecture, embedded with proprioceptive units (x‐ or y‐axis‐aligned NWs) at the hinge zones and exteroceptive units (z‐axis‐aligned NWs) at the arm centers, enables the selective encoding of the force vectors to distinguish between assembly actuation and contact with targets without cross‐axis interference (Figure 5a,b). Passive regions of neat SMP spatially isolate the active sensing zones to prevent signal crosstalk without sacrificing mechanical continuity. All sensing units are electrically interconnected using patterned liquid‐metal conductors that allow the robotic systems to acquire signals in real time while maintaining the compliance required for high‐DoF motion (see Methods and Figure S20 for more details).

Importantly, the monolithic soft robot maintains versatile shape‐morphing and reconfigurable actuation despite the embedded FBTO NWs. The thermomechanical actuation of the monolithic soft robot is governed by the entropy spring effect of the polymer chains, which allows the active and passive parts to restore their programmed shapes upon heating above the glass transition temperature (T_g_), as shown in Figure 5c,d. To investigate the influence of embedded FBTO NWs on the shape‐morphing and memory performance, we analyzed storage moduli (G‘) and loss tangent (tan δ) of each composite segment across various FBTO NW contents. The dynamic mechanical analysis (DMA) represents two distinct modulus drop regions near 50°C and 150°C (Figure S21c), corresponding to the T_g_ of the soft domains and the melting temperature (T_2_) of the hard domains, respectively. The identified transition points align with differential scanning calorimetric (DSC) thermograms that corroborate the dual‐phase morphology in the SMP matrix (Figure S21a). Additionally, the loss tangent and DSC curves demonstrate that the T_g_ remains almost stable even as the FBTO NW content increases up to 40 wt% (Figure S21b). In contrast, T_2_ shifts rapidly from 149°C to 157°C with minimal FBTO NW loading, indicating a long‐ranged ordering of the hard segments. The imbalanced reinforcement suggests a selective interfacial interaction where the FBTO NW surfaces favor the hard segments of the SMP, while leaving the soft segments largely unaffected [44]. Since the shape‐reconfigurable actuation is predominantly driven by the mechanical stretching and relaxation of the mobile soft segments, the FBTO NWs do not obstruct the underlying entropy spring mechanism. Therefore, the embedded FBTO NWs exert a minimal impact on the shape triggering and fixing temperatures that preserve the shape‐reconfigurable actuation performance regardless of the FBTO NW loading (Figures S21d, S22, and S24d). Notably, the programmed spatial alignment of the FBTO NWs is maintained during and after repeated thermal transitions above T_g_. Cross‐sectional OM images confirmed no spatial disorientation of the nanowires after 10 repeated thermo‐mechanical actuation cycles (Figure S28a). As previously verified, the selective interfacial interaction enables the crystalline hard domains to act as rigid physical anchors. These anchors firmly secure the nanowires and preserve their spatial alignment, even when the amorphous soft segments undergo a significant modulus drop during thermal actuation. To further evaluate the resistance to interfacial delamination driven by the significant modulus mismatch between the rigid ceramic NWs and the soft SMP matrix, the composites were subjected to an accelerated large‐strain bending fatigue test (100 cycles at a highly constrained radius of 55 µm and 60°C, applying a 20‐fold accelerated strain rate). Despite severe mechanical hysteresis, the composites exhibited no macroscopic mechanical failure. Post‐fatigue cross‐ sectional SEM confirmed that the nanowires remained stably embedded and maintained their programmed initial alignment (Figure S29). The observed microstructural damage, such as minor void and crack formations, was distributed randomly throughout the matrix volume rather than being localized at the nanowire‐matrix interfaces. This indicates that mechanical fatigue is governed by the inherent creep resistance of the polymer matrix, and the robust interfaces successfully facilitate stress transfer without localized interfacial debonding. Consistent with this structural integrity, isolated monolithic sensor‐actuator units demonstrated exceptional long‐term cyclic reliability, which maintains stable and reproducible peak‐to‐valley *V_OC_* amplitudes and macroscopic angular displacements over continuous shape‐morphing cycles without remarkable baseline degradation (Figure S28d–f). Robust mechanical stretching and thermal relaxation of the monolithic soft robot body enable the execution of diverse operation modes, including box assembly, origami folding, and object encapsulation (Figure S23). Detailed morphing curvatures were programmed based on elastic beam theory to transition from 2D planar to 3D square pyramidal shapes (Note S3 and Figure S24). In brief, increasing composite film thickness while minimizing the programming bending radius amplifies the bending moment, which allows the system to lift heavier payloads and achieve greater displacement upon recovery. The programmed parameters ensure the adaptability required for the diverse operational modes (Figure 5e and Figure S23).

Building upon the theoretical and experimental results of elastic beam mechanics for magneto‐piezoelectric composites (Note S3 and Figure S28), we designed two distinct robotic actuation modes to highlight the kinematic‐sensory fidelity of our perception‐embodied soft robot: (i) simultaneous folding actuation for box assembly with low bending moments (Figure 5f and Figure S25) and (ii) sequential folding actuation for object enclosure with large bending moments (Figure 5g). First, we demonstrated the autonomous packaging and isolation of toxic contaminants ((NH_4_)_2_CO_3_) in hazardous environments (Figure 5f). By programming all robotic arms with high bending radii (∼440 µm, see Methods), we induced uniform, low bending moments upon thermal triggering. This design enabled the arms to fold (125°) simultaneously, which successfully lifted the packaging box (∼200 mg per one plane) and encapsulated the toxic contaminants without the misalignment issues of each arm often caused by torque imbalances. During the simultaneous folding sequence, the embedded sensing units accurately captured the shape‐morphing actuation and reported simultaneous signal onsets. Consistent with the low mechanical stress generated by the high‐bending radii configuration, the signals exhibited low and comparable amplitudes of approximately 0.55 mV (Figure S25). The output profile confirms that only proprioceptive mechanisms were active in the absence of external contact. To validate the containment, an external NH_3_ gas sensing module was used to monitor the local environment, with the concentration data displayed in real‐time via a graphical user interface on a personal smartphone. Following the box assembly, the detected NH_3_ concentration dropped rapidly from 100 ppm to a saturated value of 9 ppm, indicating that the arms were firmly interlocked and effectively enveloped the inner contaminant. Once the environmental safety was confirmed—specifically, when the NH_3_ level fell below the short‐term exposure limit of approximately 35 ppm—the system demonstrated its reusability through shape‐reconfigurable actuation. By heating the robot to 110°C above the melting transition of short‐ordered hard domains (T_1_; Figure S21a), the robotic body underwent a significant modulus drop (Figure 5c and Figure S21c) and further turned to a state where the polymeric matrix can no longer retain its freestanding structural integrity. The thermally induced phase transition initializes the programmed shape, a crucial step that prepares the system for successive reprogramming toward future objectives (Figure 5f). The absence of reverse‐polarity signals during thermal recovery is attributed to the increased loss modulus of the heated matrix, which diminishes load transfer efficiency and uncouples mechanical stress from the embedded FBTO NWs. Simulating a hazardous scenario inaccessible to human intervention confirms that the monolithic soft robot can execute an encapsulating sequence for the chemical toxicant, relying solely on embedded mechanosensory feedback without visual assistance.

To further explore the robotic system's capacity for resolving complex temporal interactions, we programmed the robot with graded low bending radii of 55, 110, 165, and 220 µm (Figure 5g; see Methods for details). Unlike the previous uniform high‐radius design, the graded curvature induces significant differences in bending moment across the arms. Therefore, even under a global thermal stimulus, the moment differentials dictate a stepwise sequential folding actuation (Figure 5h,i), captured with high fidelity by the embedded sensing units. Subsequent analysis of the *V_OC_* signals revealed that both signal onsets and peak amplitudes were intrinsically coupled to the specific bending radius of each arm. Specifically, the low‐radii configuration generated higher signal amplitudes due to concentrated mechanical stress, while simultaneously exhibiting a time‐delayed acquisition pattern that perfectly mirrored the sequential actuation (Figure 5j). It is noteworthy that the apparent discrepancy between the free‐space programmed arm angles and the proprioceptive signals during soft robotic grasping (Figure 5i,j) arises from the physical obstruction by the central orb. The target object physically restricts the completion of bending actuation, converting the excess bending moments into contact forces applied to the exteroceptive units. To isolate the intrinsic proprioceptive mechanics without structural artifacts, isolated monolithic sensor‐actuator units were tested, which demonstrates a highly reliable one‐to‐one mapping between the macroscopic displacement and *V_OC_* amplitudes over continuous cycles (Figure S28b–f). Furthermore, to overcome environmental electromagnetic interference (EMI) inherent to unshielded compliant liquid‐metal flexible printed circuits, an advanced non‐referenced single‐ended (NRSE) signal acquisition circuit incorporating a voltage reference buffer follower was developed (Scheme S1 in Note S3). This architecture successfully attenuated the baseline noise offset from ∼2.0 to ∼0.2 mV. Because viscoelastic stress relaxation at elevated temperatures above T_g_ and slower strain rates dampens the raw voltage magnitude during soft robotic actuation compared to benchmark sensors, the integrated system uses an event‐driven peak‐detection methodology. By evaluating the time period and amplitude of characteristic signal peaks processed through the NRSE circuit, the control system accurately monitors the precise onsets and endings of the sequential folding events. Furthermore, as the sequential folding facilitated successive physical contact with the enclosed object, the exteroceptive units resolved these events as discrete secondary peaks, temporally separated and clearly distinguishable from the primary actuation signatures (Figure 5k). The distinct signal amplitudes generated upon contact directly correlate with the localized normal forces. Because the structural anisotropy keeps the exteroceptive sensors unresponsive to collinear bending strains, complete force‐mode isolation is achieved. To quantitatively map the raw piezoelectric signals to the physical contact forces, we applied an analytical calibration model combining the recovery torques of the shape‐memory polymer with Hertzian sphere‐on‐flat contact mechanics (Note S4). By translating the excess recovery torques restricted by the orb into dynamically varying normal contact areas, the raw *V_OC_* peak magnitudes that calibrated with the linear compressive sensitivity of the z‐axis‐aligned sensor (123 µV kPa^−1^) were converted into estimated localized contact forces. The calibrated forces ranged from 0.44 to 3.46 mN (Figure S30), demonstrating a clear dependency on the programmed bending radii of the individual arms and thoroughly validating the exteroceptive fidelity of the system. Meanwhile, the graded bending moments resulted in a cascade of *V_OC_* signals that consecutively diminished in accordance with the programmed radii. This behavior, consistent with our theoretical expectations, confirms that the monolithic soft robotic system can effectively decouple and resolve complex and multi‐stage physical interactions in real time. Overall, in‐body force‐mode discrimination simplifies the sensory‐motor control loop and further enables the development of energy‐efficient, self‐supervising soft machines capable of sophisticated environmental interaction and autonomous decision‐making.

## 3 Conclusion

In this study, we have established a material‐level strategy for field‐programmed anisotropy that effectively upscales the polar directionality of piezoelectric nanowires to macroscopic soft composites. Guided by a torque‐balance framework of magneto‐elastic rotational dynamics, the deterministic spatial alignment of magneto‐piezoelectric nanowires overcomes the fundamental limitations of random percolation to produce axially aligned hierarchical networks that maximize electromechanical coupling efficiency. These structurally anisotropic architectures govern the stress transfer mechanics by establishing continuous pathways for mechanical load concentration along the principal axes of the nanowires and strictly suppressing off‐axis interference. Ultimately, this magnetic‐field‐guided approach provides infinite degrees of freedom to deterministically program nanowire alignment across arbitrary 3D spatial axes. Such precisely controlled internal architectures empower the magneto‐piezoelectric composite with vector‐resolved mechanosensing capabilities. As a result, the composite intrinsically functions as a physical decision‐making filter that translates complex multidirectional stimuli into highly specific and direction‐dependent electrical outputs.

Conventional soft robotic systems typically achieve proprioception and exteroception by integrating discrete rigid sensor modules onto compliant bodies. The traditional integration strategy fundamentally degrades overall mechanical compliance, restricts kinematic degrees of freedom due to added mechanical payloads, and inevitably induces interfacial delamination. Furthermore, decoupling multi‐axial forces in these systems necessitates complex multi‐sensor array configurations and intensive computational post‐processing. Our perception‐embodied monolithic design effectively resolves these critical limitations by advancing the aforementioned material‐encoded capabilities to the system level. By directly embedding the active sensing materials within the shape‐memory polymer matrix, the seamless architecture completely resolves the interfacial modulus mismatch. More importantly, the field‐programmed spatial anisotropy inherently decouples distinct force vectors directly at the material level, effectively eliminating the need for complex multi‐sensor arrays and offloading signal discrimination tasks from external algorithms to intrinsic material properties. Consequently, this work establishes a foundational platform for material‐defined intelligence that facilitates future robotic systems to navigate environmental complexity through physical adaptation rather than computational load.

## 4 Methods

### 4.1 Materials

Titanium(IV) oxide (TiO_2_, anatase, 99.7%), barium hydroxide octahydrate (Ba(OH)_2_∙8H_2_O, ≥98%), barium titanate particle (BaTiO_3_, 99.5%), iron(II) chloride tetrahydrate (FeCl_2_∙4H_2_O, 98%), iron(III) chloride hexahydrate (FeCl_3_∙6H_2_O, ≥98%), ammonium hydroxide solution (NH_4_OH, 28.0–30.0% NH_3_), and poly(sodium 4‐styrenesulfonate) (M_w_ = 70000) were purchased from Sigma‐Aldrich. Sodium hydroxide (NaOH, >98.0%), formic acid, and polyvinylpyrrolidone (PVP, M_w_ = 40000) were obtained from Tokyo Chemical Industry. Dimethyl sulfoxide (DMSO, 99.9%), and hydrochloric acid (HCl, 35.0–37.0%) were provided by DUKSAN. A shape memory polymer (polyether‐polyol‐based polyurethane, SMP) was obtained from SMP Technologies. A filament type of SMP was cut into small pieces for further use. A thermoplastic polyurethane (TPU) was purchased from BASF. To form gallium indium eutectic alloy (EGaIn), an In ingot was cut into tiny pieces and mixed with Ga (both were purchased from RUICUI‐Changsha Rich Nonferrosou Metals) at 120°C for 2 h. EGaIn was stored in an aqueous solution of NaOH (0.5 M) for further use.

### 4.2 Synthesis and Magnetic Functionalization of BaTiO_3_ Nanowires

High‐aspect‐ratio BaTiO_3_ nanowires (BTO NWs) were synthesized through two‐step hydrothermal process. Initially, TiO_2_ powder (1.5 g) was dispersed in 10 M NaOH (60 ml) and hydrothermally synthesized at 180 °C for 48 h to form Na_2_Ti_3_O_7_ NWs. Na_2_Ti_3_O_7_ NWs were washed with H_2_O to reach a neutralized pH and immersed in 0.2 M HCl for 24 h to form H_2_Ti_3_O_7_ NWs via ion exchange reaction. After neutralization and drying, the H_2_Ti_3_O_7_ NWs (0.14 g) were mixed with Ba(OH)_2_·8H_2_O (1.025 g, corresponding to Ba:Ti molar ratio of 2:1) in H_2_O (60 ml). The mixture was sonicated for 3 h and heated at 200 °C for 1.5 h in a Teflon‐lined autoclave. The final BTO NWs were purified using 0.2 M HCl, H_2_O, and EtOH, followed by vacuum drying at 65 °C.

Magnetic functionalization was achieved by co‐precipitating Fe_3_O_4_ nanoparticles (NPs) onto the BTO NW surfaces. BTO NWs (0.6 g) were treated with PVP (0.1836 g) in H_2_O (15 mL) at 70 °C for 2 h. The suspension was cooled to 55 °C, and FeCl_3_·6H_2_O and FeCl_2_·4H_2_O were added in a 2:1 molar ratio. After stirring for 0.5 h, NH_4_OH was added dropwise (16 mL h^−1^) until the pH reached 11. The reaction proceeded for an additional 0.5 h. The resulting Fe_3_O_4_ NPs‐decorated BTO NWs (FBTO NWs) were collected by magnetic separation and washed with EtOH and vacuum‐dried at 65 °C.

### 4.3 Fabrication and Electromechanical Characterization of Magneto‐Piezoelectric Composites

Magneto‐piezoelectric composite films composed of SMP as a matrix and FBTO NWs as nanofillers were fabricated with solution casting technique. FBTO NWs (1 – 40 wt% relative to total mass) were added to a 10 wt% DMSO solution of SMP and mixed for 1 h with a planetary mixer (400‐DI, Hanil Global Technology). The mixture was cast onto a glass substrate within a rectangular polydimethylsiloxane (PDMS) mold (1.5 × 1.5 cm^2^). To achieve magnetic alignment of the NWs, a magnetic field was applied at 80 °C for 4.5 h with the aid of an electromagnetic coil, followed by drying in a convection oven for 19.5 h. Otherwise, for randomly oriented NW composites, the films were dried for 24 h without a magnetic field. All composites were subsequently vacuum‐dried at 80 °C for 24 h to remove residual DMSO, ultimately obtaining homogeneous light‐brown films (∼200 µm thick). For electrical contact, Ni fabrics (1 × 1 cm^2^) electrodes were attached onto the composite films via mild hot‐pressing (∼2.2 N) at 120°C for 5 min.

After fabrication, a stepwise DC field ramping was applied to pole the composites while preventing electrical breakdown. The composite films sandwiched by Cu plates (4 × 4 × 0.05 cm^3^) were immersed in an oil bath. To block the short circuits from fringing fields, smaller auxiliary Cu plates (1 × 1 × 0.05 cm^3^) were placed between the main Cu plates and Ni electrodes. The electric field was gradually increased from 2.5 to 15 kV mm^−1^ stepwise by alternating 15‐min DC fields applications with 10‐min resting at zero field. The maximum field was then kept for 3 h (for randomly oriented and x‐axis‐aligned NW composites) or 2 h (z‐axis‐aligned NW composites). After the poling, the composite films were washed with n‐hexane.

The electromechanical output performance of composite films as sensors was evaluated upon repeated compression or bending cycles (frequency: ∼1 Hz, JIPT, JUNIL TECH). The poled composite films were mounted on linear motor and their open‐circuit voltage (*V_OC_*) was recorded using a digital multimeter (DMM7510, Keithley) and tri‐axial cable (TRX, Keithley). In particular, we utilized the centermost conductors in a pair of the TRX cables to maximize shielding efficacy. To eliminate artifacts from triboelectrification, the gap between the sensor and compression head was strictly maintained below 1 µm, or composite films were encapsulated with Kapton tape for bending tests. A digital force gauge (AI‐35T, CASKOREA) and image‐based curvature analysis were used to quantify the applied compressive load and bending radius, respectively. With this measurement protocol, we conducted the electric measurement which compares unpoled and electrically poled composite sensors to clarify piezoelectric origin of the acquired signals. Briefly, the peak‐to‐peak *V*_oc_ magnitudes are enhanced by approximately 12.6 times following the poling process (Figure S31a,b), confirming that our measurement method effectively rejects external signal contamination by possible contributions from triboelectrification and undesirable interfacial friction.

Potential pyroelectric contributions were also excluded. Continuous and steady infrared heating resulted in a negligible rate of temperature change (d*T*/d*t* ≈ 0) during sequential folding. Furthermore, unprogrammed monolithic units exhibited no discernible voltage spikes under various controlled thermal variations (5 – 35 °C) (Figure S31c–f), and the *V*_oc_ peak amplitudes of programmed samples were entirely dependent on the programmed mechanical curvature (Figure S32). To reduce environmental EMI captured by unshielded compliant circuits, the advanced NRSE circuit equipped with passive filters and a voltage reference buffer was used (Scheme S1 in Note S3).

For the evaluation of signal decoupling capability under complex mechanical environments, we fabricated the custom mechanical holders featuring specifically designed convex and concave structures using a 3D printing method (P1P, Bambu Lab). The custom holders can conformally guide each sensor toward the intended complex bending and pressing stimuli without arbitrary structural distortion. Through these setup, various interferential forces were examined across orthogonal directions simultaneously to evaluate the sensing capabilities to distinguish outputs under complex multiaxial deformation.

### 4.4 Fabrication and Integration of Ultrathin Composites with Flexible Printed Circuit Boards

The fabrication process began with the preparation of 6‐µm‐thick composite films using a sacrificial layer method (Figure S17a–c). A glass substrate (5 × 5 cm^2^) was thoroughly cleaned with H_2_O, EtOH, and IPA successively by ultrasonication. A sacrificial layer of 20 wt% poly(sodium 4‐styrenesulfonate) (M_w_ = 70000) was spin‐coated onto the glass at 2000 rpm for 30 s and completely dried at 100 °C for 3 h. Afterward, 0.5 mL of SMP/FBTO NW/DMSO mixture (SMP:DMSO = 1:9 in w:w, FBTO NW:SMP = 1:9 in w:w) was spin‐coated onto the sacrificial layer, then dried at 100 °C for 24 h, and further vacuum‐dried at 80 °C for 24 h. The resulting multi‐stacks were immersed in H_2_O bath and gently shaken to dissolve the sacrificial layer (Figure s17c). The delaminated thin film was cut into 1.5 × 1.5 cm^2^, sandwiched by Cu plates after drying residual H_2_O, and poled as described above. Surface profiling (DektakXT, BRUKER) confirmed an average thickness of ∼209 nm for the sacrificial layers and ∼6.45 µm for the composite films (Figure S17b).

Following the film preparation, Ag‐Ga‐In‐based flexible printed circuit boards (FPCBs) were fabricated for the on‐skin sensor array. An electrode pattern (line width = 1.3 mm) was cut from a vinyl sheet using a cutting plotter (KI‐375A, VEVOR) and transferred onto polyurethane‐based waterslide decal paper as a substrate. A mixture of Ag epoxy adhesive (1:1 base/curing agent ratio, diluted with IPA, MG Chemicals) was printed onto the masked sheet, and the pattern was cured at 65 °C for 3 h after solvent removal. EGaIn was subsequently printed onto the Ag traces and incubated for 3 h at ambient conditions. After removing the mask, the prepared SMP composite films were affixed to the FPCB using H_2_O, while flexible printed cable (FPC) was attached using Ag epoxy and anisotropic conductive film (ACF). The electrode width was matched to that of the FPC (1.27 mm). Finally, the device was encapsulated with a sprayed acrylic insulating agent (R ∼10^14^ ohms, clearcoat) and dried for 0.5 h (Figure S17d). The resulting sensors were hydro‐transferred onto human skin by eliminating a paper supporter using H_2_O, which exhibited a highly flexible and deformable on‐skin sensor with a total thickness of ∼10 µm (Figure S17e).

### 4.5 Fabrication of Flexible Sensor Arrays and Implementation of Remote Prosthetic Control

To fabricate an ultrastretchable electrode suitable for flexible electronics, a liquid‐metal‐particle‐based conductive ink was prepared using poly(sodium 4‐styrenesulfonate) (PSS, Mw = 70000) and a weak acid. Specifically, 3.5 mM of formic acid (FA) was used not only as a scavenging agent to chemically sinter the oxide layer but also to ensure that a thin oxide layer remains the topmost surface due to its volatile nature (vapor pressure ∼ 45 mmHg at 20 °C). This method improves mechanical durability compared to pristine liquid metal (see Figure S18i–k) while remaining a conductive pathway underneath (Figure S18f). The ink was synthesized by dissolving PSS and FA in 1.8 g of H_2_O, adding 1.25 g of EGaIn, and sonicating the mixture for 0.5 h at 25 kHz (80% intensity) in an ice bath. The proposed interface structure of a liquid metal particle was illustrated in Figure S18a.

To evaluate the electric conductivity, four individually synthesized conductive inks were patterned and dried onto a glass substrate with a square shape (1.5 × 1.5 cm^2^). Subsequently, a sheet resistance was measured through four‐point probe method by utilizing a probe station (MODEL 8000, MS TECH) equipped with an optical microscope (FOK‐100W, Fiber Optic Korea) and a sourcemeter (SMU2636b, Keithley), where the averaged values show the proper conductivity when compared with EGaIn and liquid metal particle without adding PSS and FA (Figure S18b). To examine stability under mechanical and thermal stress, the ink was cast onto VHB tape. Resistance changes were monitored under tensile strain or heating cycles, which confirms its suitability for stretchable electronics (Figure S18e–g) and high‐temperature processing/operation cycles of soft robots (Figure S18h).

The conductive ink was subsequently utilized in the fabrication of two‐dimensional tactile sensor arrays. Twenty‐five composite films (each size: 1 × 1 × 0.006 cm^3^) with varying alignment states (random, x‐axis‐aligned, and z‐axis‐aligned) were arranged on a glass substrate and thermally welded at 175 °C. All the welded interfaces were thoroughly examined by using an optical microscope to avoid the leakage of a conductive ink, followed by cleaning with IPA. The conductive ink was then patterned in a matrix configuration on both the top and bottom surfaces. The entire assembly was encapsulated with two neat SMP films (40‐µm‐thick) with five pin holes (1.2 × 1.2 mm^2^) for electric connection to the poling apparatus or measurement systems (DMM7510/NI‐USB‐6211) via FPCs.

Finally, to demonstrate the practical application of the sensor array, a remote prosthetic hand control system was developed. Real‐time analog voltage signals from the composite sensors were acquired using a DMM7510 and converted to a digital signal (levels 0–3) via Python‐based algorithm (Figure S17f,g). Python was also used to manage the graphical user interfaces (GUI) and communicate with DMM7510 to an Arduino Uno, which processed the digital signals to modulate servo motors through pulse‐width modulation (PWM) with a duty cycle of 1.2 ms and control transistors (NPN‐type BJT), thereby enabling precise operation of the prosthetic hand (Figure S17g and Video S1).

### 4.6 Development of Monolithic Soft Robots and Operation Protocols

A monolithic soft robot was constructed by successively stacking a neat SMP passive layer, eight active composite films (x‐ and z‐axis‐aligned NWs), conductive ink, and a neat SMP encapsulation layer. The neat layer and composite films were cut and thermally welded at 175 °C to ensure uniform interfaces (Figure S20). Conductive ink was patterned on the top surface and then encapsulated with an SMP film featuring eight pin holes (1.2 × 1.2 mm^2^) for FPC connections to facilitate poling and signal measurement. As a fundamental test for proprioceptive signaling during thermo‐mechanical actuation, the monolithic sensor‐actuator units with x‐axis‐aligned NWs (1.5 mm × 1.5 mm × 200 µm) were programmed with the curvature of 55, 110, 165, and 220 µm, and *V*_OC_ signals were monitored under 60 °C by a heat lamp (0.8 W cm^−2^, 75% intensity).

The robot was programmed for various operation modes including origami folding, object encapsulation, and box assembly. For shape reconfiguration, each arm part of planar robot body was folded at 60 °C, intercalated with Teflon spacers (55 µm) to prevent adhesion, and annealed at 120 °C for 5 min. A temporary planar shape was fixed by heating to 60 °C, straightening, and cooling, successively. Thus, shape recovery was triggered by a heat lamp (0.8 W cm^−2^, 75% intensity) with deformation angles monitored by Kinovea software based on the recorded videos and temperature profiles by using a Fluke thermal measurement system (Fluke 179 TRUE‐RMS MULTIMETER, FLUKE).

Representatively, contaminant isolation application scenarios were demonstrated. For isolation, a home‐made packaging box was fabricated using Mylar panels (50‐µm‐thick), and Ecoflex hinges (∼100‐µm‐thick) with magnetic studs (ferrite magnetic particles in chlorinated polyethylene, 5 × 2.5 mm^2^) affixed to the surface of Mylar to maintain the assembled state after completion of robotic guidance. To ensure the latching occurred exclusively through the intended robotic motion, the studs were diagonally magnetized (45°‐relative to the out‐of‐plane direction, 15 kOe). A total weight of one arm was designed below 200 mg, ensuring an arm is within a range of payload capacity for a robotic motion (see Note S3). The programmed shape‐morphing robot enclosed ammonium carbonate ((NH_4_)_2_CO_3_), which served as a model hazardous contaminant, and the successful assembly was validated by observing a reduction in NH_3_ gas concentration from 100 to 9 ppm (BMI‐NH3, GDI Solution) and simultaneously monitoring piezoelectric signals (∼0.55 mV).

To evaluate the lifespan of the soft robots upon a prolonged operational cycle, we performed long‐term cyclic test of thermo‐mechanical actuation while observing morphological change, and monitoring *V*_OC_ signal variation as well as shape‐memory performance of the composite (Figures S28, S29, and S32 and Note S3). In brief, repeated actuation and reprogramming over 10 cycles were carried out under 60 °C by a heat lamp (0.8 W cm^−2^, 75% intensity) by acquiring *V*_OC_ response signals with the modified measurement circuits (see detailed information from Scheme S1 in Note S3). Subsequently, all the samples with the thermo‐mechanical hysteresis were fractured to obtain a cross sectional OM image by cryogenic cutting method. Separately, to evaluate and extend much longer cyclic period, an accelerated degradation test (ADT) was performed under severe large‐strain bending fatigue at a radius of 55 µm and an elevated temperature of 60 °C. The mechanical testing was extended over 1000 cycles to rigorously simulate the actual operating conditions of the soft robotic system. To introduce larger mechanical stress and accelerated potential structural damage at the composites, an approximately 20‐fold acceleration in the strain rate was applied.

### 4.7 Characterization

#### 4.7.1 Structural and Morphological Analysis

Morphological features were examined using high resolution field emission scanning electron microscope (Verios 5 UC, Thermo Fisher Scientific) or optical microscope (IX73, Olympus). Crystal structures, elemental evolution and distributions were analyzed via X‐ray diffractometer (SmartLab, Rigaku), X‐ray photoelectron spectra (NEXSA, Thermo Fisher Scientific), and transmission electron microscopy equipped with energy dispersive spectroscopy (Tecnai G2 F30 S‐Twin, FEI). To visualize the 3D alignment of FBTO NWs within the polymer matrix, 3D X‐ray tomography microscope system (Zeiss) was used, followed by orientation analysis using Geodict software. The layered imaging method initially provided ∼900 slices of sample images, and three‐dimensional models were reconstructed using Dragonfly 3D World software (ZEISS edition). Small‐angle X‐ray scattering measurements (Nanopix, Rigaku) were conducted at room temperature with a detector positioned at 468 mm from the sample. The accessible scattering vector (q) was ranged in 0.04‐4 nm^−1^, which can be calculated from q = $\frac{4\pi }{\lambda }$ sin(θ/2), where θ stands for a scattering angle.

#### 4.7.2 Surface Chemistry and Magnetic Properties

Surface chemistry evolution of nanomaterials was characterized by measuring zeta potential (Zetasizer Nano ZSP, Malvern Instruments). To separate specific surface effects, control samples (poly(pyrrolidone) (PVP)‐treated, acid/based‐exposed BTO, and FBTO NWs) were neutralized prior to analysis. Magnetic properties were evaluated using a vibrating sample magnetometer (7400 Series, Lake Shore Cryotronics) with a field sweep from −5 to 5 kOe. Anisotropy arising from Fe_3_O_4_ NPs arrangement was specifically assessed using coin‐shaped composites (Φ = 16 mm, t = 5 mm) under in‐plane azimuthal magnetic fields to eliminate bulk shape anisotropy effects.

#### 4.7.3 Electromechanical, Thermal, and Mechanical Testing

Local piezoelectric responses were mapped with a piezoresponse force microscopy (NX20, Park Systems) on Ta/Pt‐coated Si wafers. Ferroelectric polarization‐electric field (*P‐E*) hysteresis loops of the composite films were recorded by sweeping an external bias with a range of −3 to 3 kV, utilizing a ferroelectric tester (Precision Multiferroic Ferroelectric Tester and High Voltage Interface, Radiant Technologies). Mechanical tensile tests were performed using a universal testing machine (UNITEST M1, TEST ONE) on rectangular specimens (1 × 0.5 cm^2^) at a strain rate of 104 mm min^−1^ to simulate bending condition. Typical electric measurement for bending mode was conducted at 1 Hz of frequency and the strain rate were yielded by general expression of a bending strain model, $\varepsilon_{top}=\frac{t}{2r}$, where *ε* is bending strain (tension), *t* is thickness of specimens, and *r* is bending radius of curvature. Dynamic thermomechanical analysis (Discovery 850, TA Instrument) was conducted in both temperature ramping mode (−50 to 180 °C, strain to samples 0.1%) and controlled force mode (temperature cycles from 25 to 60 °C or 60 to 25 °C upon isostress condition of 0.2 MPa) to evaluate shape memory behaviors. For data reliability, isotherm steps (5 min) were also inserted to the temperature cycles.

## Funding

This work was supported by Grants RS‐2025‐25442536 and RS‐2024‐00407155.

## Conflicts of Interest

The authors declare no conflicts of interest.

## Supporting information




**Supporting File 1**: adma73967‐sup‐0001‐SuppMat.pdf.

**Supporting File 2**: adma73967‐sup‐0002‐VideoS1.mp4.

## Data Availability

The data that support the findings of this study are available from the corresponding author upon reasonable request.
