# Supplementary material for: Field‐Programmed Anisotropy in Magneto‐Piezoelectric Composites for Material‐Encoded Mechanoperception
Source: Adv Mater. 2026 Jul 18;38(48):e73967. doi: 10.1002/adma.73967 (PMC13508759; doi:10.1002/adma.73967)
Supplement: Supplementary file 1 [file ADMA-38-e73967-s002.pdf]

Supporting Information

Field-Programmed Anisotropy in Magneto-Piezoelectric Composites for Material-Encoded Mechanoperception

Yubin Kim[1], Yumin Kwon[1], Dabin Kim[2], Minsun Oh[1], Jin Young Jung[3], Dongyeong Gim[1], Kootak Hong[4], Jun Yeon Hwang[3], Sang-Woo Kim[2], Minjeong Ha[1,*]

[1]Department of Materials Science and Engineering, Gwangju Institute of Science and Technology, Gwangju, Republic of Korea
[2]Department of Materials Science and Engineering, Center for Human-oriented Triboelectric Energy Harvesting, Yonsei University, Seoul, Republic of Korea
[3]Institute of Advanced Composite Materials, Korea Institute of Science and Technology, Jeonbuk, Republic of Korea
[4]Department of Materials Science and Engineering, Chonnam National University, Gwangju, Republic of Korea

[*]Correspondence: Minjeong Ha (minjeongha@gist.ac.kr)

Table of Contents

Supplementary Notes 3
Supplementary Figures 15
Supplementary References 49

Supplementary Note 1: Theoretical framework and magnetomechanical alignment dynamics

General torque balance model

The axial alignment of FBTO NWs within a viscoelastic medium is governed by the interplay of competing torques. While previous models primarily attributed alignment to the balance between gravitational torque (τ_g) and anisotropic paramagnetic torque (τ_{pm}) [1], such frameworks are insufficient for describing behavior in non-Newtonian or solid-state regimes where matrix resistance is non-negligible. To rigorously predict the critical magnetic field strength (H_c) required for axial alignment, we established a comprehensive torque balance model incorporating the elastic restoring torque (τ_{el}). The condition for alignment is defined as the state where the magnetic driving force overcomes the combined resistance of gravity and matrix elasticity. We numerically estimated the minimum field strength satisfying the inequality by solving for the individual torque components derived below.

Component torque derivations

The gravitational torque (τ_g), which acts to rotate the FBTO NW away from the vertical axis, is defined as:

$$\tau_g = g\left(\frac{l}{2}+t\right)V_{NW}(\rho_{NW}-\rho_{med})\sin\psi \tag{1}$$

where g is the gravitational acceleration constant (9.8 m s^{-2}), l and V_{NW} are the averaged length and volume of FBTO NWs, t is the averaged diameter of the surface Fe_3O_4 NPs, and ρ_{NW} and ρ_{med} denote the densities of the NW and dispersing medium, respectively. ψ represents the angle relative to the gravitational vector. Although a vertical magnetic field theoretically maximizes τ_g (where ψ = 90° relative to the field vector), scaling analysis indicates that τ_g remains negligible in our system due to the minute volume of individual FBTO NWs (~10^{-20} m^3). Thus, gravitational effects are considered insignificant compared to magnetic and elastic forces (Figure 2f; Figure S9e), and the system dynamics are primarily driven by the competition between paramagnetic and elastic torques.

The primary driving force for alignment, the anisotropic paramagnetic torque (τ_{pm}), arises from the shape anisotropy of the NWs and is expressed as:

$$\tau_{pm} = \frac{2}{3}\pi\left[\left(\frac{l}{2}+t\right)\left(\frac{d}{2}+t\right)^2\right]\mu_0\frac{\chi^2}{\chi+2}H_0^2\sin 2\theta \tag{2}$$

Here, l and d are the length and diameter of FBTO NW, μ_0 is the magnetic permeability of free space corresponding to $4\pi\cdot 10^{-7}\ m\cdot kg/s^2A^2$, H_0 is the external magnetic field strength, and θ is the angle between the NW long axis and the direction of applied magnetic field. The

effective magnetic susceptibility (χ) is given by $\chi = C \cdot \chi_{\mathrm{Fe_3O_4}}$, where C is the surface coverage of Fe_3O_4 NPs on FBTO NWs. Surface coverage (C) was quantified via image analysis (ImageJ software) of over 2,700 Fe_3O_4 NPs across nine individual NWs from three independent synthesis batches. C is defined as the area ratio ($A_{\mathrm{Fe_3O_4}}/A_{NW}$) ranging from 0 to 1. Note that FBTO NWs are randomly dispersed before solidification, thus individual FBTO NWs can reveal different rotation angles from initial states. The representative torques under a field were considered as the ones where FBTO NWs in a perpendicularity rotate into the parallel to a magnetic field direction (0° → 90°). By assuming that, we calculated a field strength in which the majority of FBTO NWs contributes to the magnetic alignment. Meanwhile, the magnitude of τ_{pm} exhibits a strong angular dependence due to the sinusoidal term, $\sin 2\theta$. The torque diminishes rapidly as the NW aligns parallel to the field (θ → 90°). Because the magnetic driving force becomes negligible near this equilibrium state, achieving perfect 0°-alignment is physically constrained. Accordingly, we defined the experimental alignment criterion with an angular tolerance of ±3°.

Opposing this magnetic alignment is the elastic restoring torque (τ_{el}), which represents the resistance exerted by the polymer matrix against rotation and is modeled as:

$$\tau_{el} = \theta\left(\frac{d}{2}\right)^3 \frac{2\pi G(1-\upsilon)}{3(3-4\upsilon)\ln\left(l/\frac{d}{2}\right)} \tag{3}$$

where G is the shear modulus and υ is the Poisson's ratio of the dispersing medium. To ensure calculation accuracy, the dynamic shear moduli for various media were experimentally determined using a rheometer (ARES-G2, TA Instrument).

It is considered that thermal fluctuations (Brownian motion) typically oppose alignment in colloidal systems. Furthermore, due to the anisotropic geometry of the FBTO NWs, the suspension can undergo a phase transition to a liquid crystalline (nematic) state. Above a specific concentration, the packing entropy of rod-shaped colloids promotes spontaneous ordering, which could compete with or complicate the magnetic alignment. The theoretical threshold concentration (Φ) for the nematic phase transition is governed by:

$$\frac{1.7\pi d}{l} < \Phi < \frac{1.925\pi d}{l} \tag{4}$$

Our calculation reveals that the bi-phase state is reached at ~98 vol%. Since the concentrations used in this study (0.19 – 7.38 vol% even after the solidification) are significantly below this limit, we concluded that packing entropy effects are also negligible and thereby allow us to focus solely on the competition between paramagnetic and elastic restoring torques.

Experimental validation

While predicting the exact dynamics of multiple anisotropic nanowires involves complex hydrodynamic coupling and dipole-dipole interactions, the single-nanowire model provides a highly valid approximation for determining the initial threshold regime of alignment. The FBTO NWs are initially dispersed at very low volume fractions (0.018 to 0.725 vol%) with superparamagnetic Fe_3O_4 nanoparticles exhibiting near-zero remanent magnetization (~0.14 emu g^{-1}), which prevents spontaneous magnetic agglomeration prior to external field application. During the critical onset stage, the primary rotational resistance is the substantial elastic restoring torque (τ_{el}) of the highly viscous polymer matrix directly enveloping each individual nanowire. Thus, the initial rotation is predominantly governed by the competition between the individual paramagnetic driving torque (τ_{pm}) and matrix elasticity, operating largely independently of multi-body interferences. Although intense multi-wire interactions dominate at much higher fields (e.g., 1500 mT) to drive hierarchical bundling, the single-nanowire analytical framework remains physically robust for predicting the initial threshold activation.

To experimentally verify this theoretical framework, we modeled two distinct viscoelastic regimes that represent extreme boundaries of matrix elasticity. In the paramagnetic-torque-dominant regime, such as an aqueous suspension where the shear modulus is negligible (G_{H_2O} = ~10^{-8} MPa), the elastic restoring torque is virtually non-existent. Consistent with our model's prediction of easy alignment, we observed the onset of axial alignment at a very low field of ~2.5 mT (Figure S9). Furthermore, by synthesizing NWs with varying Fe contents (1.0, 2.5, 7.5, and 10.0% mol/mol to Ba), we confirmed that the critical field decreases as Fe content increases (from 224.09 to 1.19 mT; Figure S9g). These results show excellent agreement with the susceptibility-dependent term in Equation (2).

In the elastic-torque-dominant regime based on a solid thermoplastic polyurethane (TPU, BASF Elastollan 1185A10) matrix, the high modulus yields a prohibitive τ_{el} of ~2.49×10^{-12} N·m. Our calculations indicated that aligning NWs in solid TPU at room temperature would require a practically inaccessible field of 29 T (Figure S10a). However, the model suggested that reducing the shear modulus (G) via heating would enable alignment (Figure S11). Under a constant 1500 mT field, alignment was triggered only when the temperature exceeded 160 °C, the point at which the thermally reduced elastic torque (1.20×10^{-15} N·m) was finally surpassed by the paramagnetic torque (4.74×10^{-15} N·m). While minor deviations in alignment persist due to inherent polydispersity, the overall trend robustly aligns with the model (see Figure 2h and Figure S8c). This framework demonstrates broad versatility that enables the prediction and

control of alignment across various polymer matrices (SMP, PDMS, EcoFlex) by tuning the applied field relative to the matrix's rheological properties.

Supplementary Note 2: Theoretical analysis of load transfer and anisotropic stress confinement to FBTO NWs in polymer composites

Mechanism of stress concentration

Based on classical load transfer theory [2], assuming a perfect interfacial interaction between a polymeric matrix and FBTO NWs, an external mechanical load exerted on the composite system initially propagates through the continuous polymer matrix. Upon encountering the interface, this load is transferred to the dispersed NWs via interfacial shear stress. Critically, because piezoelectric ceramic NWs exhibit a mechanical stiffness significantly higher than that of the viscoelastic polymer, the mechanical stress is not distributed uniformly but is effectively focused and confined within the stiffer NWs. This stress concentration is the fundamental driver for efficient mechanical-to-electrical energy conversion.

Modeling load transfer dynamics

The efficiency of this stress confinement is governed by the load transfer length (l_t), defined as the critical distance required for the interfacial shear stress (τ_y) to build up to the maximum stress (σ_{Max}) within the NW. To simulate the impact of alignment, we modeled composite system under two orthogonal loading conditions: (1) a longitudinal mode (load parallel to the NW long axis; Figure S16e) and (2) a transverse mode (load perpendicular to the NW long axis; Figure S16f). For the simulation, σ_{Max} was set to 0.175 GPa, consistent with the maximum compressive pressure performed in our tests. The NW dimensions were a long-axis length of 1040 nm and a short-axis length of 150 nm (Figure S16e,f).

The load transfer length is derived from the force equilibrium at the interfaces:

$$\tau_y \left(\frac{l_t}{2}\right) \pi d = \sigma_{Max} \left(\frac{\pi d^2}{4}\right) \tag{5}$$

Rearranging this yields the critical length equation:

$$l_t = \frac{\sigma_{Max} d}{2\tau_y} \tag{6}$$

where *d* represents the effective diameter of the NW relative to the loading direction.

More simplified form can be also accepted as below:

$$\sigma_{Max} = 2\tau_y (1 + \upsilon) \tag{7}$$

And we calculated the stress profiles for each orientation by employing Equation (7) with a Poisson's ratio of 0.23 for BTO.

Numerical simulation results and discussion

The simulation reveals a remarkable contrast in stress confinement efficiency dictated by the geometric orientation. In the longitudinal mode, where the load aligns with the NW long axis, the effective diameter corresponds to the short-axis length (*d* = 150 nm). Therefore, the stress builds up rapidly and reaches saturation within a short load transfer zone ($l_t/2$) of approximately 97.5 nm. Since the total length of the NW (1040 nm) significantly exceeds this transfer length ($l \gg l_t$), approximately 82% of the FBTO NW volume resides within the maximum stress region that results in maximizing piezoelectric polarization (Figure S16e).

Whereas, in the transverse mode, the effective diameter expands to the long-axis length (d = 1040 nm). Theoretically, this geometry necessitates a much longer propagation distance of approximately 645.8 nm to achieve maximum stress confinement. However, the physical dimension available in the direction is limited to the NW actual diameter (150 nm), meaning the transfer length far exceeds the available length ($l \ll l_t$). As a result, the stress profile fails to saturate and reaches a peak value of only ~20.3 kPa that is nearly one order of magnitude lower than the parallel case (Figure S16f).

These theoretical insights are in good agreement with the experimental tensile tests (Figure S16b,c) and FEA analysis (Figure 4a; Figure S14). These results confirm that magnetic alignment facilitates anisotropic mechanical properties by optimizing the effective load transfer length along the specific stress direction.

Supplementary Note 3: Theoretical modeling and experimental validation of shape memory mechanics based on elastic beam theory

Elastic beam theory and recovery torque

To accurately predict the actuation performance of the soft robot, we modeled the shape memory behavior using Euler-Bernoulli beam theory. An elastic beam under bending deformation produces a restoring moment proportional to its stiffness and curvature. However, for a shape-memory polymer activated by heat, the constitutive relationship must account for the temperature-dependent change in modulus. The bending moment (M) is thus expressed as the product of the temperature-dependent storage modulus (G'), the second moment of inertia ($I = \frac{wt^3}{12}$, w and t are width and thickness of a beam), and the curvature (κ). Since I scales with the cube of the thickness (t^3) and κ is the reciprocal of the bending radius (R), theoretical analysis suggests that thicker films programmed with smaller bending radii will generate significantly higher recovery torques (Figure S24a). This scaling law provides a fundamental design strategy for tuning the actuation force.

Experimental verification of torque generation and thermo-mechanical proprioception

Based on this theoretical insight, we constructed a comprehensive experimental matrix consisting of thirty-three specimens (area = 1.35 × 1.35 cm^2) with varying thickness (20–310 μm) and programming radii (23.5–216.8 μm) (Figure S24b). Each specimen was programmed, placed on a flat surface, and triggered thermally at 60 °C. The generated recovery force was measured with a digital force gauge (M5-10, Mark-10) equipped with a tip-type accessory. The experimental setup was standardized. The pivot was precisely centered at 0.675 cm from the edge via thermal reconfiguration, the gauge tip contacted the specimen at a height of 0.035 cm, and the lever arm was set to 0.35 cm. Then, the measured force was converted into recovery torque (τ_{rec}) using the geometric relation:

$$\tau_{rec} = rF_{meas}\sin(90-\theta) \quad (8)$$

where r is the length of the lever arm, F_{meas} is the force recorded by the gauge, and θ is the instantaneous bending angle. Although the experimental values exceeded simple theoretical predictions by a few orders of magnitude because of complex viscoelastic effects and large-deformation non-linearity, the results confirmed the qualitative trend. Specimens with greater thickness and tighter programming radii generated superior bending moment (Figure S24b).

For a highly reliable quantitative evaluation and establishing a robust empirical backgrounds about piezoelectric response upon thermo-mechanical actuation, we applied an advanced signal acquisition system including a voltage reference buffer follower, as schematically described in

Scheme 1. An IC (OPA2381), dampers, physical guards, and passive filters were integrated between the sensor and the voltmeter to enhance the signal-to-noise ratio (SNR). While the fundamental framework of the single-ended measurement protocol from the main manuscript in Figure 5 was maintained to simulate the original signal acquisition environment, the updated architecture introduces a modified readout convention. Specifically, the modified system acquires the voltage signal based on the formula, $V_{\mathrm{measured}} = V_{\mathrm{out}} - V_{\mathrm{RefBus}}$. Here, V_{measured} denotes the electric potential read by the voltmeter, V_{out} represents the buffered potential from the sensor electrode, and V_{RefBus} corresponds to the potential at the reference busbar node. The 2.5 V reference voltage is derived from an externally supplied 5.0 V bias, a configuration fundamentally required to capture bipolar voltage signals. The integration of these buffered potentials successfully completes a non-referenced single-ended (NRSE) measurement circuit. By extracting the voltage signals through the updated NRSE architecture, the baseline noise offset undergoes successful attenuation from approximately 2.0 mV to 0.2 mV. This precise attenuation resolves the primary obstacle of identifying signal onsets and terminations. Furthermore, external disturbances, such as the 5.0 mV in-rush current generated when activating the heating lamp, are virtually minimized. Thus, the improved SNR provides a highly robust platform for the subsequent quantitative analysis.

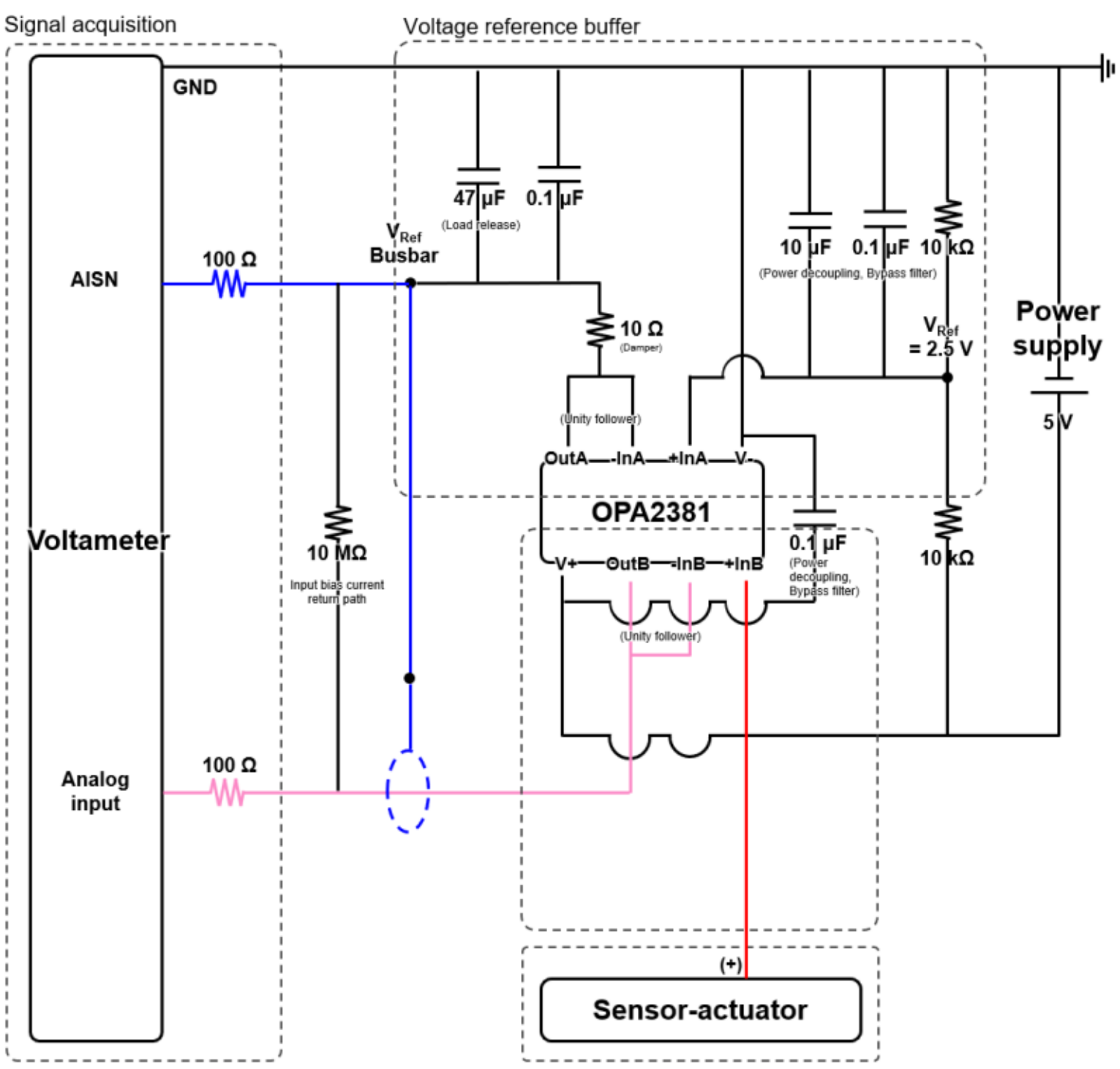

Scheme 1. Schematic illustration of the advanced signal acquisition circuit incorporating a voltage reference buffer follower.

With the updated circuit, we modeled a monolithic sensor-actuator unit without the encapsulation layers and the central obstruction (by the orb) to isolate the intrinsic proprioceptive mechanics from structural/measurement artifacts (found in Figure 5). Figure S28b,c illustrate the time-domain V_{OC} and macroscopic displacement of these isolated units. The data demonstrates that curvature programming induces a large gradient in bending actuation. Specifically, the programmed radii clearly dictate the onset of shape morphing, the displacement rate, and the peak-to-valley V_{OC} amplitude. Furthermore, Figure S28d–f validate the cyclic reliability of this sensing modality. Over continuous actuation cycles, the V_{OC} amplitudes and corresponding bending displacements were mostly maintained, exhibiting with no remarkable degradation in peak amplitude or baseline stability. Notably, the monolithic units used in these isolated tests were thinner (approximately 200 μm) than the fully integrated soft robot (approximately 600 μm in total, in Figure 5). The reduction in overall thickness inherently results in relatively smaller reported amplitudes and displacements due to lower recovery torques.

To clearly isolate these acquired proprioceptive signals from potential signal artifacts such as pyroelectric response of the composites under a thermal condition, we tested a sensor-actuator monolithic unit with programmed and un-programmed states (the size and thickness were same to the typical sensors), and measured their electric responses under a thermal condition. As shown in Figure S31c–f and S32, no noticeable signals were obtained from un-programmed states, which may be because the pyroelectric response is weak and beyond a detection limit. On the other hand, the peak-to-valley V_{OC} values during the shape memory behaviors were increased depending on the programmed curvature (Figure S32), which is appreciated by the findings that higher bending moments produce higher load transfer and the resulting piezoelectric signals.

Payload capacity calculation

Guided by these findings, we derived the payload capacity of the specimens, defined as the maximum weight of a programmed beam can lift against gravity. The critical condition occurs when the generated recovery torque (τ_{rec}) surpasses the gravitational torque exerted by the payload. The governing equation for the payload capacity (W_{load}) at a given actuation angle is:

$$W_{load} = \frac{\tau_{rec}}{gr\sin\theta} \quad (9)$$

where g is gravitational acceleration constant, r is the distance to the payload center of mass, and θ is the angle of elevation. We mapped these calculations onto a design chart (Figure 5e;

Figure S24c), which describes the underloaded (successful lifting) and overloaded (failure) regimes with respect to specimen thickness and programming radius. This mapping was consistent with subsequent verification tests.

Robotic motion design and signal acquisition

Based on this mechanical guideline, we designed a 600-μm-thick robotic platform composed of 300 μm encapsulation and 300 μm body, which is capable of executing two distinct actuation modes.

1) Simultaneous actuation (low torque mode): To facilitate tasks such as enveloping an object or assembling a box, we programmed the robot arms with a relatively large bending radius (~440 μm). This configuration generates a low but uniform bending moment across all arms. Upon thermal triggering, the arms fold simultaneously. This synchronized motion is ideal for lifting light payloads (up to ~200 mg per plane) and achieving precise geometric interlocking for object enclosure (Figure 5a; Figure S25). The measured electromechanical signals (V_{oc}) during this mode exhibited low and comparable amplitudes, which ensures the uniform and lower-magnitude stress generation.
2) Sequential actuation (high torque gradient mode): To operate stepwise motion with distinct signal recognition, we programmed the arms with progressively decreasing radii (e.g. 55, 110, 165, and 220 μm) that induces a large gradient in recovery torque. As a result, the arms actuate sequentially by starting from the arm with the smallest radius (highest torque). This mode produced time-resolved, high-amplitude V_{oc} signals and thereby enabled the distinct recognition of each folding event (Figure 5g–k).

Supplementary Note 4: Quantitative estimation of localized contact mechanics and forces

To quantify this specific relationship, we applied a mathematical calibration model derived from the pre-calibrated linear sensitivity of the z-axis-aligned sensor under normal compression. As demonstrated in Figure 4c-i of the main text, this linear sensitivity (S) was measured at 123 μV kPa^{-1}. Fundamentally, the applied contact force (F) can be calculated from the raw V_{oc} peak magnitude and the dynamically varying contact area ($A_{contact}$) using the following relationship.

$$F = \frac{V_{oc} \times 2}{S} \times A_{contact}$$

During the robotic gripping motion, the contact area between the compliant inner face of each robotic arm and the rigid spherical orb varies dynamically depending on the composite modulus at the triggering temperature and the excess bending moments. Because direct optical measurement of this localized contact area during shape morphing is practically limited, we constructed an analytical framework that couples the recovery torques generated by the shape-memory polymer composite arm with Hertzian sphere-on-flat contact mechanics[3].

Applying this analytical method, the excess recovery torques remaining after bending (yet physically restricted by the orb) are converted into a normal contact area using the contact moment arms. Briefly, the excess torque (τ_{excess}) is defined by the following equation,

$$\tau_{excess} = \left(W_{capacity} - W_{payload}\right) g r_{load} \sin\theta$$

where $W_{payload}$ is approximately 0.1863 g corresponding to the mass of one single arm. Other associated parameters are detailed in Supplementary Note 3. Subsequently, the Hertzian contact force ($F_{contact}$) is approximated as,

$$F_{contact} = \frac{\tau_{excess}}{r_{contact}}$$

where $r_{contact}$ stands for the contact moment arm, measuring exactly 7.217 mm determined from the geometric configurations of the robotic arm and the central orb. To estimate the localized contact force in a highly rigorous manner, we approximated the contact area using the reduced-modulus Hertz contact relation for a sphere-on-flat interface[3]. Upon circular contact, higher loads and relative radii produce a larger contact area, whereas higher contact moduli result in a smaller contact area. According to this established Hertzian contact theory, the contact radius ($a_{contact}$) is determined by the following formulation.

$$a = \left(\frac{3F_{contact}\, a_{orb}}{4E^*}\right)^{1/3}$$

When the reduced contact modulus (E^*) is defined as,

$$E^* = \left[\frac{1 - \upsilon_{composite}^2}{E_{composite}} + \frac{1 - \upsilon_{orb}^2}{E_{orb}}\right]^{-1}$$

For this formulation, $E_{composite}$ was substituted with the modulus of the 10 wt% composite at 60 °C obtained from the slope in the initial range of dynamic strain derived from thermo-mechanical analysis. For the rigid orb, the standard modulus value of 75 GPa for glass materials was adopted for E_{orb}. Lastly, by obtaining $a_{contact}$ from the aforementioned derivation, the circular contact area ($A_{contact}$) was calculated from

$$A_{contact} = \pi a_{contact}^2$$

The calculated contact areas for each robotic arm, which vary systematically according to their programmed bending radii, are presented in Figure S30a. Finally, by applying this analytically derived contact area to our established benchmark in Figure 4c-i, the raw piezoelectric voltage is successfully translated into the quantified localized contact force, as summarized in Figure S30b.

We should note that while this theoretical model provides a highly logical framework for force quantification, the exact deduction of the localized contact forces remains subject to inherent analytical variances. These specific variances primarily originate from the thermal fluctuations influencing the dynamic viscoelastic behaviors of the polymer matrix.

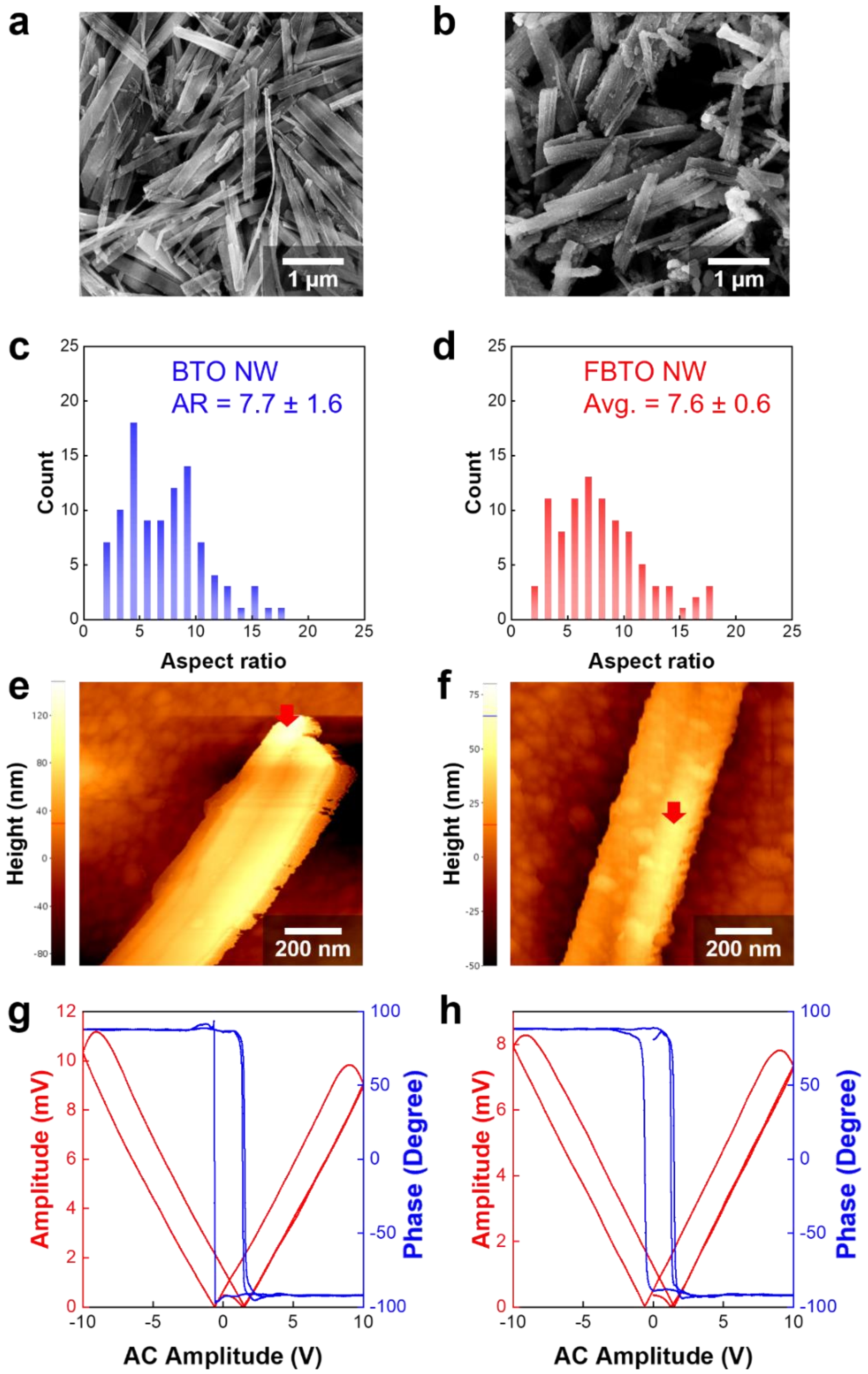

Figure S1. Morphological and electromechanical characterization of pristine BTO and FBTO NWs. (a–h) Comparative analysis of pristine BTO NWs (a,c,e,g) and FBTO NWs (b,d,f,h). (a,b) High-resolution SEM images showing surface morphology. (c,d) Statistical distributions of ARs. (e,f) Two-dimensional topographical profiles obtained by atomic force microscopy (AFM). Red arrows indicate the specific locations where point-mode electromechanical measurements were acquired. (g,h) Local piezoelectric response signals measured via piezoresponse force microscopy (PFM).

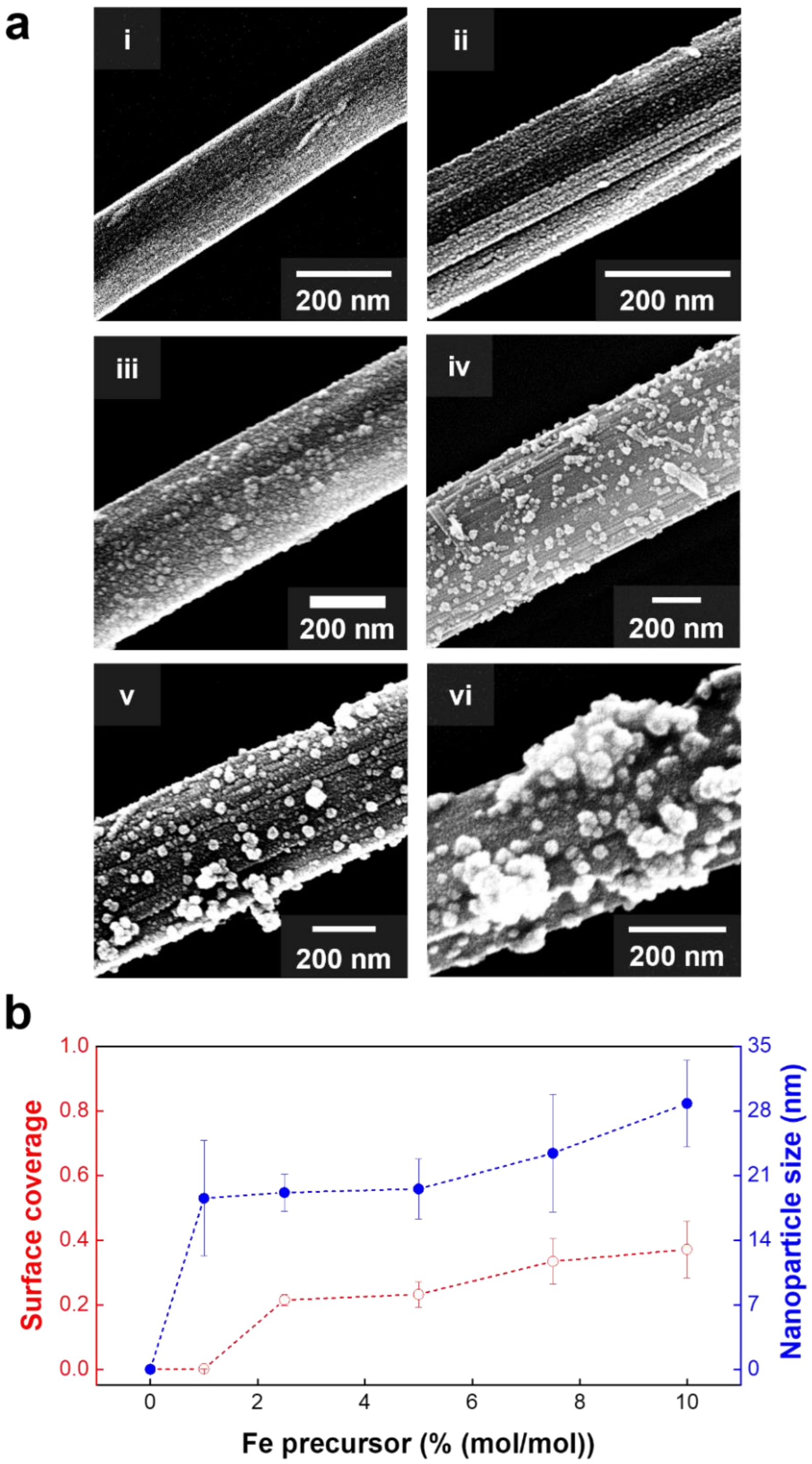

Figure S2. Morphological evolution of FBTO NWs with varying Fe precursor concentrations. (a) High-resolution scanning electron microscopic (HR-SEM) images of FBTO NWs synthesized with increasing Fe:Ba molar ratios: (i) 0% (mol/mol), (ii) 1% (mol/mol), (iii) 2.5% (mol/mol), (iv) 5% (mol/mol), (v) 7.5% (mol/mol), (vi) 10% (mol/mol). (b) Corresponding statistical analysis of the average particle size and surface coverage of Fe_3O_4 NPs on the BTO NW surface.

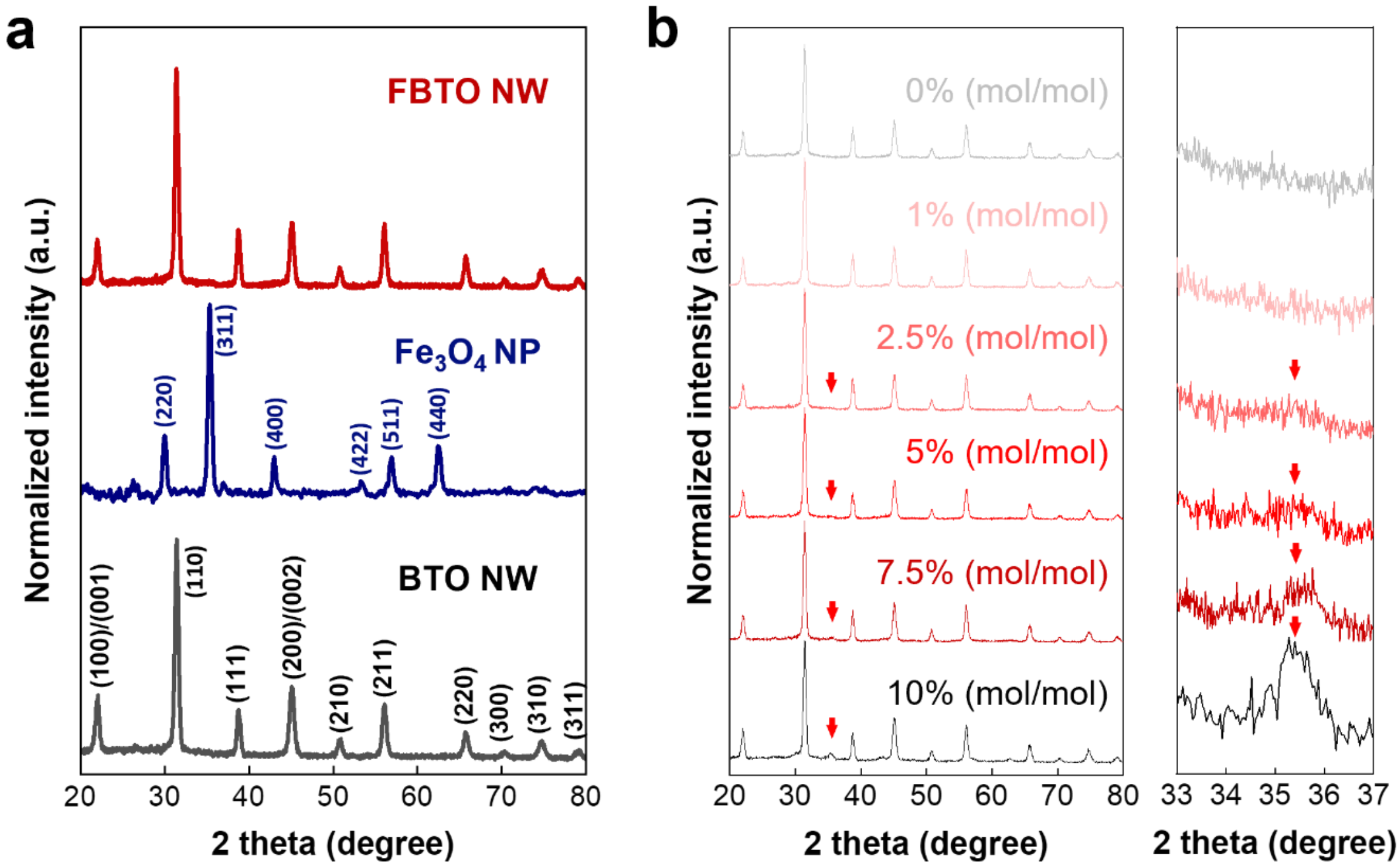

Figure S3. Crystallographic structural analysis of FBTO NWs. (a) Comparative high-resolution X-ray diffraction (HR-XRD) patterns of pristine BTO NWs, pure Fe_3O_4 NPs, and FBTO NWs. (b) HR-XRD patterns of FBTO NWs synthesized with Fe:Ba molar ratios ranging from 0 to 10% (mol/mol). Red arrows indicate the characteristic diffraction peaks corresponding to the Fe_3O_4 phase.

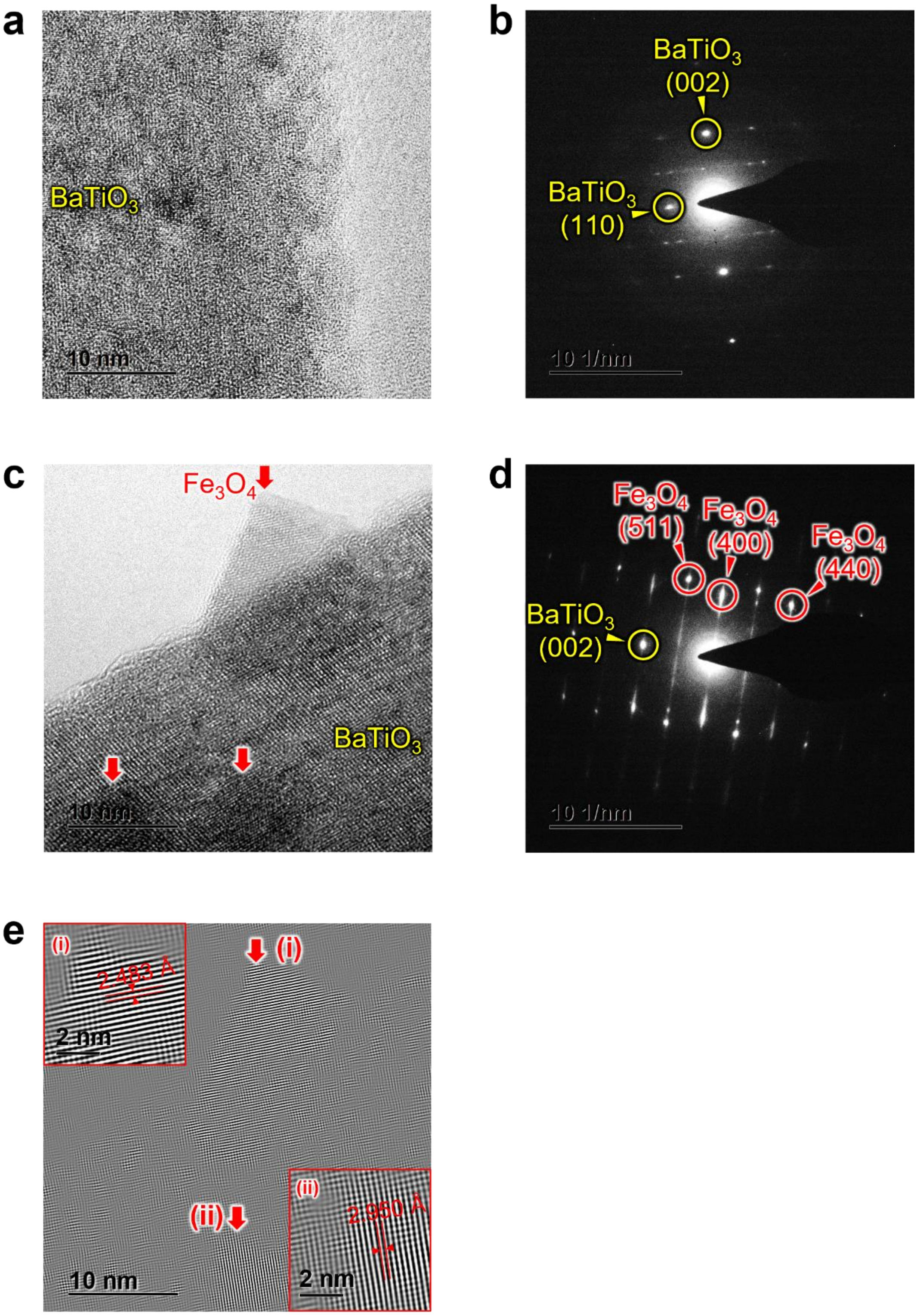

Figure S4. Crystallographic characterization of BTO and FBTO NWs. (a–d) High-resolution TEM (HR-TEM) images and corresponding selected-area electron diffraction (SAED) patterns of pristine BTO NWs (a,b) and FBTO NWs (c,d). (e) Fourier-filtered image derived from (c), highlighting the (311) and (220) lattice planes characteristic of the Fe_3O_4 phase.

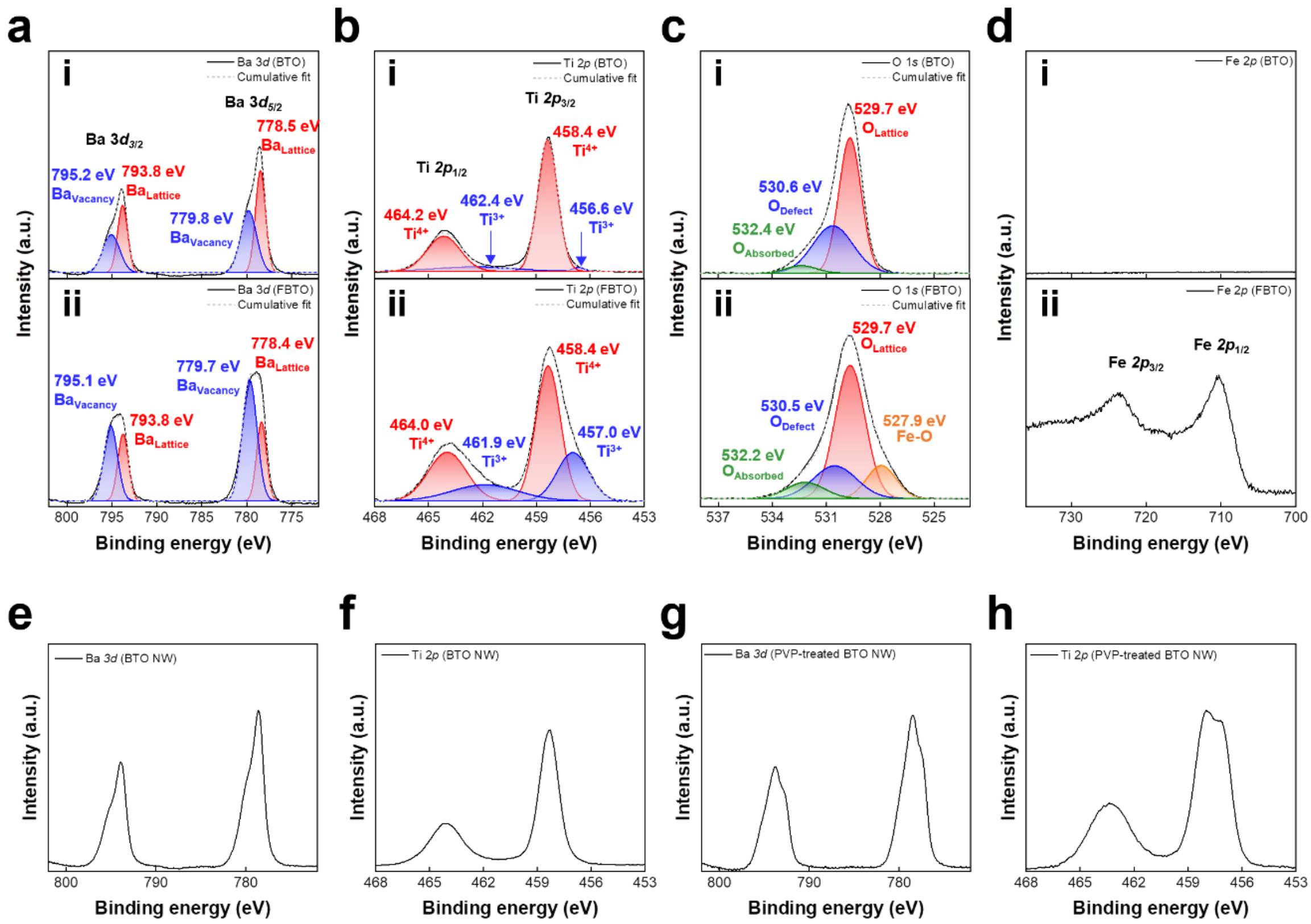

Figure S5. Surface chemical analysis via X-ray photoelectron spectroscopy (XPS). (a–d) Comparative high-resolution XPS spectra and deconvoluted peaks of pristine BTO NWs (i) and FBTO NWs (ii) covering the (a) Ba *3d*, (b) Ti *2p*, (c) O *1s*, and (d) Fe *2p*. (e–h) Corresponding XPS spectra of PVP-functionalized BTO NWs prior to Fe_3O_4 deposition, showing (e) Ba *3d*, (f) Ti *2p*, (g) O *1s*, and (h) Fe *2p* regions to elucidate the surface-mediated growth mechanism.

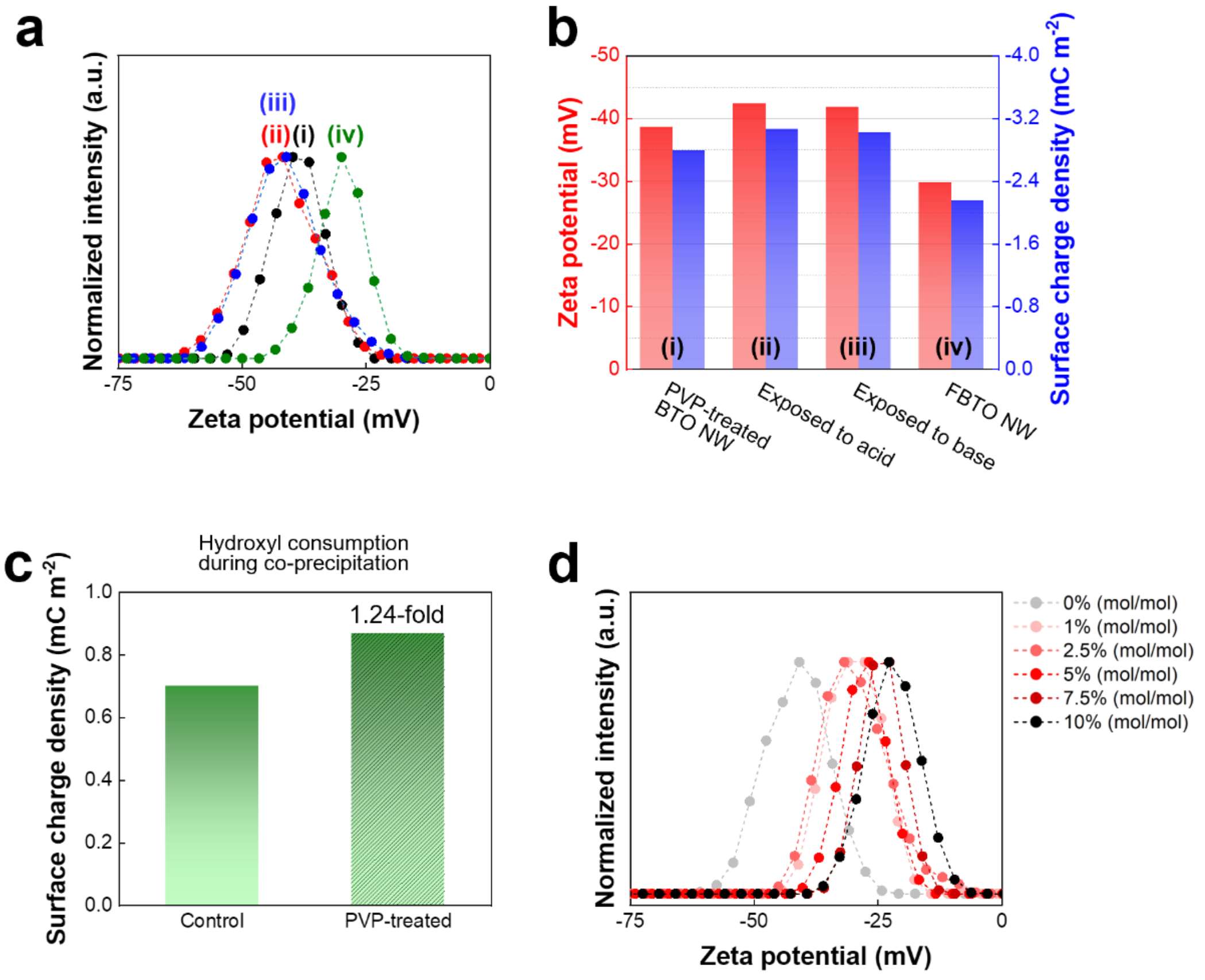

Figure S6. Surface charge analysis and growth mechanism verification. (a) Zeta potential profiles of (i) PVP-treated BTO NW, (ii) acid-exposed BTO NW, (iii) base-exposed BTO NW, and (iv) FBTO NW. (b) Gaussian-fitted values of zeta potential and surface charge density calculated from Graham equation based on the fitted values. (c) Consumed negatively charged surface moieties of pristine BTO NW and PVP-treated BTO NW after co-precipitation reaction of Fe_3O_4 nanoparticle. (d) Zeta potential profiles of FBTO NWs with a molecular ratio of Fe/Ba ranging from 0–10% (mol/mol).

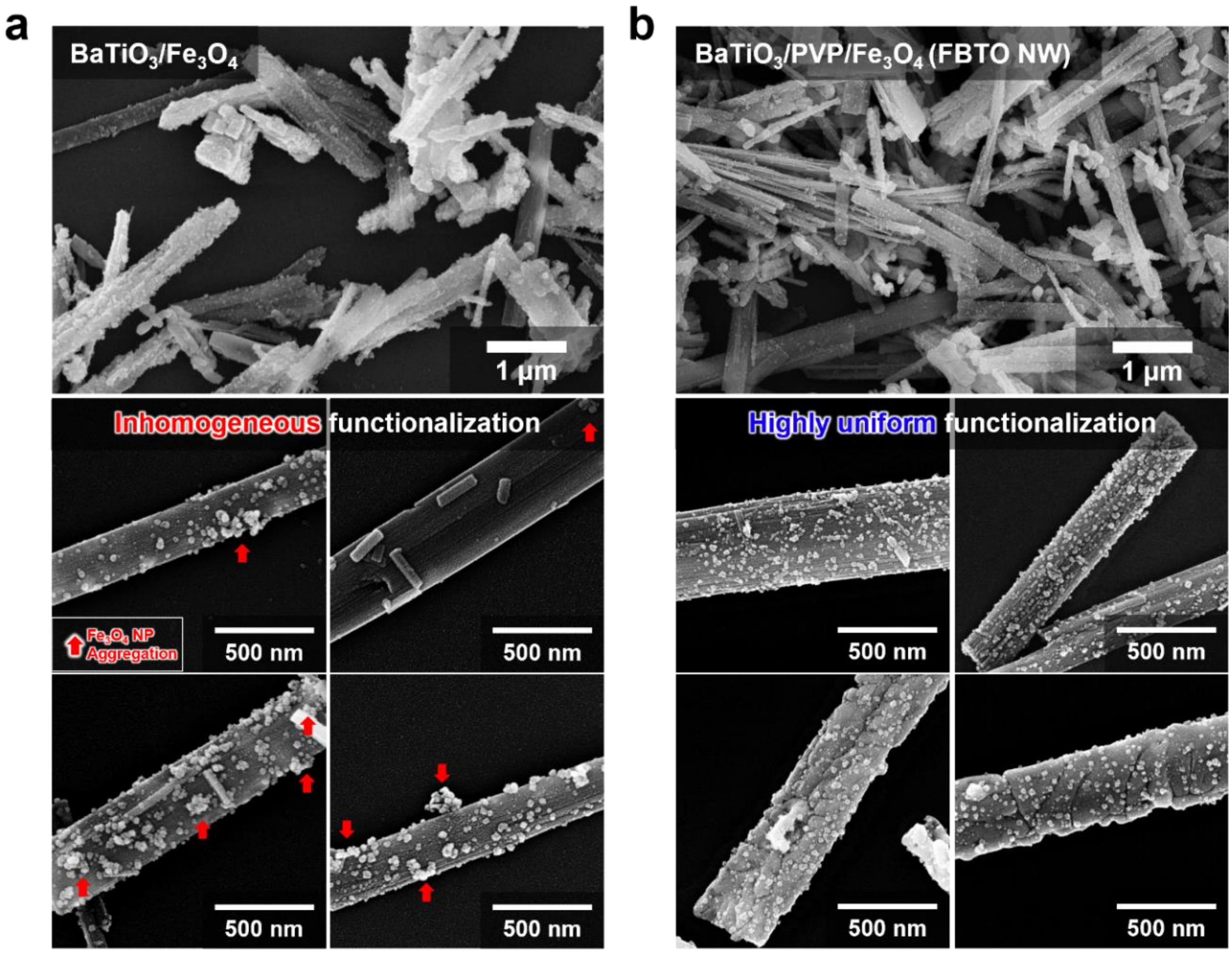

Figure S7. Impact of PVP functionalization on surface morphology. HR-SEM images of Fe_3O_4 NP-decorated BTO NW (a) without PVP treatment and (b) with PVP treatment.

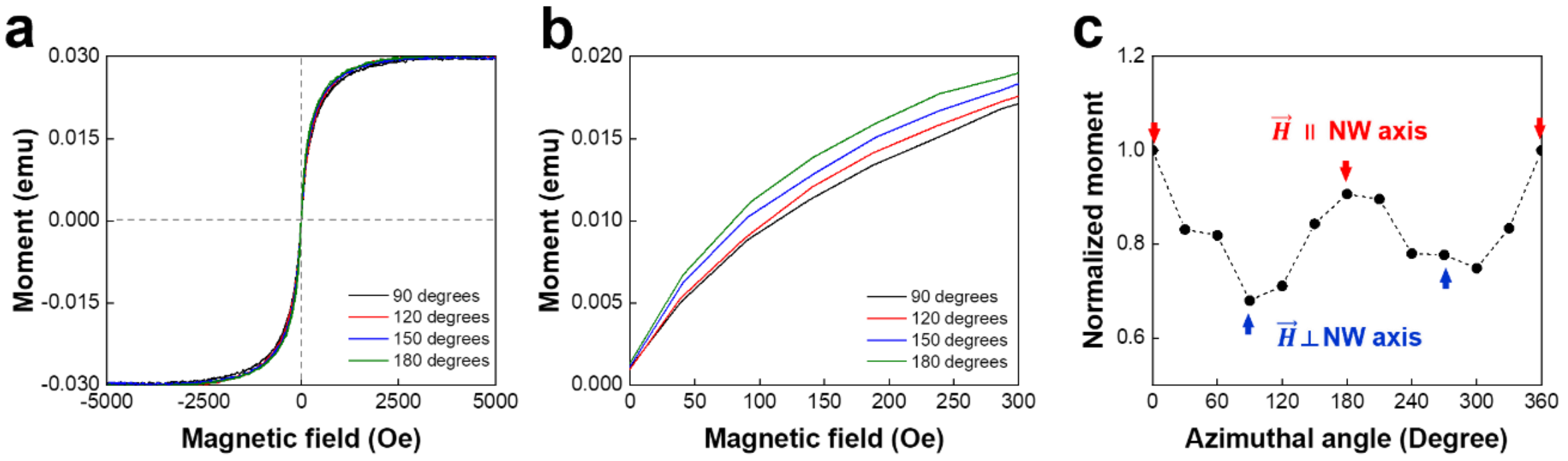

Figure S8. Magnetic anisotropy of aligned FBTO NWs. (a) Magnetic hysteresis curves of magnetically aligned FBTO NW in horizontal direction, which are obtained from different in-plane azimuthal angles (0–180 degrees). (b) Magnified views of magnetic hysteresis in an initial region of an external magnetic field. (c) Normalized magnetic moments depending on in-plane azimuthal angles.

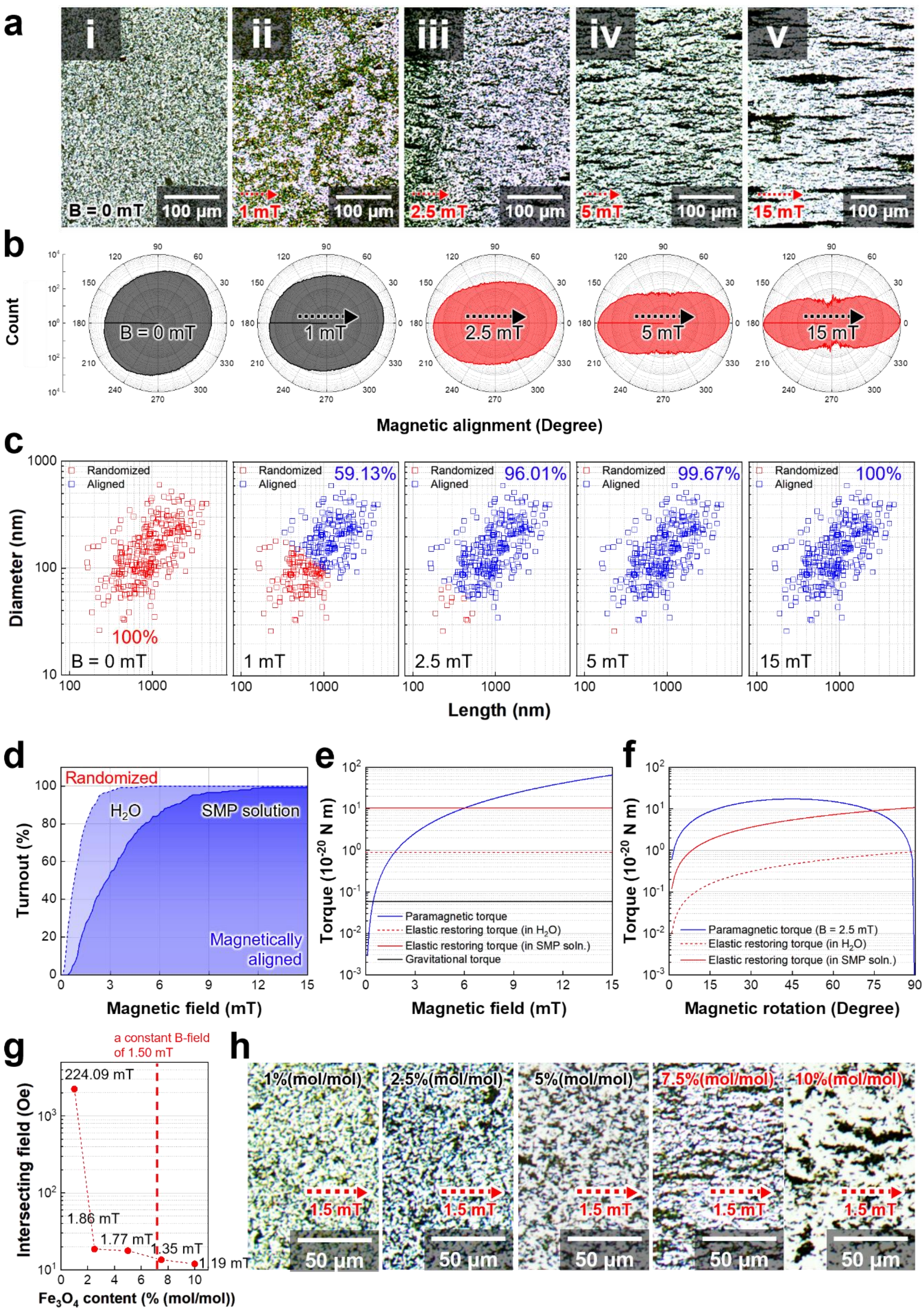

Figure S9. Alignment dynamics and torque analysis in different media. (a–c) Magnetic alignment behavior of FBTO NW aqueous suspensions. (a) Optical micrographs corresponding

(b) angular orientation profiles and (c) theoretical prediction of alignment states under magnetic fields ranging from 0 to 15 mT. (d) Comparative evolution of the aligned NW population in low-viscosity aqueous medium (dotted lines) versus high-viscosity SMP/DMSO solution (solid lines). (e,f) Theoretical torque balance analysis. (e) Comparison of anisotropic paramagnetic torque (blue) against gravitational (black) and elastic restoring torques in water (red dotted) and SMP solution (red solid). (f) Angular dependence of paramagnetic and elastic restoring torques governing the rotational dynamics. (g) Theoretical expectation of magnetic fields required for magnetic alignment of FBTO NWs with a molecular ratio of Fe/Ba ranging from 0 – 10% (mol/mol). (h) Optical micrographs of the low-viscosity aqueous suspensions of FBTO NWs with different Fe/Ba ratio under 1.5 mT.

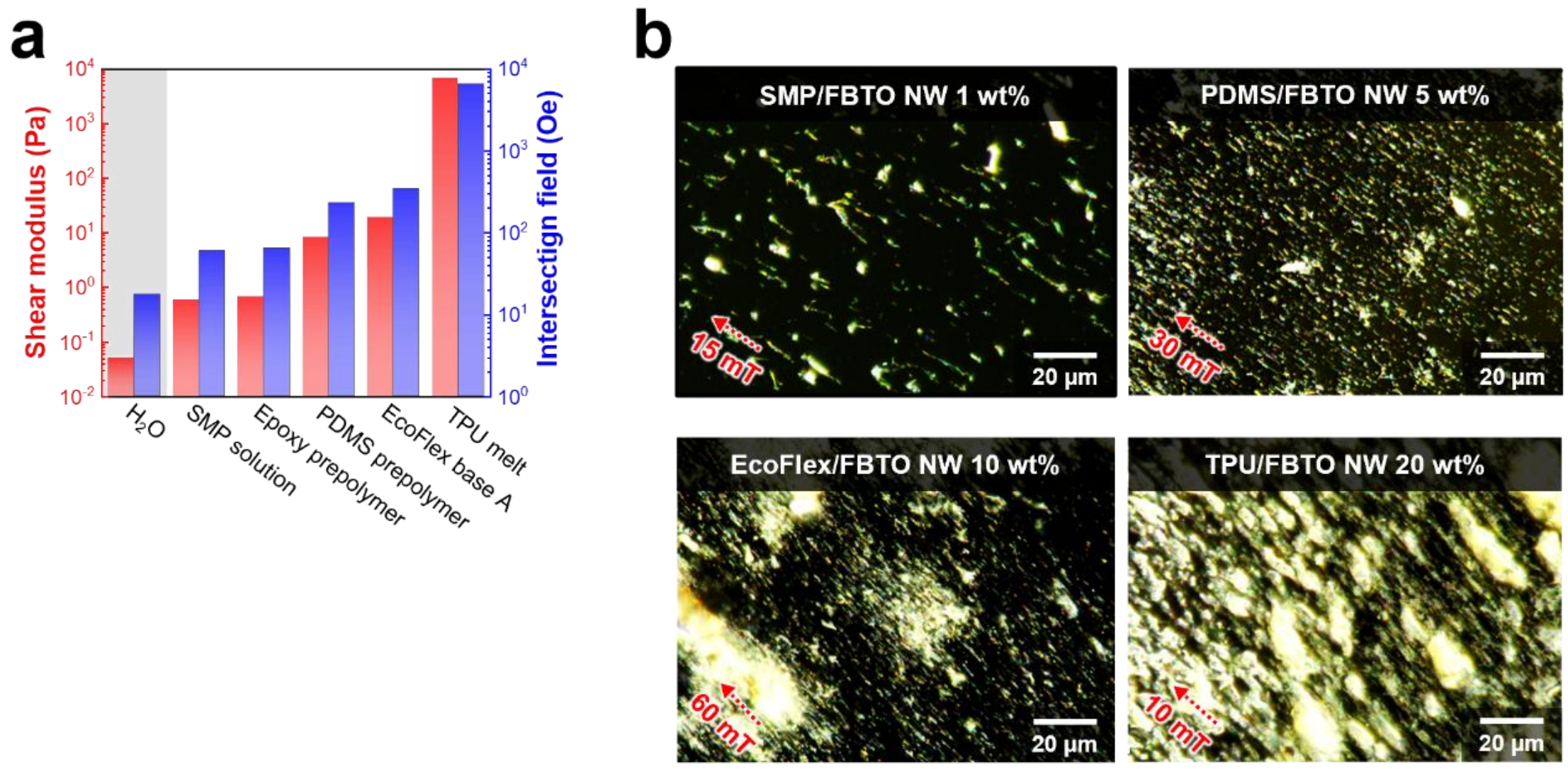

Figure S10. Rheological dependence and versatility of alignment. (a) Dynamic shear moduli (red) and the critical magnetic field required for 90º rotation of FBTO NWs (blue) across various dispersion media. (b) Optical micrographs of FBTO NW composites prepared with diverse polymer matrices, demonstrating the universal applicability of the alignment strategy.

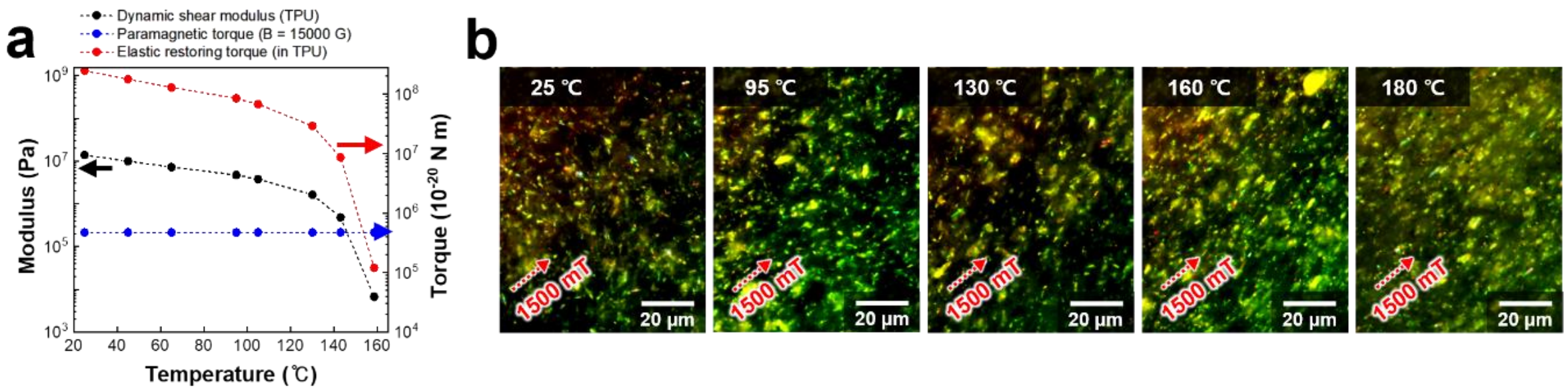

Figure S11. Temperature-dependent alignment mechanism in thermoplastic polyurethane (TPU). (a) Temperature dependence of the dynamic shear modulus (black dotted line), contrasted with the anisotropic paramagnetic torque (blue) and the elastic restoring torque (red) of the TPU matrix. (b) Optical micrographs demonstrating the magnetic alignment transition in TPU/FBTO NW composites induced by heating from 25 to 180 ℃.

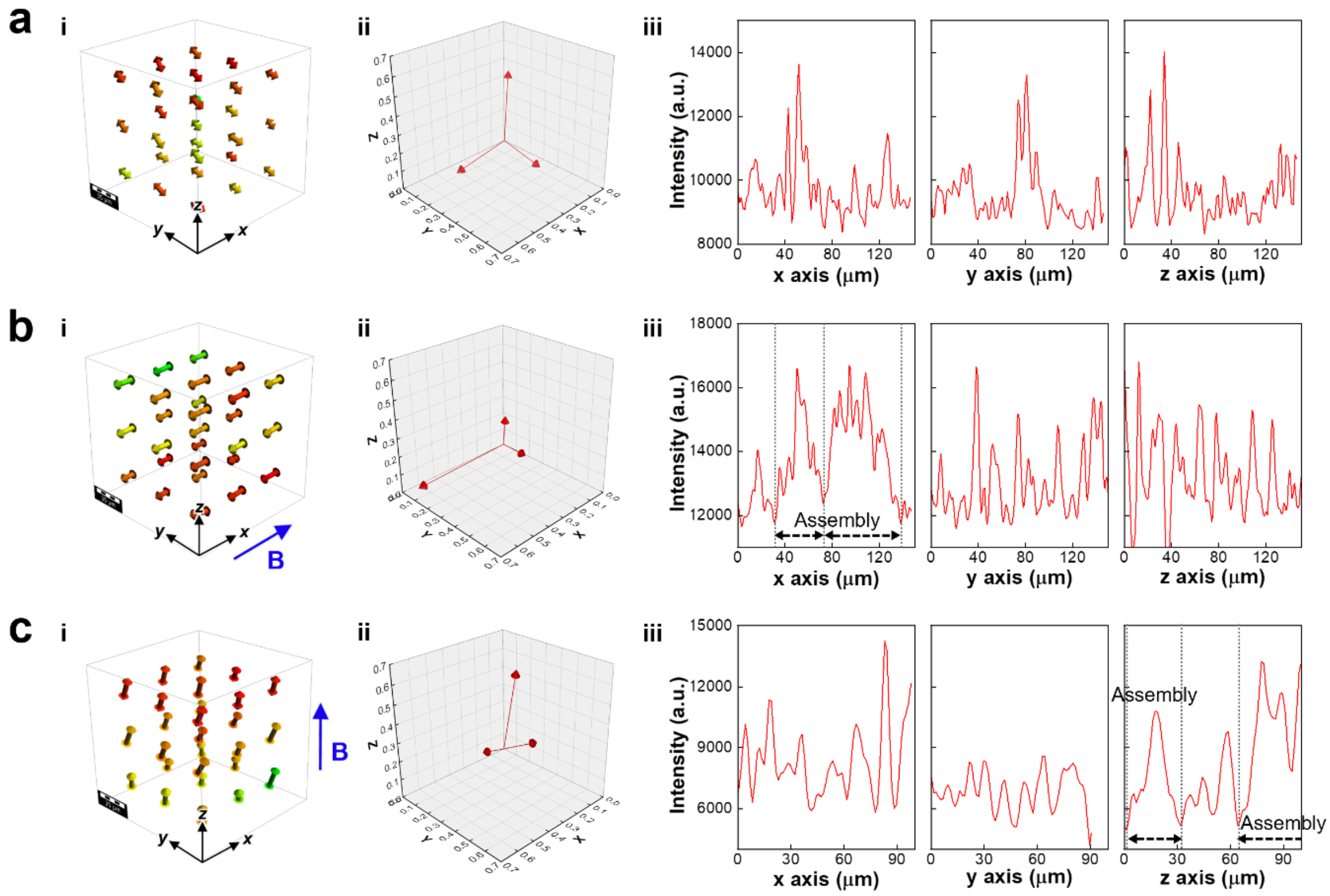

Figure S12. Quantitative 3D structural analysis of NW alignment. (a–c) Comprehensive orientation analysis based on 3D XRM models of composites with (a) random, (b) x-axis-aligned, and (c) z-axis-aligned FBTO NW distributions. Each panel displays (i) 3D fiber orientation intensity plots, (ii) second-order orientation tensors, and (iii) intensity-distance profiles. The color-coded arrows (green to red) in the plots and tensors indicate the direction and degree of predominant orientation. High-intensity regions in the distance profiles correspond to the dense spatial distribution of FBTO NWs, corroborating the formation of chain-like assemblies within the SMP matrix.

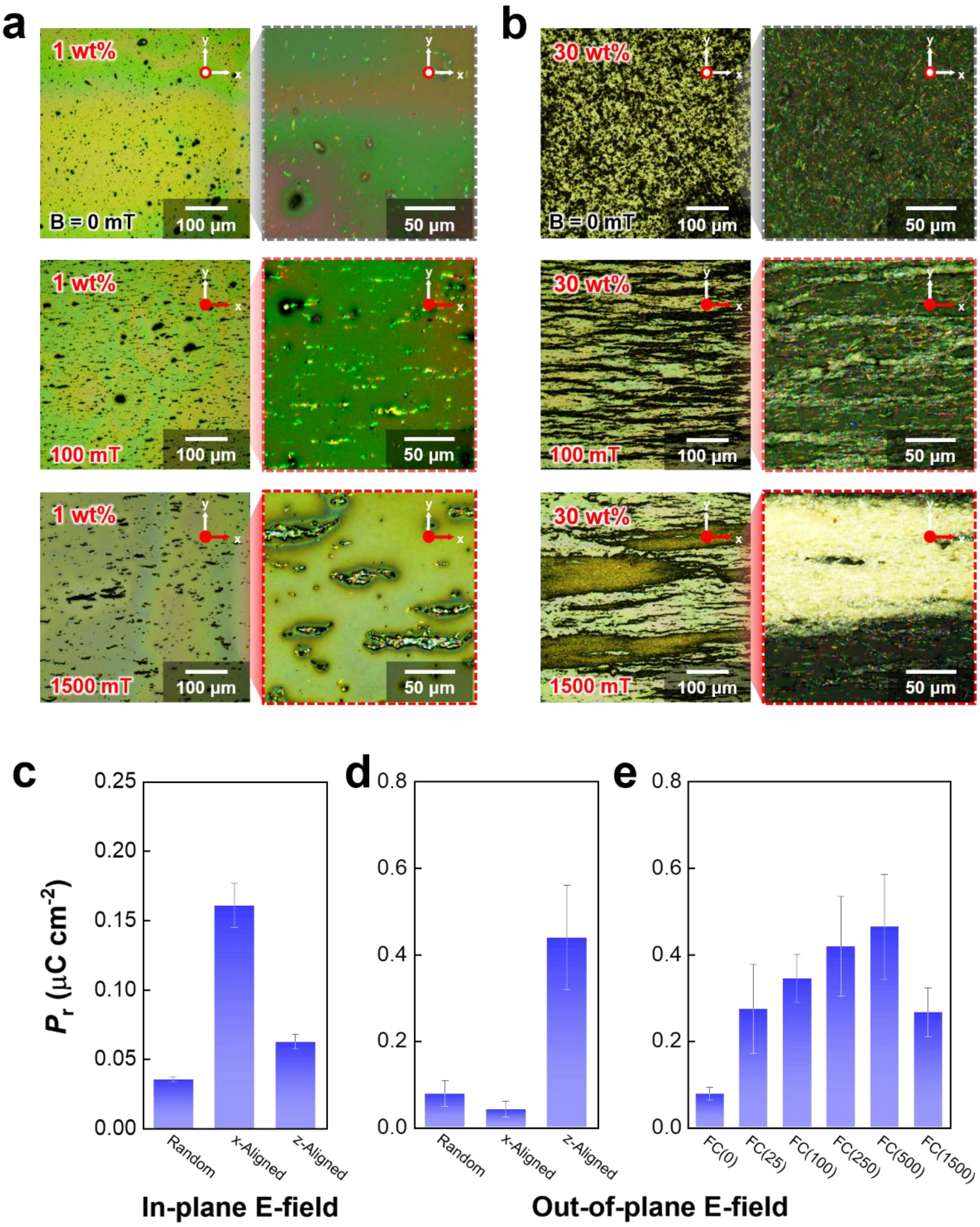

Figure S13. Microstructural evolution and ferroelectric polarization analysis. (a,b) Optical micrographs of thin composite films (thickness = ~10 µm) containing (a) 1 wt% and (b) 30 wt% FBTO NWs, prepared under varying magnetic field strengths (0–1500 mT). (c–e) Ferroelectric characteristics of high-loading (40 wt%) composites, denoted as FC(X) where X indicates the alignment magnetic field strength. (c,d) Remanent polarization (P_r) measured under (c) in-plane and (d) out-of-plane electric fields (E) for different alignment orientations. (e) Evolution of P_r as a function of the magnetic field strength (FC series) measured under out-of-plane electric fields.

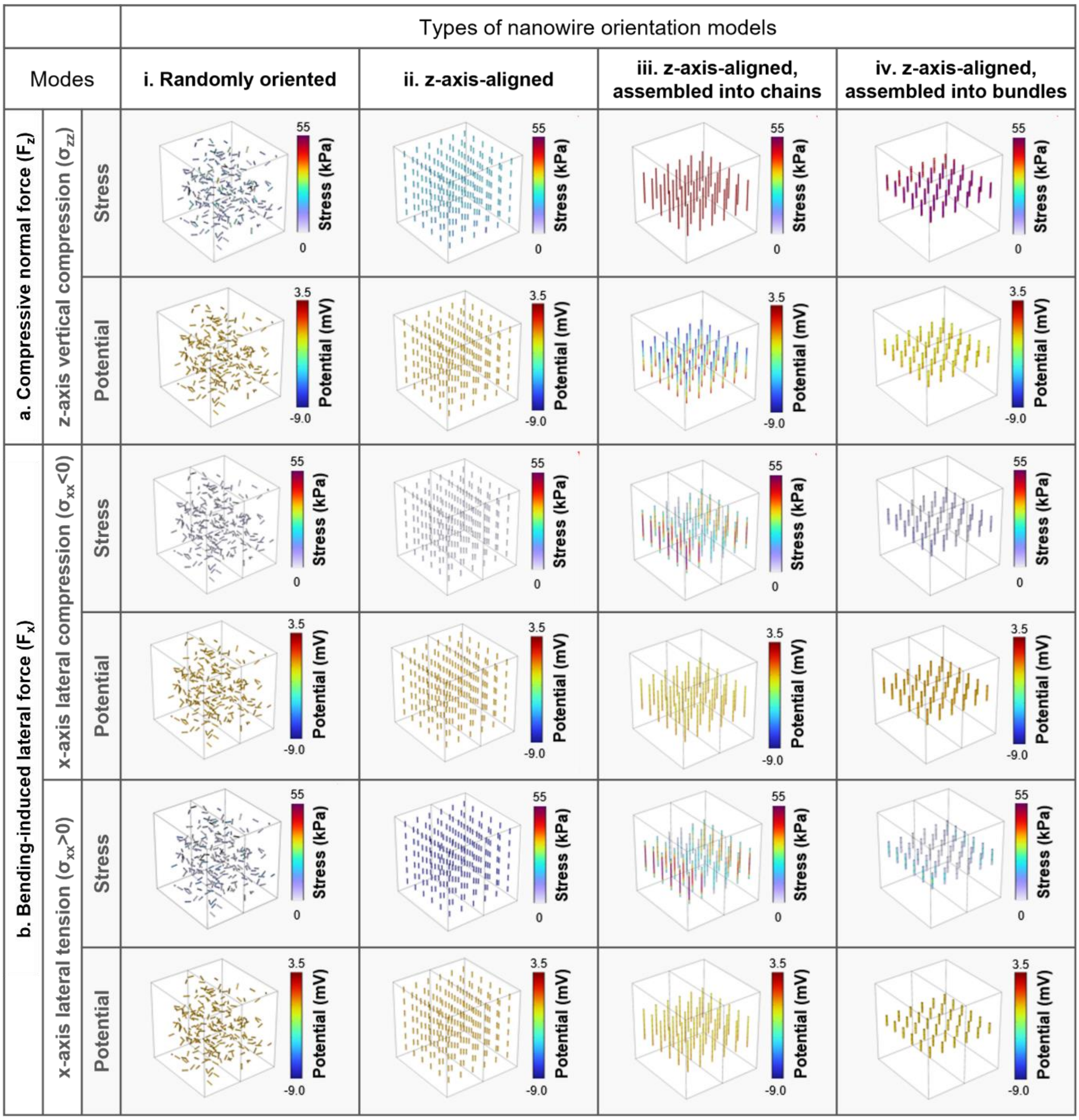

Figure S14. Computational analysis of microstructure-dependent stress transfer. Finite element analysis (FEA) mapping of mechanical stress and piezoelectric potential distributions. The simulations are categorized by the loading direction relative to the z-axis NW alignment: (a) Compressive normal force (F_z) inducing vertical stress (σ_{zz}), representing parallel loading (longitudinal mode). (b) Bending-induced lateral force (F_x) inducing lateral compression (σ_{xx}<0) and tension (σ_{xx}>0), representing perpendicular loading (transverse mode). The polymer matrix is rendered transparent to visualize stress concentration on the nanofillers. Columns (i–iv) compare four representative microstructural configurations: (i) randomly oriented, (ii) z-axis-aligned, (iii) z-axis-aligned chain-like assemblies, and (iv) z-axis-aligned bundles.

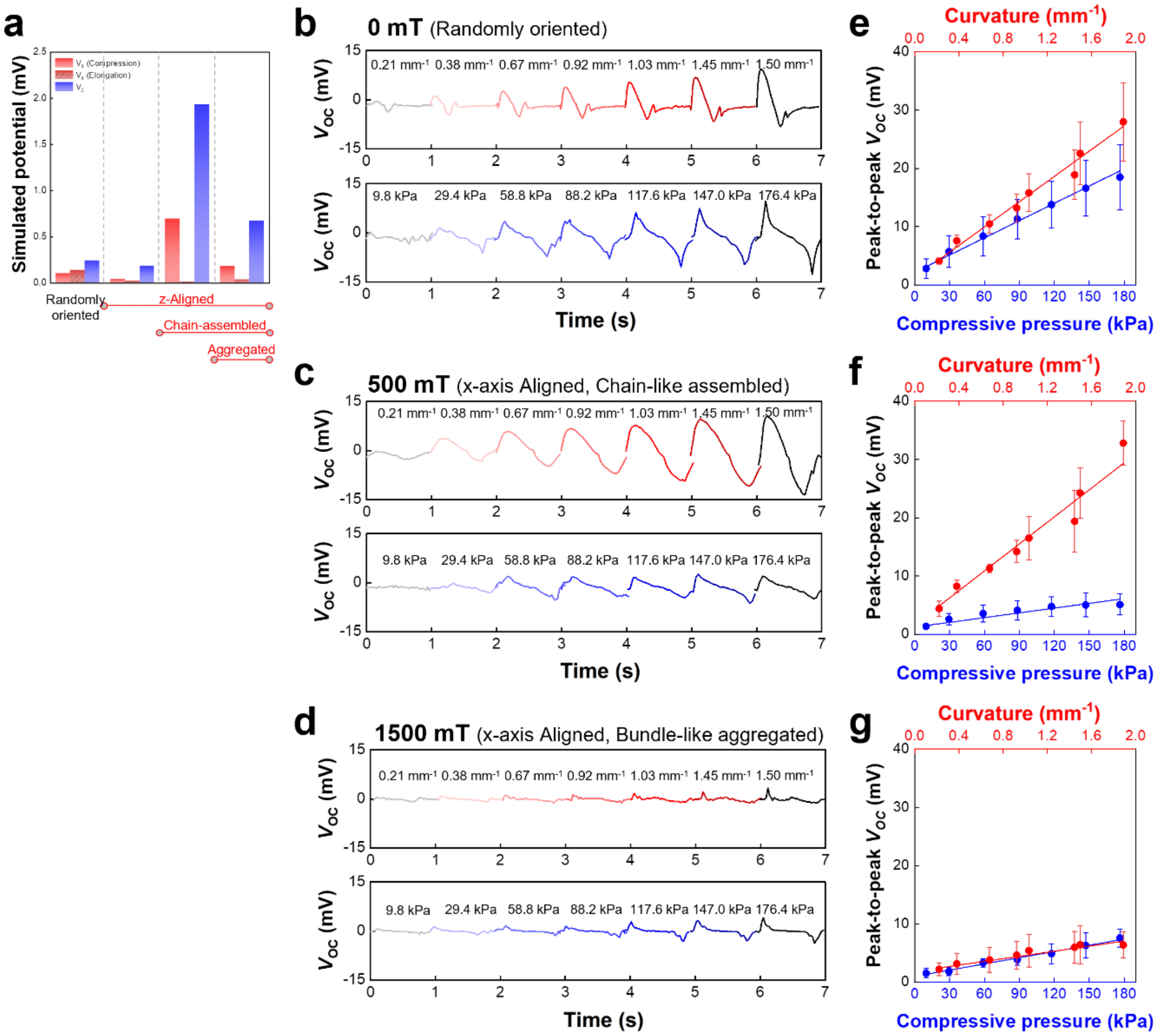

Figure S15. (a) Quantitative comparison of piezoelectric potential generation. FEA-simulated potential outputs for the four representative microstructural models defined in Figure S14 (randomly oriented and z-axis-aligned). The bar charts contrast the electrical response generated under vertical compression (F_z, blue columns) versus bending-induced lateral force (F_x, red columns), highlighting the dependence of electromechanical transduction efficiency on nanowire alignment and assembly. (b-g) Electromechanical responses and signal decoupling capabilities of the magneto-piezoelectric sensors across different nanowire alignment states (randomly oriented and x-axis-aligned). (b-d) Real-time open-circuit voltage (V_{oc}) profiles under applied bending curvature (top panels) and vertical compressive pressure (bottom panels) for composites fabricated under magnetic fields of (b) 0 mT, (c) 500 mT, and (d) 1500 mT. (e-g) Corresponding peak-to-peak voltage outputs as a function of curvature (red lines) and compressive pressure (blue lines) to evaluate the signal decoupling performance for the (e) 0 mT, (f) 500 mT, and (g) 1500 mT states.

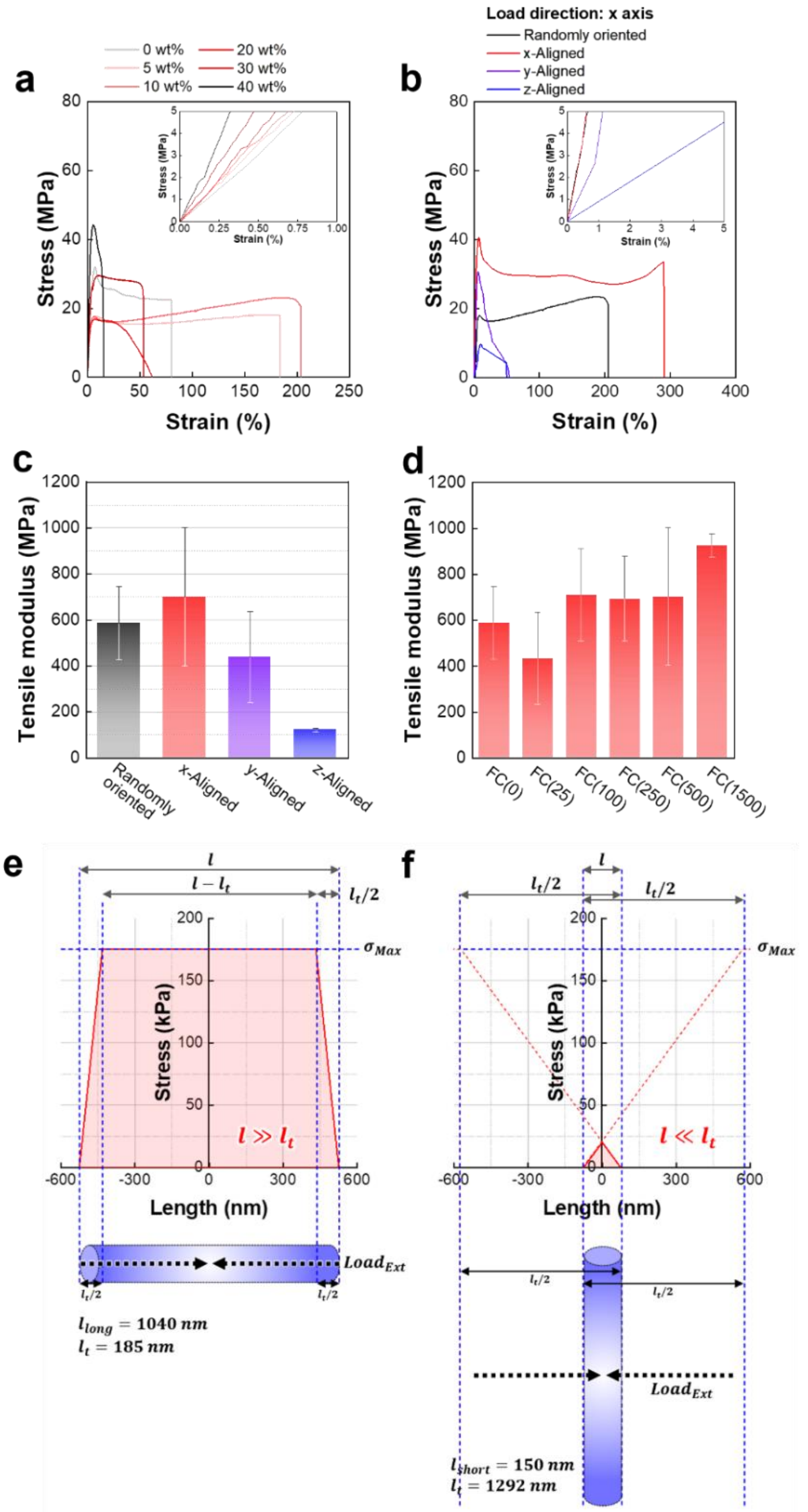

Figure S16. Mechanical characterization and stress transfer analysis. (a,b) Engineering stress–strain curves of SMP/FBTO NW composites with varying filler loadings (0–40 wt%) (a) and different alignment states measured under uniaxial tension along the x-axis (b). The inset in (b) highlights the linear elastic region at low strains. (c,d) Comparison of tensile moduli for 10 wt% composites depending on the alignment orientation (c) and the magnetic field strength applied during solidification (d). (e,f) Theoretical modeling of load transfer and stress confinement efficiency along the longitudinal (long) axis (e) and transverse (short) axis (f) of a single BTO NW, derived from load transfer theory (see Supplementary Note 2 for details).

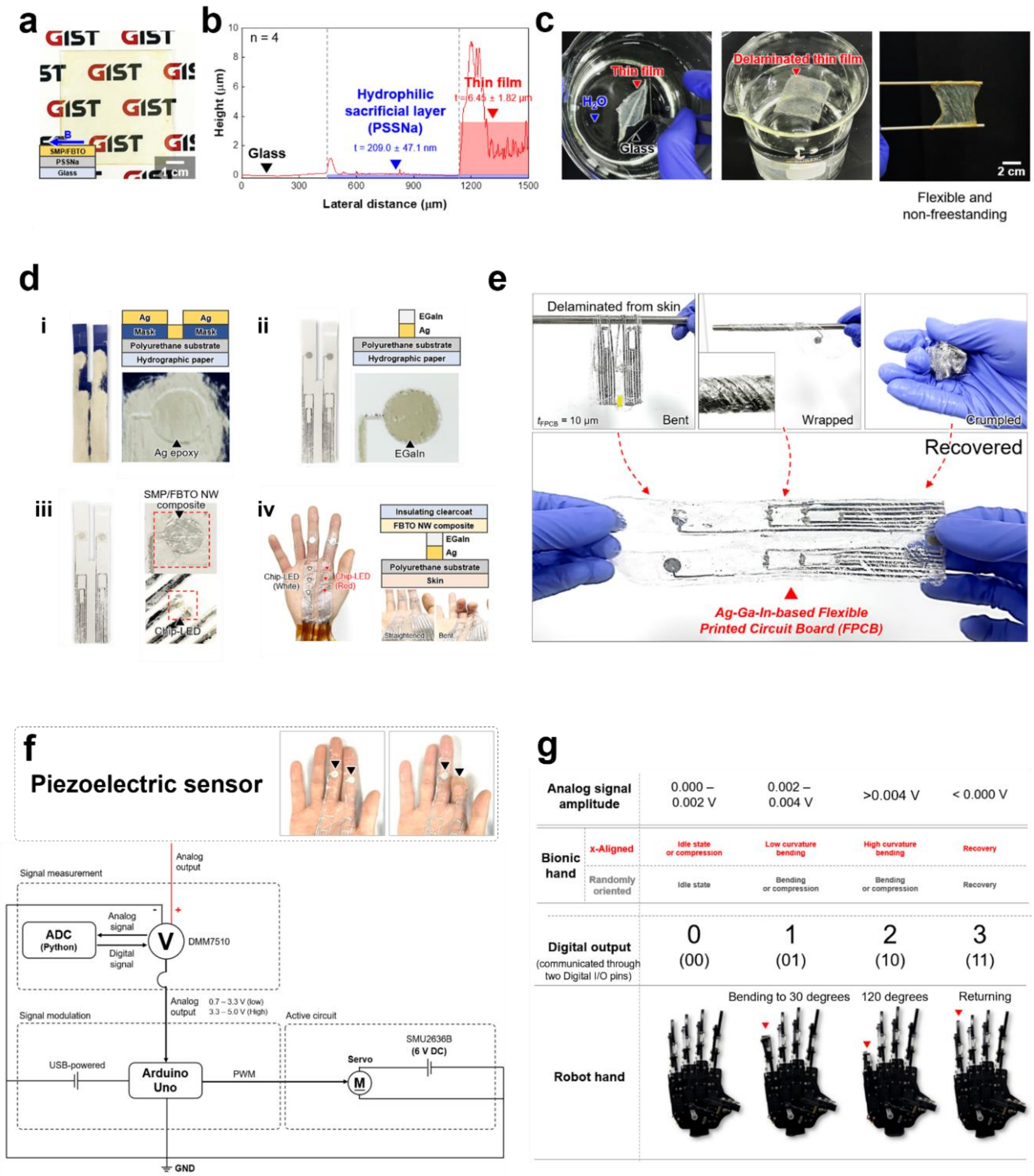

Figure S17. Fabrication and system integration of ultrathin wearable interfaces. (a–c) Preparation of ultrathin (~6 μm) SMP/FBTO NW composite films. (a) Optical image of the film cast on a water-soluble sacrificial layer. (b) Surface height profile confirming the film thickness. (c) Delamination process via dissolution of the sacrificial layer in water. (d) Schematic workflow and photographs illustrating the fabrication of skin-conformal wearable sensors. (e) Flexibility demonstration of the Ag–Ga–In-based flexible printed circuit board (FPCB). (f) Circuit diagram of the signal processing interface for the remote-controlled robotic hand. (g) Control logic thresholds defined for robotic actuation: (i) idle state (0–2 mV), (ii) partial bending (30º, 2–4 mV), (iii) full bending (120º, >4 mV), and (iv) return/reset (<0 mV).

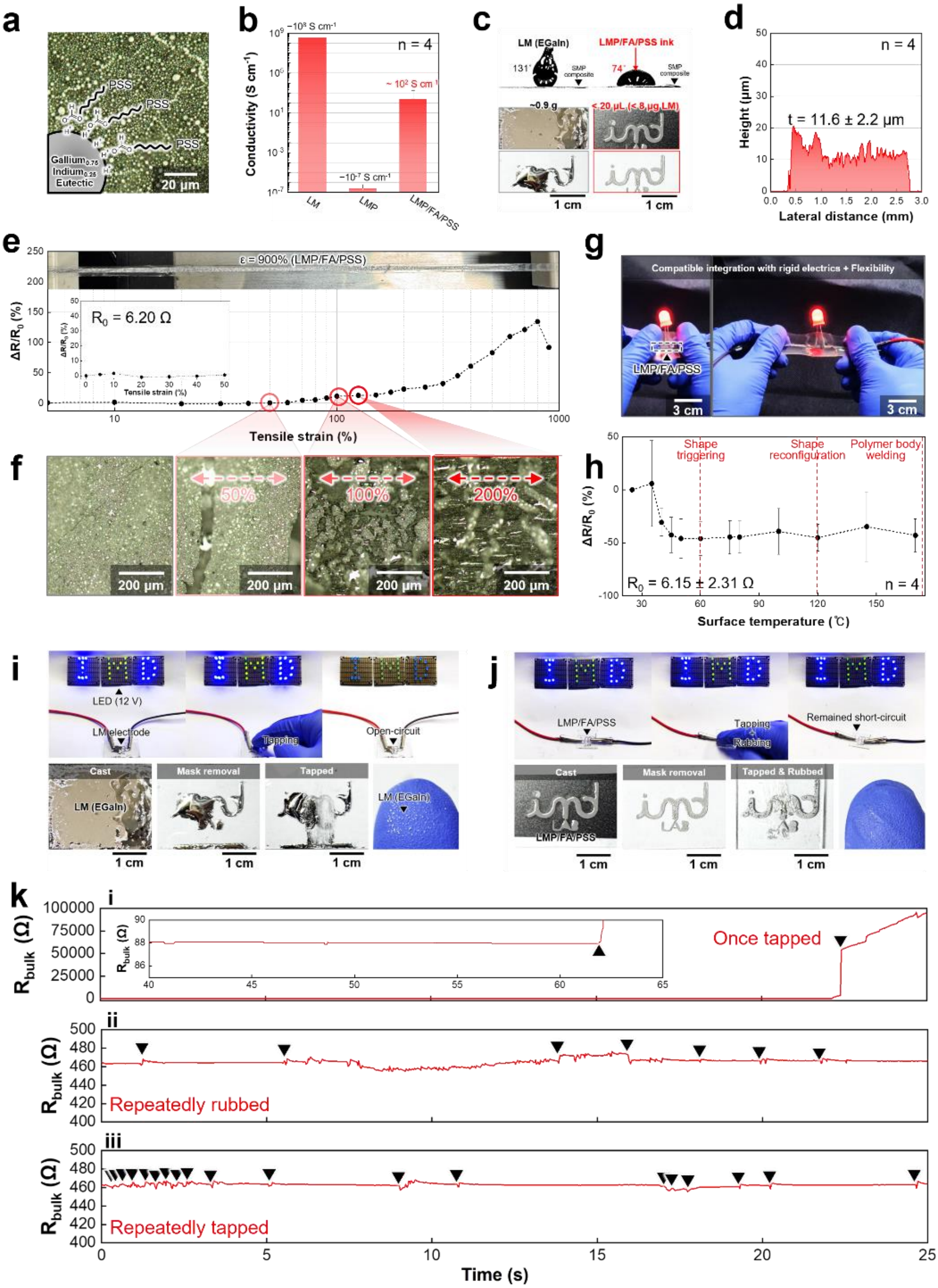

Figure S18. Characterization and stability of the liquid-metal-based stretchable ink. (a) Optical micrograph of the formulated ink comprising EGaIn microparticles (LMP), formic acid (FA), and poly(sodium 4-styrenesulfonate) (PSS). Inset: Schematic illustration of the proposed

surface chemical interactions. (b,c) Comparative analysis of (b) electrical conductivity and (c) surface contact angle for pristine liquid metal (LM), untreated particles (LMP), and the functionalized ink (LMP/FA/PSS). (d) Surface height profile of a printed electrode. (e,f) Electromechanical performance under tensile strain: relative resistance change (e) and corresponding morphological evolution (f) on a VHB substrate. (g) Integration capability with rigid electronic components. (h) Thermal stability of the electrode resistance across the robotic processing temperatures, marked by red dotted lines: shape triggering (60 ℃), reconfiguration (120 ℃), and interface welding (175 ℃). (i–k) Mechanical robustness assessment. Comparison of physical stability during handling for pristine LM (i) versus the functionalized ink (j). (k) Resistance monitoring under mechanical abrasion (rubbing and tapping), highlighting the enhanced durability of (ii,iii) the LMP/FA/PSS electrode compared to (i) the unstable pristine LM .

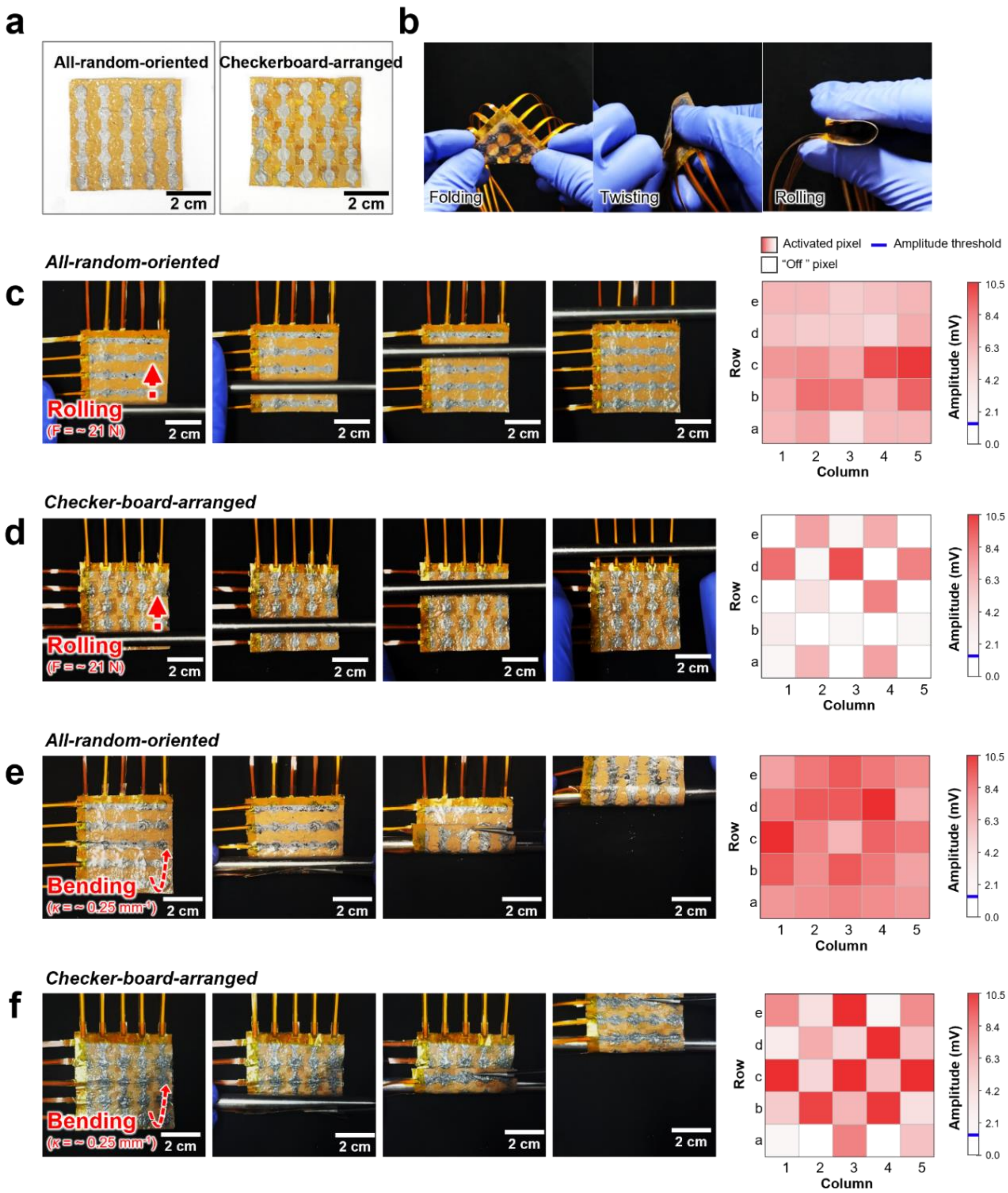

Figure S19. Comparison of spatiotemporal sensing capabilities. (a,b) Device configuration and mechanics. (a) Optical photographs contrasting the control array with all-randomly oriented pixels (left) and the proposed checkerboard array with alternating x- and z-aligned pixels (right). (b) Demonstration of the mechanical flexibility of the as-fabricated device. (c–f) Spatiotemporal piezoelectric voltage (V_{oc}) mapping under dynamic stimuli. The 5 × 5 matrix plots compare the response of the random control array (c,e) versus the aligned checkerboard array (d,f) under two distinct scenarios: (c,d) sequential compression induced by a cylinder rolling across the surface, and (e,f) sequential bending deformation conforming to the cylinder. Data are presented as superimposed time-lapse intensity maps to visualize the stimulus trajectory and sequence.

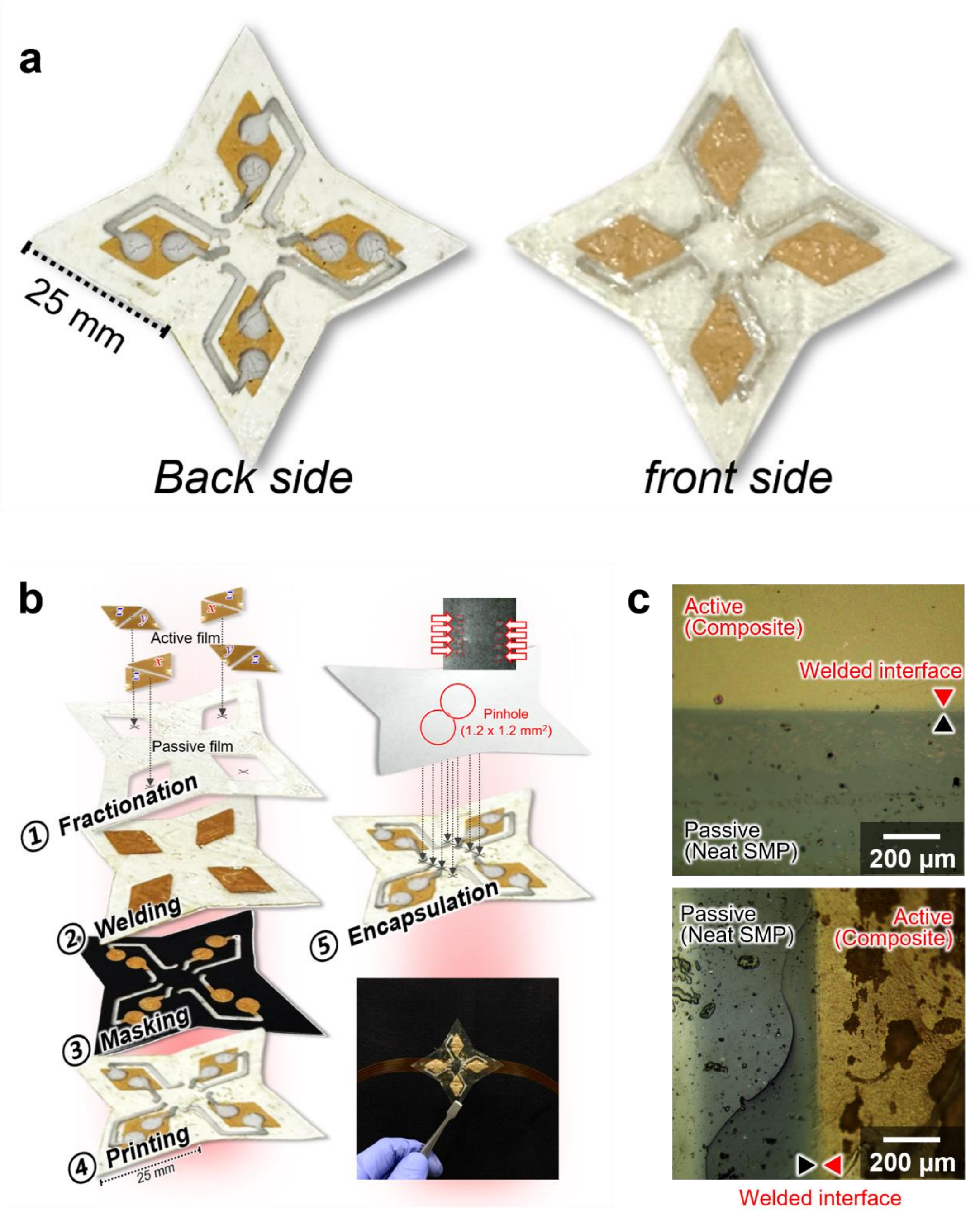

Figure S20. Fabrication and structural integration of the monolithic soft robot. (a) Optical photograph of the as-prepared monolithic soft robot. (b) Schematic illustration of the layer-by-layer fabrication process. (c) Optical micrographs characterizing the welded interface between the passive SMP template and the active composite unit, confirming seamless structural integration.

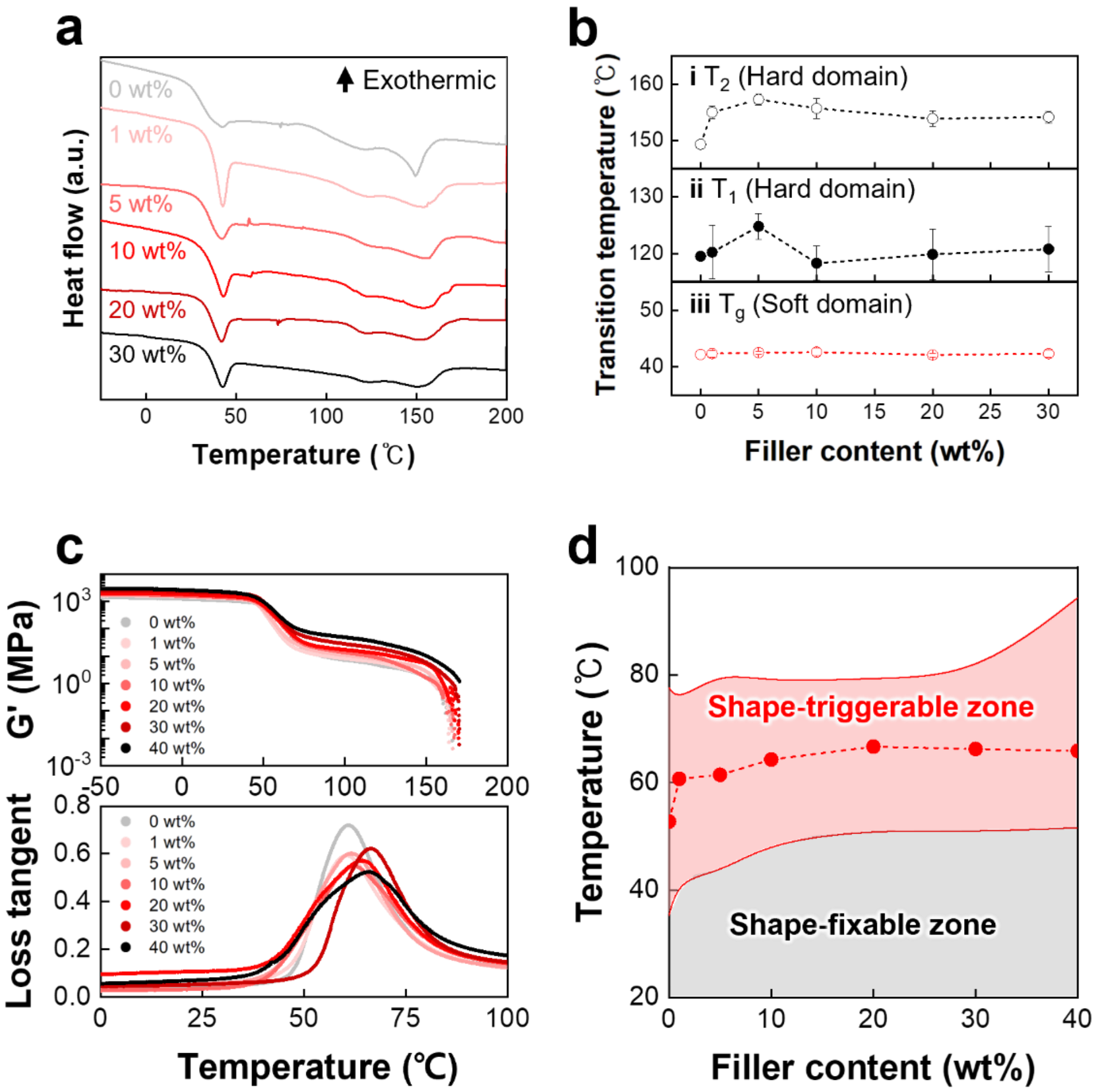

Figure S21. Thermal phase transitions and shape-memory performance. (a) Differential scanning calorimetry (DSC) thermograms of SMP/FBTO NW composites with varying filler loadings (0–30 wt%). (b) Identification of characteristic transition temperatures: T_2 (melting of long-range ordered hard domains acting as physical crosslinks), T_1 (melting of short-range ordered hard segments), and T_g (glass transition of amorphous soft segments). (c,d) Thermomechanical characterization of composites with filler loadings extended to 40 wt%. (c) Dynamic mechanical analysis (DMA) spectra. (d) Quantitative evaluation of thermal shape-memory behaviors.

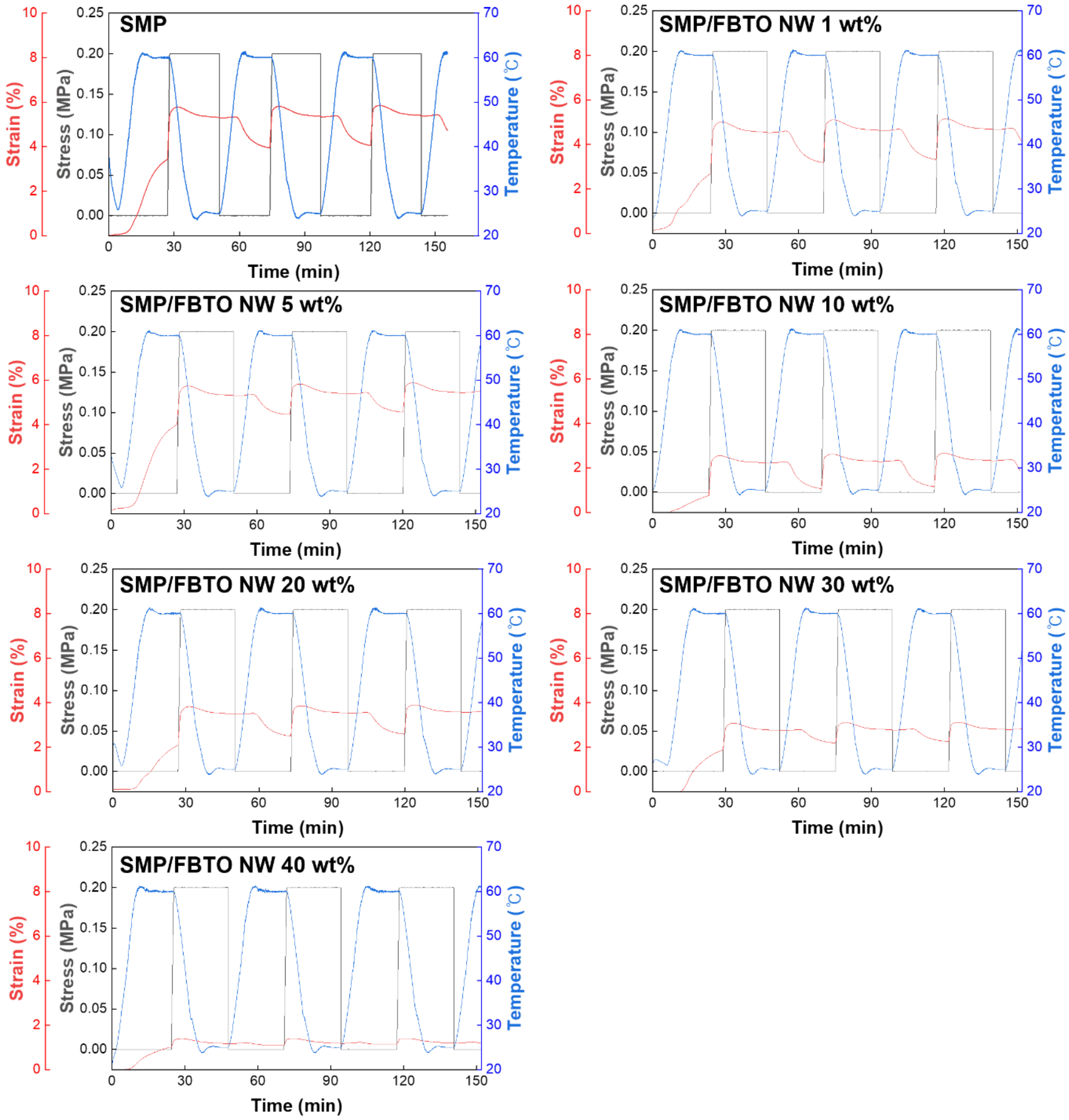

Figure S22. Shape-memory characterization via controlled-force mode tests in DMA. Representative DMA profiles obtained in controlled-force mode for SMP/FBTO NW composites with varying filler loadings (0–40 wt%), demonstrating the mechanical response under thermal cycling.

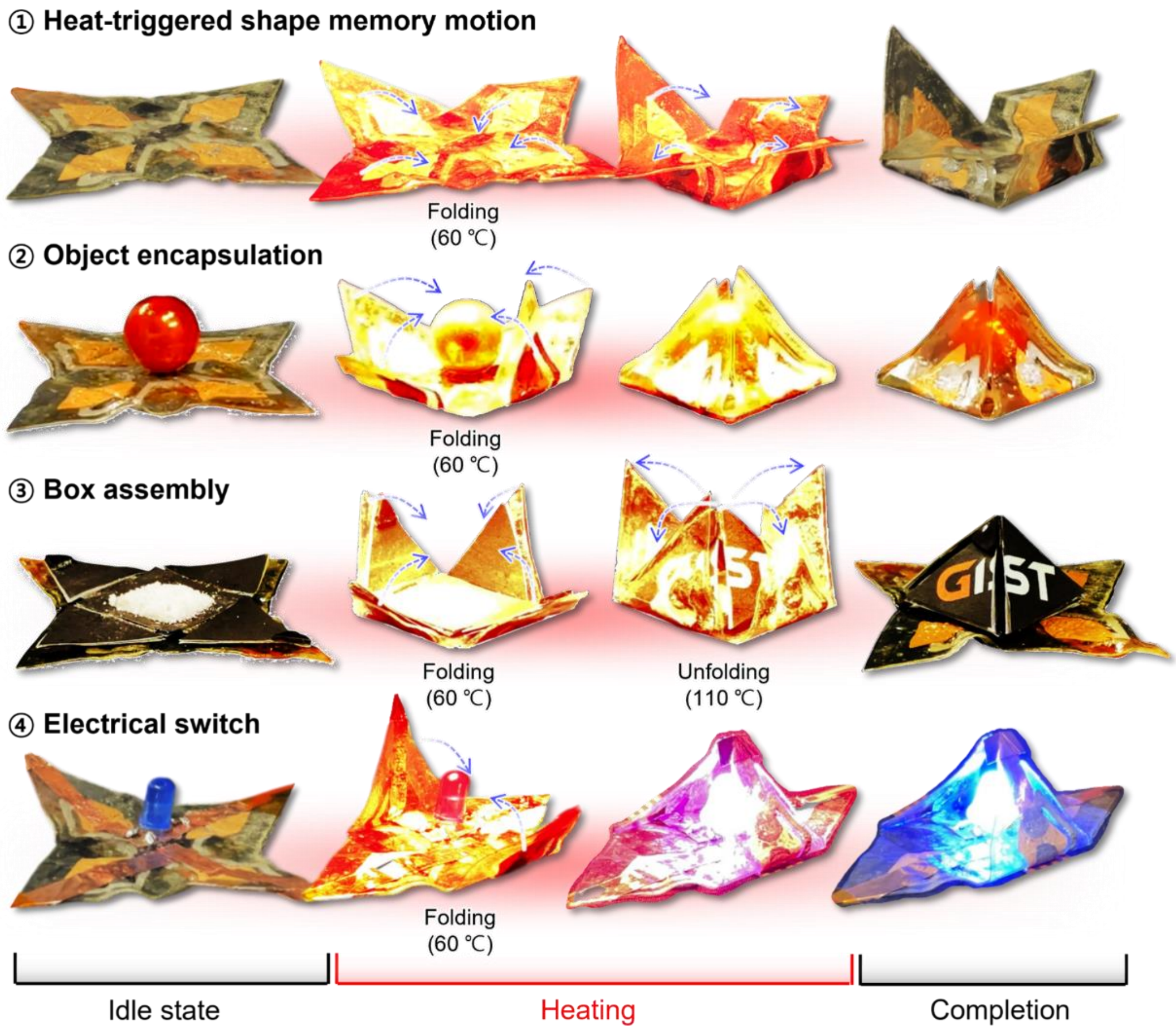

Figure S23. Versatile operational modes of the monolithic soft robot. Demonstrations of diverse actuation capabilities: ① complex origami folding into a swan configuration, ② object encapsulation, ③ self-folding box assembly, and ④ functional operation as an electrical switch to activate an LED.

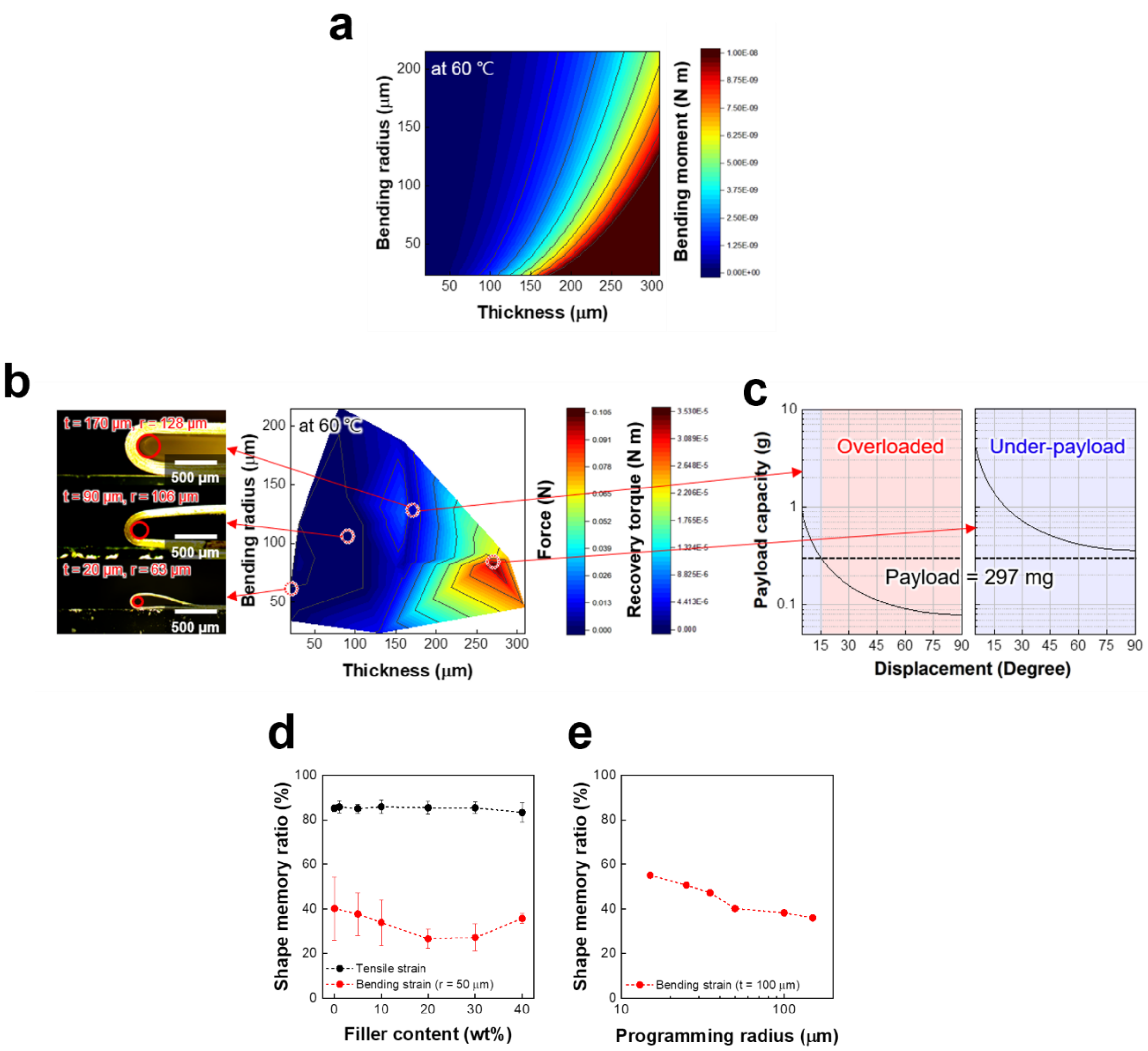

Figure S24. Mechanics of actuation and shape-memory performance. (a) Theoretical modeling of the bending moment as a function of film thickness and bending radius. (b,c) Validation and application of the model: experimental verification of theoretical predictions (b) and numerical estimation of the payload capacity for SMP beams (c). (d,e) Evaluation of shape-memory recovery performance depending on (d) filler loadings (0–40 wt%) and (e) the programmed bending radius (15–150 μm).

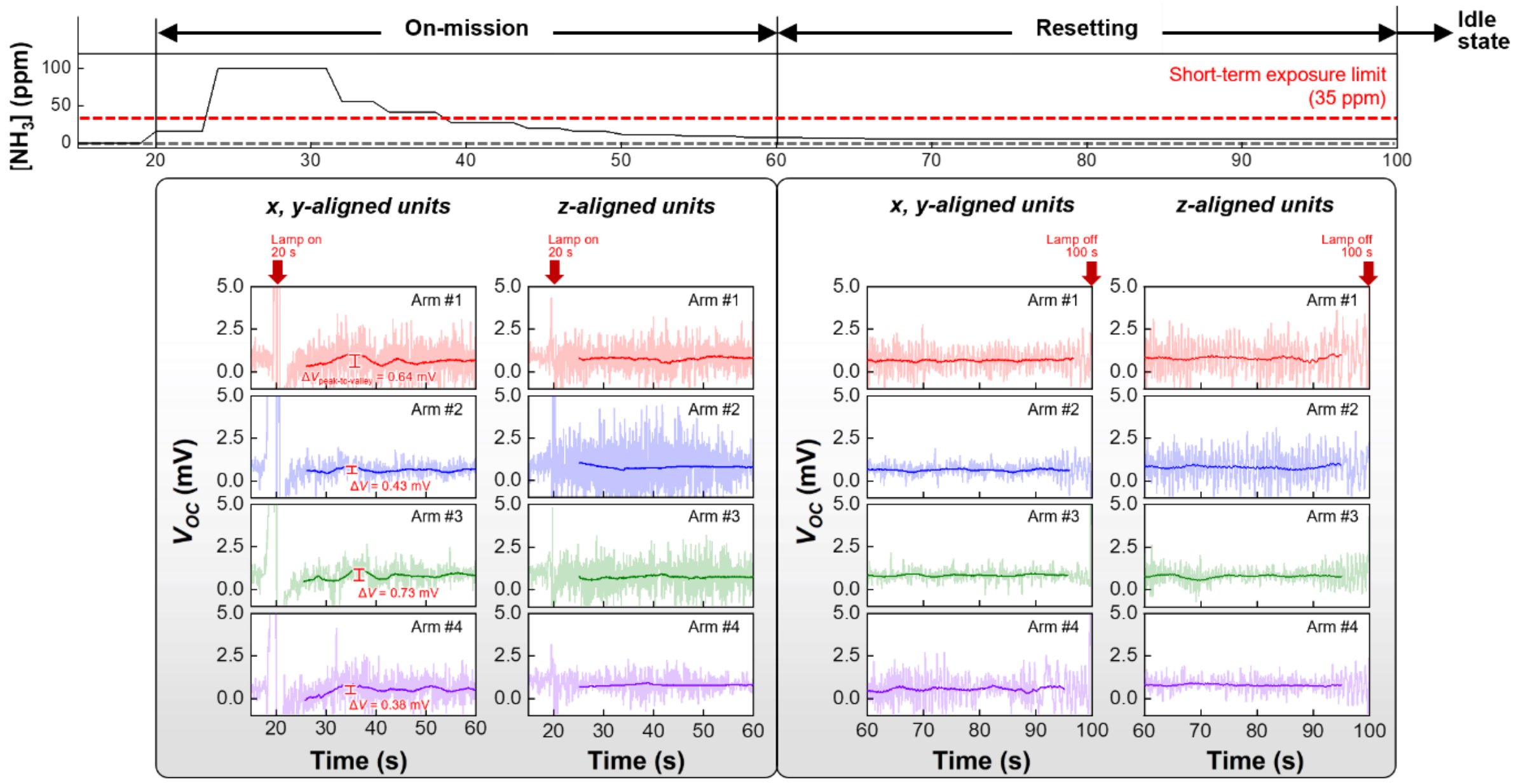

Figure S25. Robotic encapsulation and operando monitoring. Application of the soft robot for self-folding box assembly to isolate a chemical contaminant. Simultaneous acquisition of NH_3 gas concentration profiles and operando mechanosensing signals during the robotic operation, verifying successful encapsulation and real-time state feedback.

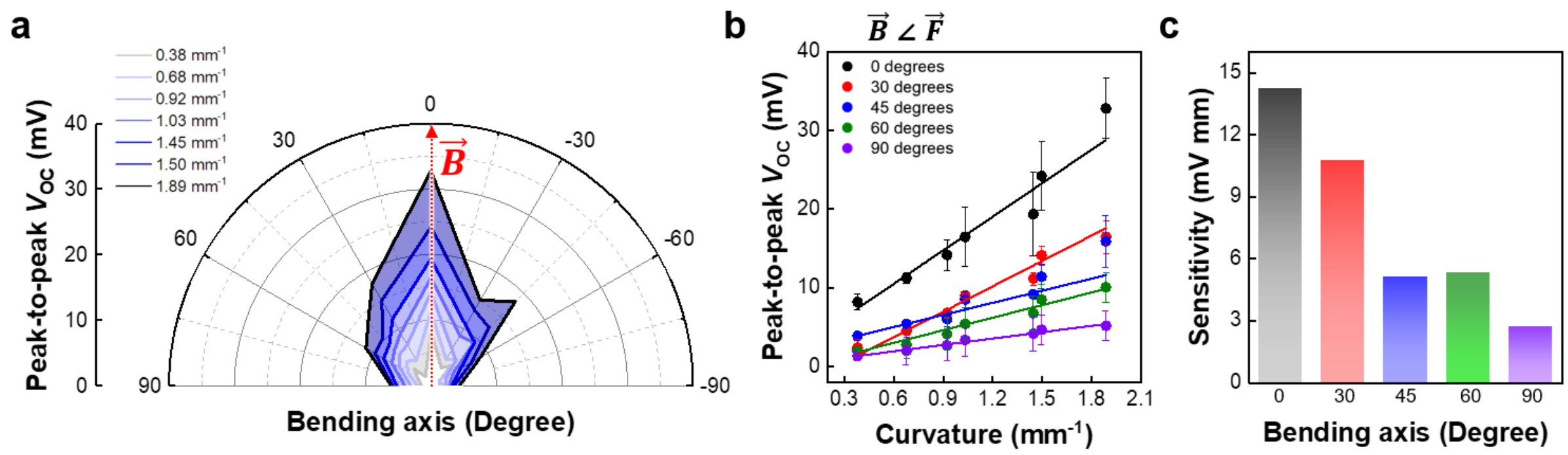

Figure S26. Piezoelectric response of the composite sensor evaluating vector-resolving capabilities under combined multiaxial loading. (a) A polar contour plot illustrating the peak-to-peak V_{oc} generated at various bending angles relative to the primary nanowire alignment axis ($\vec{B}$) under differing applied curvatures. (b) Linear correlation between the peak-to-peak V_{oc} and the applied bending curvature across multiple relative angles between the alignment axis and the loading vector ($\vec{B} \angle \vec{F}$) ranging from 0 to 90 degrees. (c) Extracted bending sensitivity as a function of the respective bending axis angle.

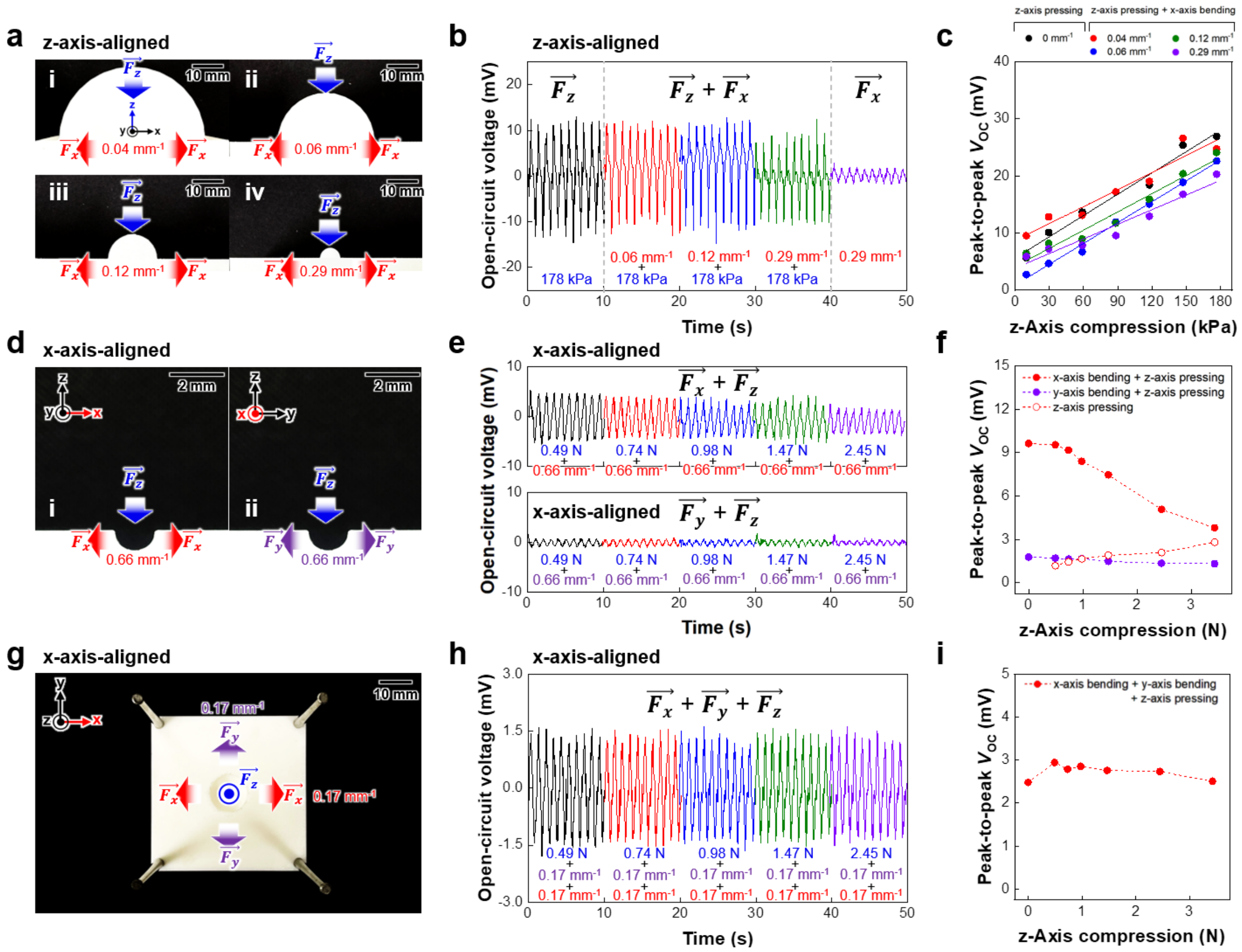

Figure S27. Piezoelectric response of the composite sensors examining the fidelities of signal decoupling under multi-axial mechanical environments. (a) Visual inspection of the testing method for the exteroceptive z-axis-aligned sensors under a dynamic pressing mode upon a static bending strain ($\vec{F_z}+\vec{F_x}$). Insets (i)–(iv) indicate the varying applied bending curvatures of 0.04, 0.06, 0.12, and 0.29 mm^{-1}, respectively. Corresponding (b) signal waveform profiles and (c) peak-to-peak amplitude plots evaluating the linear sensitivity to z-axis compression without destructive signal crosstalk. (d) Visual inspection of the proprioceptive x-axis-aligned sensors subjected to a dynamic bending strain (0.66 mm^{-1}) combined with dynamic compressive forces. Insets specify the bending configurations applied parallel to the (i) x-axis ($\vec{F_x}+\vec{F_z}$) and (ii) y-axis ($\vec{F_y}+\vec{F_z}$). Corresponding (e) open-circuit voltage waveform profiles and (f) peak-to-peak amplitude plots summarizing the signal decoupling performance, signal attenuation limits, and out-of-plane force distinction capabilities. (g) Visual inspection of the x-axis-aligned sensors under a highly complex 3D-axial dynamic mechanical environment consisting of simultaneous x- and y-axis bending alongside z-axis pressing ($\vec{F_x}+\vec{F_y}+\vec{F_z}$). Corresponding (h) signal waveforms and (i) peak-to-peak amplitude plots demonstrating robust vector-resolving stability under multi-axial deformation.

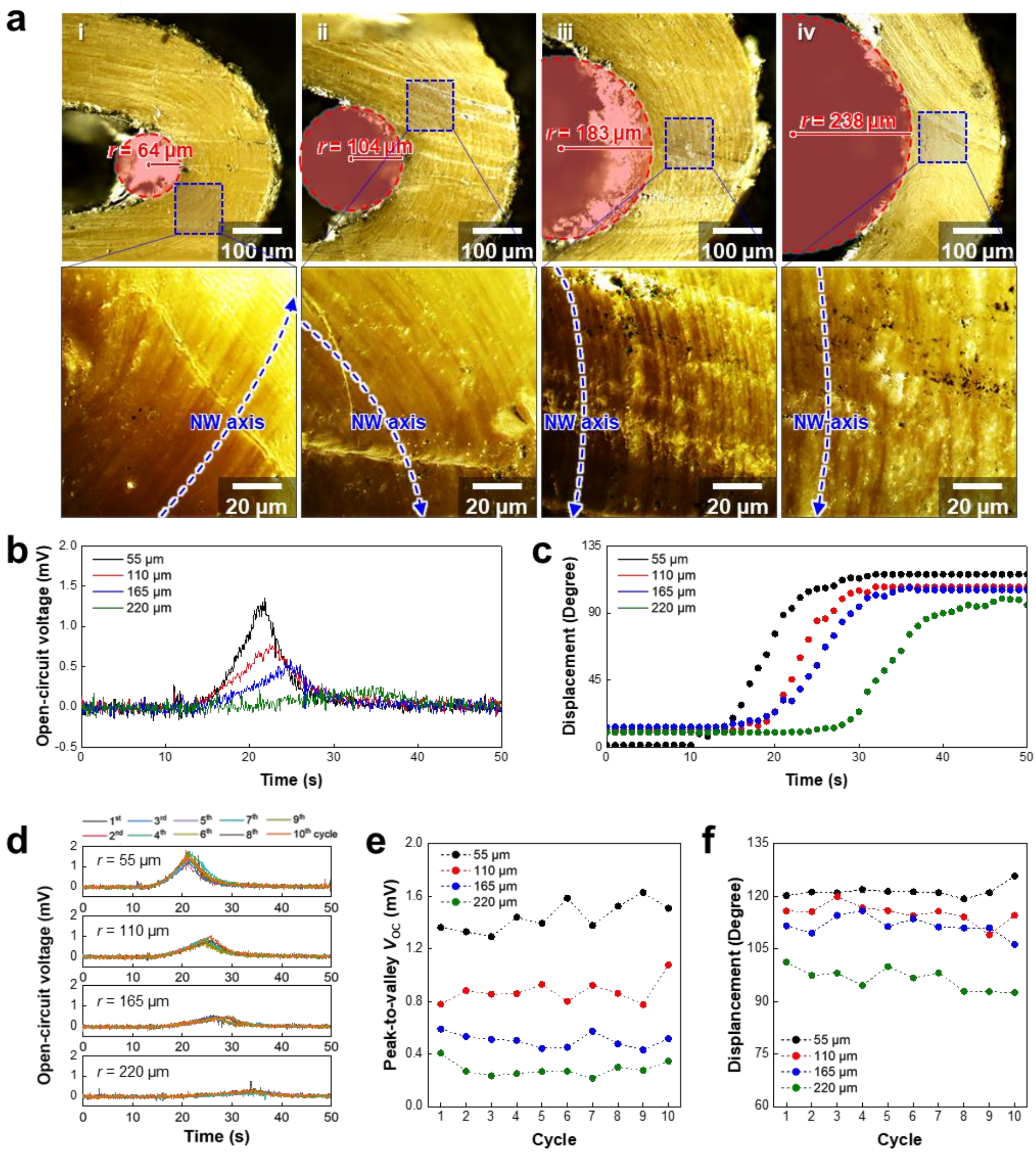

Figure S28. Intrinsic proprioceptive mechanics and cyclic reliability of the isolated monolithic sensor-actuator units. (a) OM images of the composite obtained at various programmed bending radii (64, 104, 183, and 238 μm) after subjecting the samples to 10 repeated thermo-mechanical actuation cycles. The magnified views clearly illustrate that the primary parallel alignment axes of the FBTO nanowires are highly preserved without spatial disorientation despite the repeated thermal modulus transitions and localized mechanical strains. (b) Time-domain V_{OC} and (c) macroscopic displacement of the isolated units programmed with varying bending radii (55, 110, 165, and 220 μm). (d) Continuous V_{OC} responses of the respective monolithic units under repeated actuation cycles. (e) Extracted peak-to-valley V_{OC} and (f) corresponding bending displacements over the continuous cycles.

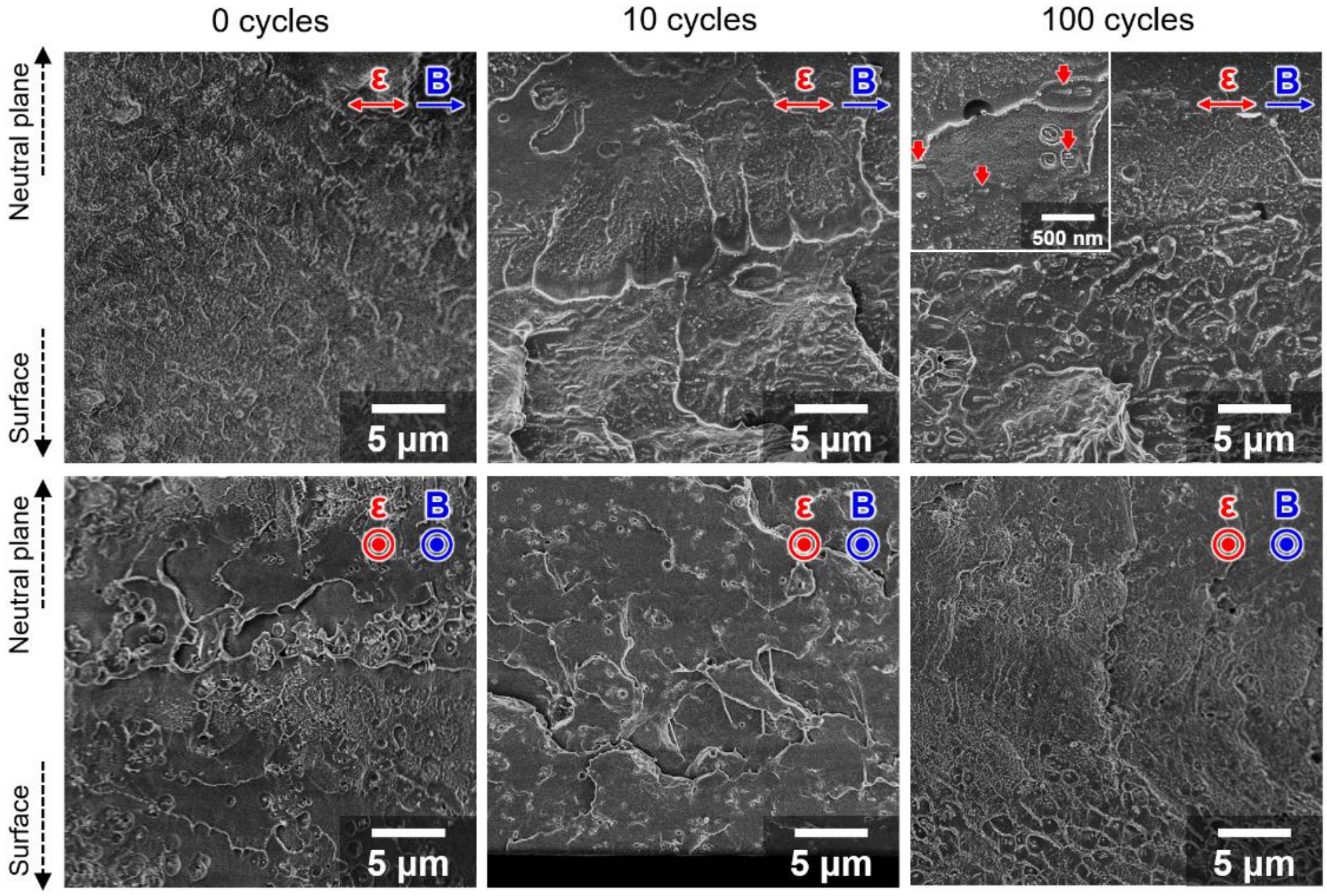

Figure S29. Cross-sectional SEM analysis of the composite under accelerated large-strain bending fatigue. Sequential images illustrate the microstructural evolution of the composite cross-sections acquired at 0, 10, and 100 cycles of accelerated degradation testing. The upper and lower panels display distinct cross-sectional viewing planes oriented parallel and perpendicular to the principal strain (ε) and magnetic alignment (B) axes. A magnified inset at the 100-cycle stage highlights the functionalized nanowires remaining stably embedded and maintaining their programmed alignment.

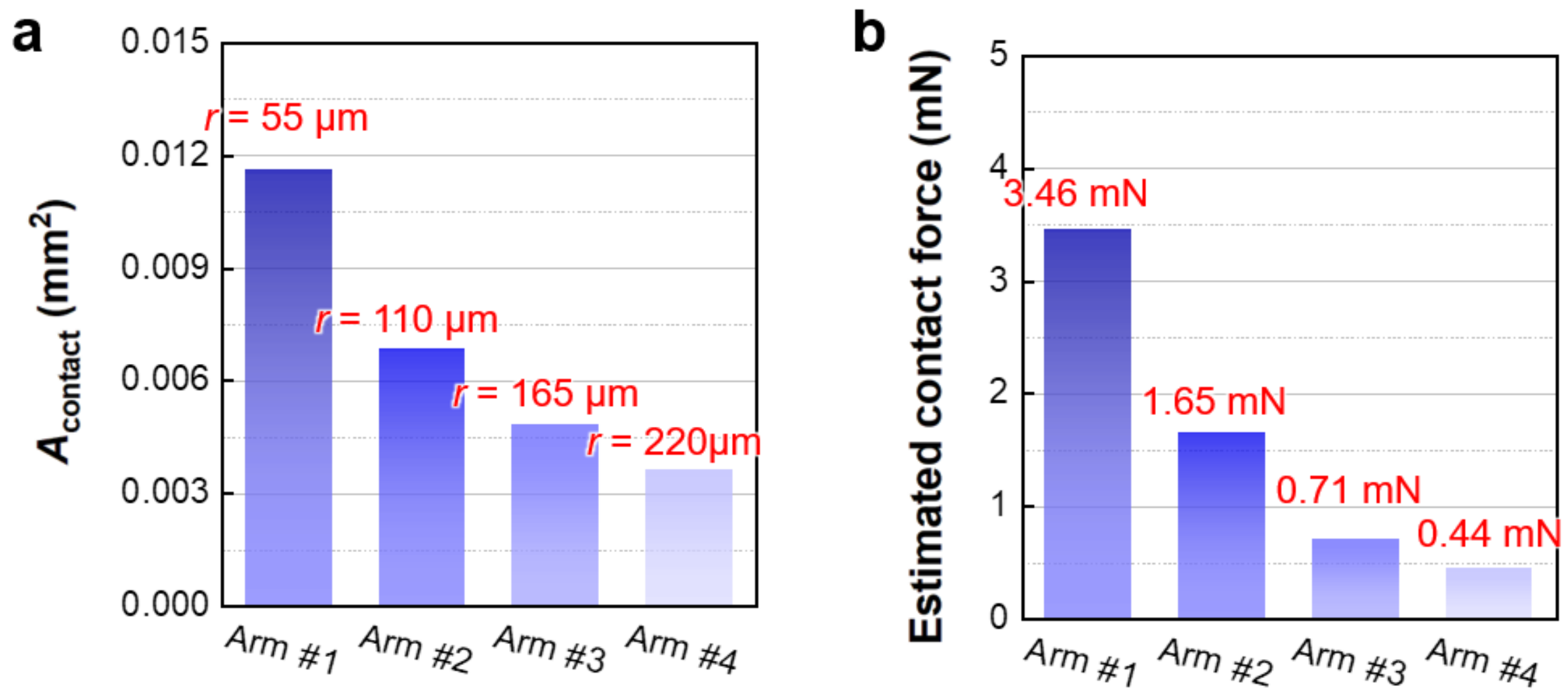

Figure S30. Quantitative estimation of localized contact mechanics and forces. (a) Analytically derived localized contact area ($A_{contact}$) between the compliant inner face of each robotic arm and the rigid spherical orb. The areas are estimated based on the Hertzian sphere-on-flat contact model, demonstrating a clear dependency on the programmed bending radii (r = 55, 110, 165, and 220 μm) of the individual arms. (b) Estimated localized contact forces exerted by the four robotic arms.

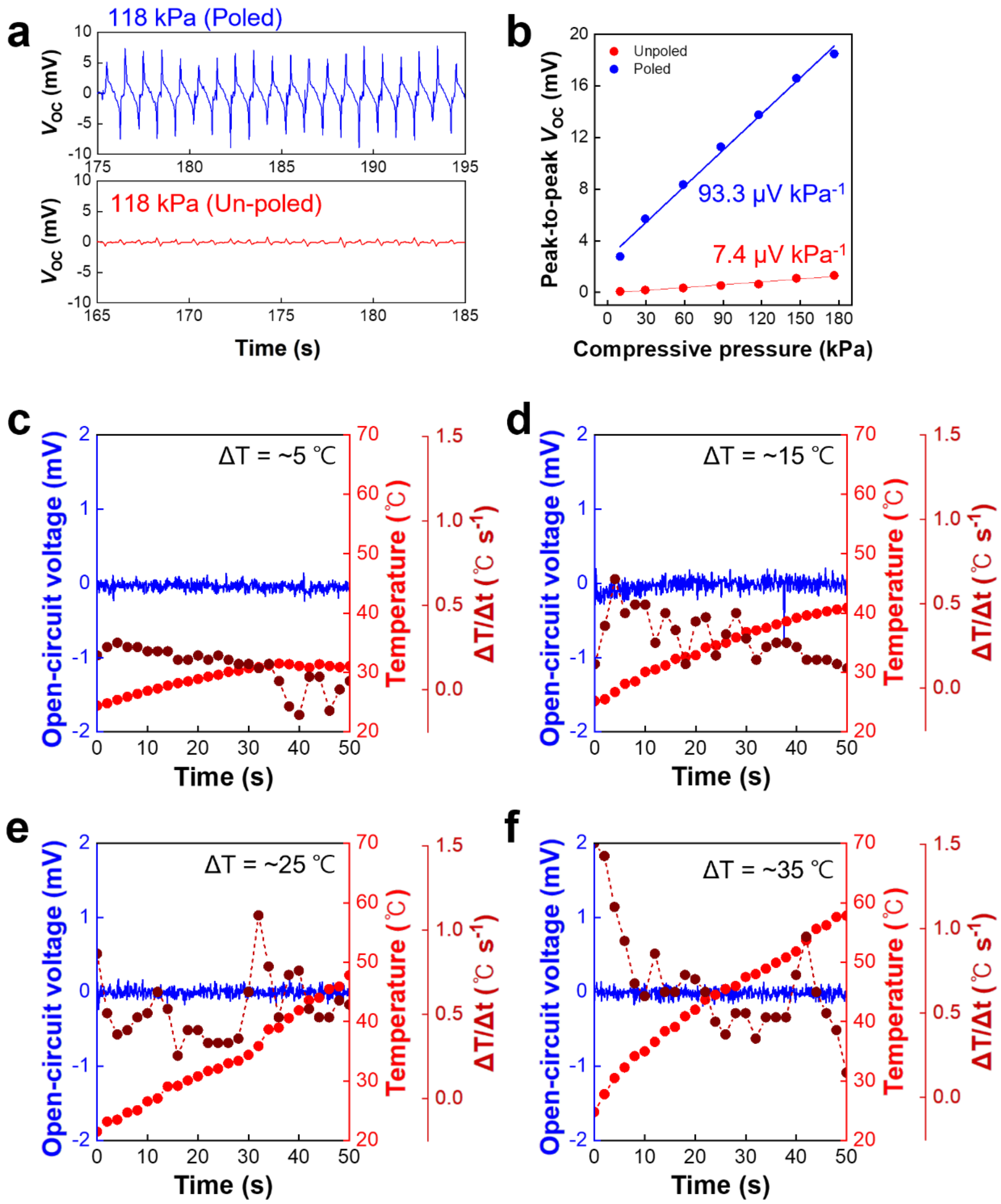

Figure S31. Verification of the piezoelectric origin of the generated signals. (a) Transient V_{oc} profiles and (b) the corresponding peak-to-peak V_{oc} amplitudes of unpoled and poled composite sensors subjected to cyclic compressive pressures. Control experiments evaluating pyroelectric contributions. (c–f) Transient V_{oc} outputs and corresponding temperature profiles of unprogrammed monolithic units under various targeted thermal variations (ΔT) of approximately (c) 5 °C, (d) 15 °C, (e) 25 °C, and (f) 35 °C. The devices under the tests reveal no noticeable voltage peaks across the evaluated heating rates (dT/dt).

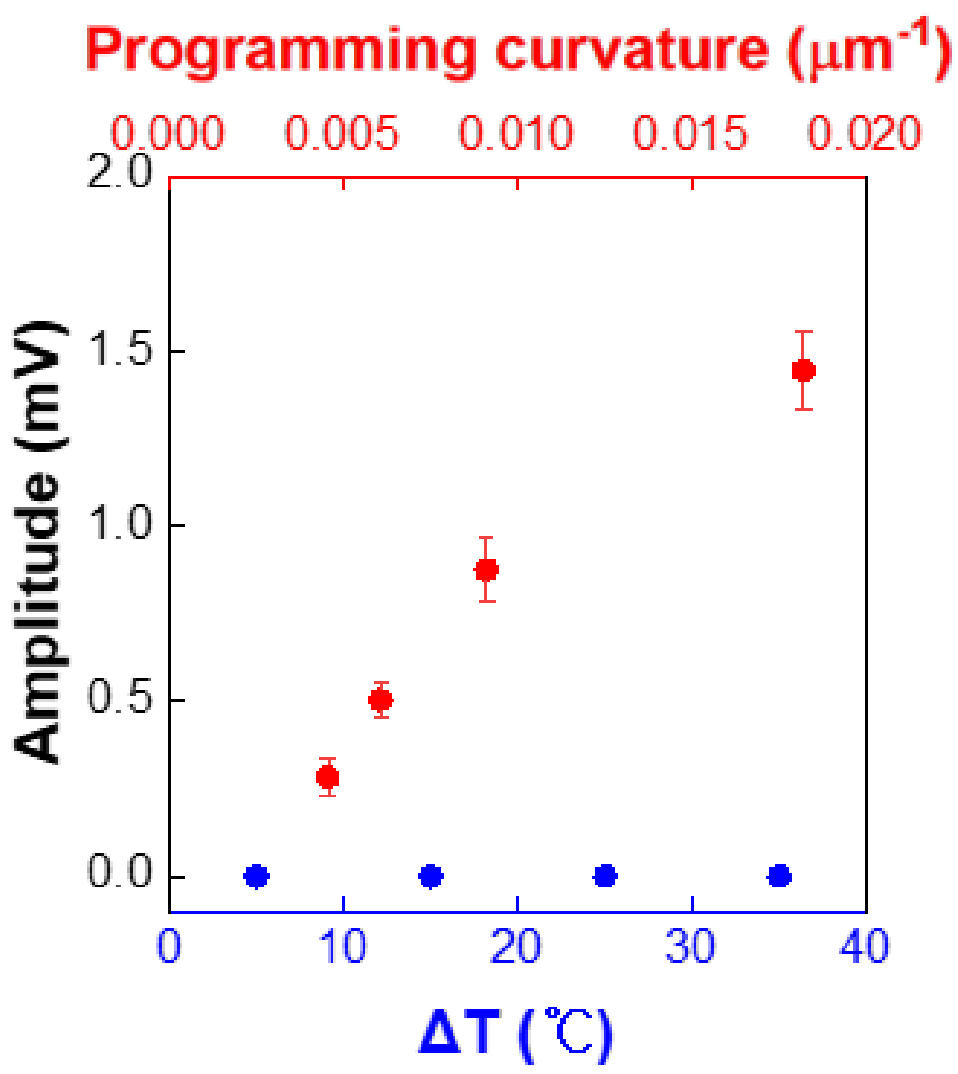

Figure S32. Piezoelectric response dependency on programmed curvature. Extracted V_{oc} peak amplitudes as a function of the targeted thermal variation (ΔT, blue marks) and the programmed curvature (red marks).

References

[1] R. M. Erb, R. Libanori, N. Rothfuchs, et al., "Composites Reinforced in Three Dimensions by Using Low Magnetic Fields," *Science* 335, (2012): 199. https://doi.org/10.1126/science.1210822

[2] L. Dilandro, A. T. Dibenedetto, J. Groeger, "The Effect of Fiber-Matrix Stress Transfer on the Strength of Fiber-Reinforced Composite Materials," *Polymer Composites* 9, (1988): 209. https://doi.org/10.1002/pc.750090308

[3] V. L. Popov, M. Heß, E. Willert, *"Handbook of Contact Mechanics"*. Springer, **2019**.
